# Metabolic flux analysis in *Ashbya gossypii* using ^13^C-labeled yeast extract: industrial riboflavin production under complex nutrient conditions

**DOI:** 10.1186/s12934-018-1003-y

**Published:** 2018-10-16

**Authors:** Susanne Katharina Schwechheimer, Judith Becker, Lindsay Peyriga, Jean-Charles Portais, Christoph Wittmann

**Affiliations:** 1 Institute of Systems Biotechnology, Saarland University, Campus A1.5, 66123 Saarbrücken, Germany; 2 Université de Toulouse, INSA, UPS, INP, Toulouse, France; 3 INRA, UMR792 Ingénerie des Systèmes Biologiques et des Procédés, Toulouse, France; 4 CNRS, UMR5504, Toulouse, France

**Keywords:** *Ashbya gossypii*, Riboflavin, Vitamin B_2_, ^13^C tracer, Metabolic flux, Yeast extract, Industrial process

## Abstract

**Background:**

The fungus *Ashbya gossypii* is an important industrial producer of the vitamin riboflavin. Using this microbe, riboflavin is manufactured in a two-stage process based on a rich medium with vegetable oil, yeast extract and different precursors: an initial growth and a subsequent riboflavin production phase. So far, our knowledge on the intracellular metabolic fluxes of the fungus in this complex process is limited, but appears highly relevant to better understand and rationally engineer the underlying metabolism. To quantify intracellular fluxes of growing and riboflavin producing *A. gossypii*, studies with different ^13^C tracers were embedded into a framework of experimental design, isotopic labeling analysis by MS and NMR techniques, and model-based data processing. The studies included the use ^13^C of yeast extract, a key component used in the process.

**Results:**

During growth, the TCA cycle was found highly active, whereas the cells exhibited a low flux through gluconeogenesis as well as pentose phosphate pathway. Yeast extract was the main carbon donor for anabolism,  while vegetable oil selectively contributed to the proteinogenic amino acids glutamate, aspartate, and alanine. During the subsequent riboflavin biosynthetic phase, the carbon flux through the TCA cycle remained high. Regarding riboflavin formation, most of the vitamin’s carbon originated from rapeseed oil (81 ± 1%), however extracellular glycine and yeast extract also contributed with 9 ± 0% and 8 ± 0%, respectively. In addition, advanced yeast extract-based building blocks such as guanine and GTP were directly incorporated into the vitamin.

**Conclusion:**

Intracellular carbon fluxes for growth and riboflavin production on vegetable oil provide the first flux insight into a  fungus on complex industrial medium. The knowledge gained therefrom is valuable for further strain and process improvement. Yeast extract, while being the main carbon source during growth, contributes valuable building blocks to the synthesis of vitamin B_2_. This highlights the importance of careful selection of the right yeast extract for a process based on its unique composition.

**Electronic supplementary material:**

The online version of this article (10.1186/s12934-018-1003-y) contains supplementary material, which is available to authorized users.

## Background

Riboflavin, also called vitamin B_2_, is an essential metabolite for humans and animals and therefore, must be obtained through diet. Riboflavin deficiency can result in skin lesions and corneal vascularization amongst other symptoms. Ariboflavinosis during pregnancy can lead to growth defects in newborns [1, 2]. In the cellular physiology, the vitamin serves as precursor for the cofactors FAD and FMN and thus, plays a central role in the metabolism [3, 4]. The majority of the continuously growing riboflavin market is used for feed fortification [5]. Luckily, the ability of microorganisms to synthesize the vitamin has meanwhile enabled production on an industrial level: more than two decades ago, the initially chemical synthesis of riboflavin was partially replaced by fermentation using the filamentous hemiascomycete *Ashyba gossypii* [5], which efficiently forms the vitamin on vegetable oil as major carbon source [6, 7]. Nowadays, the vitamin is exclusively produced via biotechnological synthesis [8].

**Fig. 1:**
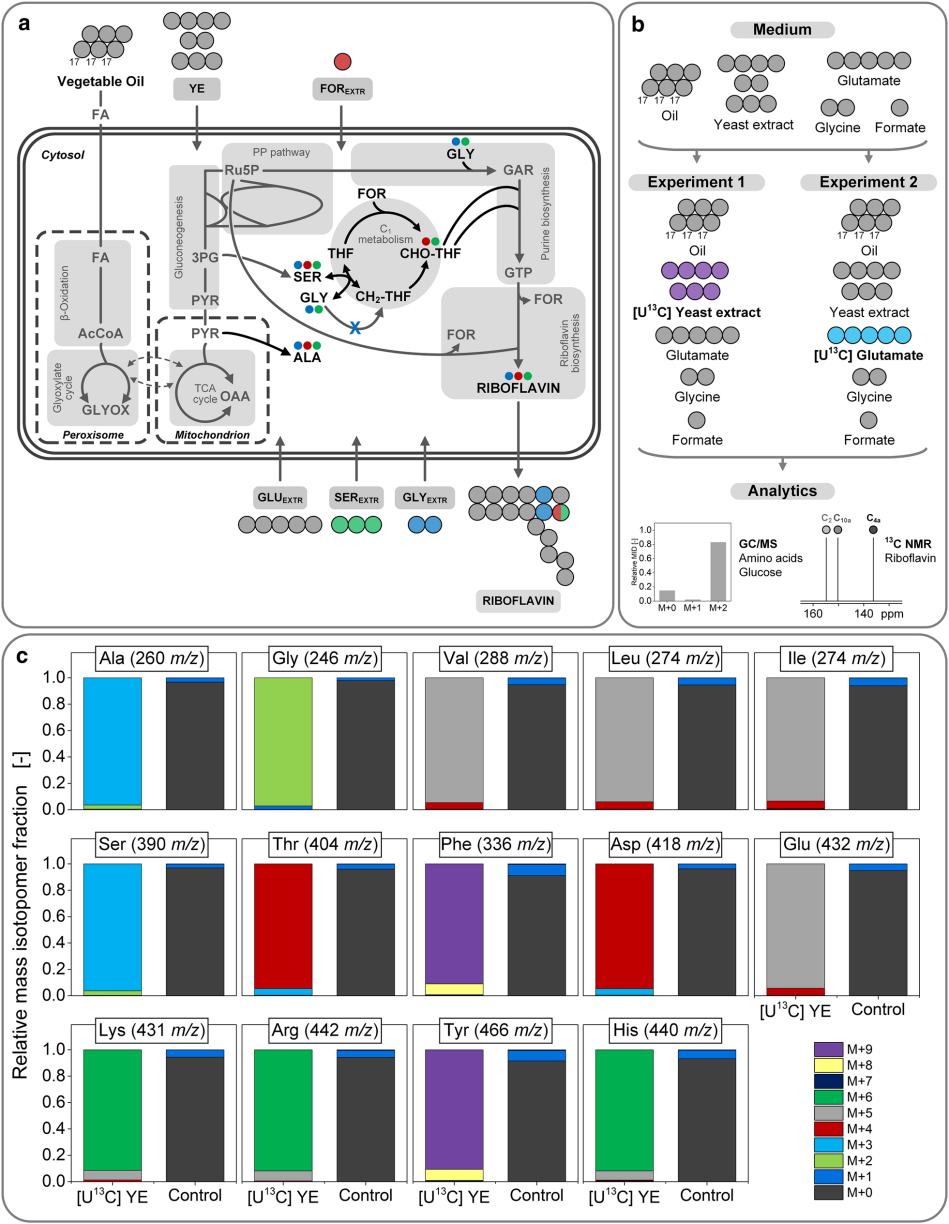
Schematic riboflavin biosynthesis from vegetable oil in *A.* *gossypii* under industrial process conditions (**a**). Riboflavin biosynthesis with *A.* *gossypii* entails multiple compartments and different metabolic pathways. The terminal biosynthesis starts from GTP, which is formed in the purine biosynthesis, and ribulose 5-phosphate originating from the pentose phosphate (PP) pathway. The map includes qualitative data from our previous study [16]: in three parallel ^13^C tracer experiments, the contribution of the respective tracer to growth as well as riboflavin biosynthesis was assessed. The ^13^C tracers used were glycine (blue), formate (red), and serine (green). All other carbon sources (vegetable oil, yeast extract, glutamate) remained naturally labeled (grey). The small circles indicate ^13^C contribution of the respective tracer to proteinogenic amino acids or riboflavin. Note that the size of the circles is not quantitative. The schematic presentation of the riboflavin molecule highlights the single carbon origin also in a qualitative manner: fully ^13^C-labeled glycine contributes to two distinct carbon atoms, while [3-^13^C] serine and [^13^C] formate contribute their ^13^C label to another carbon atom. Naturally labeled medium ingredients contribute to all carbon atoms in riboflavin. The one-carbon metabolism is only drawn in the cytosol. It can be assumed, however, that there is also a one-carbon metabolism in the mitochondrion. The ^13^C labeling strategy for the present work (**b**). In two parallel approaches, fully ^13^C-labeled yeast extract and glutamate replaced the naturally labeled substrates in an otherwise naturally labeled medium. Mass isotopomer distributions (MIDs) of the *t*-butyl-dimethylsilyl derivatized amino acids from the custom-synthesized and hydrolyzed [U^13^C] yeast extract (**c**). Naturally labeled amino acids served as control. The mass isotopomer M + 0 represents the relative amount of non-labeled, M + 1 the amount of singly-labeled mass isotopomer fraction and so on. All data are corrected for natural isotopes. Amino acids are designated with their three letter code. The error of the measurement was below 1%. 3PG, 3-phosphoglycerate; CH_2_-THF, 5,10-methylenetetrahydrofolate; AcCoA, acetyl-CoA; CHO-THF, 10-formyltetrahydrofolate; FA, fatty acid; FOR_(EXTR)_, (extracellular) formate; GAR, glycineamide ribonucleotide; GLU_EXTR_, extracellular glutamate; GLY_(EXTR)_, (extracellular) glycine; GTP, guanosine triphosphate; OAA, oxaloacetate; PYR, pyruvate; Ru5P, ribulose 5-phosphate; SER_(EXTR)_, (extracellular) serine; THF, tetrahydrofolate; YE, yeast extract

Industrial riboflavin biosynthesis with *A.* *gossypii* is a complex process both inside and outside the cell: a multi-compartment biosynthesis ranging from ß-oxidation of fatty acids in the peroxisome via the tricarboxylic acid (TCA) cycle in the mitochondria to terminal riboflavin biosynthesis in the cytosol meets a rich production medium with vegetable oil, yeast extract, and supplemented glycine and glutamate (Fig. 1) [9–12]. As means of resolving underlying metabolic routes, ^13^C tracer studies have proven to be a powerful strategy in the past decade [13]. However, only chemically defined media with one single carbon source are applicable to be studied by conventional ^13^C metabolic flux analysis (MFA) [14, 15]. Industrial, complex production media with several carbon sources require a much more refined labeling approach. Most recently, we were able to successfully resolve parts of the riboflavin biosynthetic pathway in the industrial producer strain *A.* *gossypii* B2 with sophisticated ^13^C tracer experiments (Fig. 1) [16]. Hereby, glycine, but also formate and serine were used as ^13^C tracer and the isotope experiments were analyzed with GC/MS, LC/MS as well as NMR. This revealed that glycine efficiently fueled the intracellular pool and was incorporated as a whole into riboflavin, while extracellular formate and serine contributed only slightly to riboflavin biosynthesis (Fig. 1). Valuable insights were gained in that study, however, the impact of the chosen ^13^C tracers was confined to small parts of the metabolism so that the full picture could not be resolved so far. Accordingly, the contribution of vegetable oil, the main carbon source, or yeast extract to the carbon core metabolism and subsequent riboflavin biosynthesis still remains to be elucidated. Since riboflavin production with *A.* *gossypii* uses large amounts of yeast extract as complex medium ingredient [5, 17] and almost all labeling studies with the fungus were conducted using the complex source [18–20], it seemed crucial to uncover its contribution to the vitamin. Furthermore, yeast extract is generally used as nitrogen source for industrial fermentation processes [21, 22], which added to the importance of understanding its role in this exemplary microbial vitamin production.

In this study, growth and riboflavin production with the industrial overproducer strain *A.* *gossypii* B2 were investigated using ^13^C isotope tracer experiments and a combination of GC/MS as well as NMR techniques. The resulting labeling patterns of proteinogenic amino acids, cellular glycogen as well as riboflavin were then integrated with ^13^C labeling data from a recent flux analysis of riboflavin producing *A.* *gossypii* [16]. For the first time, this enabled the quantitative calculation of carbon fluxes in (i) the growth phase, but also (ii) the riboflavin biosynthetic phase in a riboflavin production set-up. The resulting data provide novel insights into the metabolism of *A.* *gossypii* on vegetable oil and deliver new starting points for process optimization.

## Methods

### Strain and maintenance

The riboflavin overproducing strain *Ashbya gossypii* B2 was derived from the wild type strain ATCC 10895 and was kept as glycerol stock as previously described [16].

### Media

The solid sporulation agar plate medium, the pre-culture medium as well as the medium for the main culture were of complex composition and contained yeast extract in large amounts among other ingredients. The preparation of the media is described elsewhere [16]. In isotope experiments, naturally labeled ingredients were replaced by ^13^C-labeled tracers in equimolar amount. The used 98% ^13^C-enriched glutamic acid was obtained from Sigma-Aldrich (Taufkirchen, Germany). Fully ^13^C-labeled yeast extract was custom-synthesized (Ohly, Hamburg, Germany) and characterized, regarding ^13^C enrichment and suitability for riboflavin production as described below.

### Cultivation and sampling

Cultivation of *A.* *gossypii* as well as sampling were carried out as described before [16]. Briefly, sporulation agar plates were inoculated with mycelia from cryo cultures (30 °C, 120 h). The complex pre-culture was then inoculated with mycelia from those agar plates (40 h) and subsequently, the main culture was inoculated with 3.5% of pre-culture. All cultivations were carried out with 10% filling volume on a rotary shaker at 200 rpm, 30 °C, and 80% humidity (Multitron Pro, Infors, Bottmingen, Switzerland). Per data point, three shake flasks were sacrificed and evaporation of culture volume was taken into account [16].

### Quantification of substrates and products

Amino acids in the culture supernatant, filtered prior to analysis (0.2 µm, PES, Sarstedt, Nümbrecht, Germany), were quantified by HPLC using the internal standard α-aminobutyric acid [16, 23]. Residual rapeseed oil was quantified gravimetrically prior to cell dry weight (CDW) determination by extraction with hexane–isopropanol and subsequent evaporation of the solvent [16, 24]. Once, the residual oil was removed, CDW could also be measured gravimetrically through filtration of the culture broth, followed by drying and subsequent weighing using a moisture analyzer (HB43-S, Mettler Toledo, Columbus, OH, USA) [16, 24]. Riboflavin was quantified spectrophotometrically as previously described [16, 25].

### Mass isotopomer distribution analysis of amino acids

Prior to analysis by GC/MS, cell protein harvested at the end of the exponential growth phase (36 h) and [U^13^C] yeast extract were hydrolyzed with 6 M HCl [16]. Derivatization of the hydrolyzed biomass and yeast extract was carried out as described previously [16, 26]. Quantification of mass isotopomer distributions (MIDs) of the amino acids of biomass and yeast extract was carried out by GC/MS with helium as carrier gas [16, 27]. For all amino acids, the ion cluster [M-57] was measured [28]. In addition, the ion cluster [M-85] was measured for leucine and isoleucine. The obtained MIDs were corrected for natural isotopes and the fraction of naturally labeled amino acids from the inoculum [16, 29]. Additionally, the summed fractional labeling (SFL) was calculated from the corrected MIDs [30] (Additional file 1: Equation S1). The net incorporation of ^13^C label from a given tracer to the SFL of a target molecule, e.g. amino acids, also took into account the dilution of ^13^C labeling of a respective precursor through naturally labeled pre-culture medium (SFL_corr_) (see Additional file 1).

### Mass isotopomer distribution analysis of glycogen-derived glucose

The mass isotopomer distribution for glucose from cellular glycogen was measured with the same GC/MS system as described above. About 5 mg cells were harvested at the end of the exponential growth phase by centrifugation. Recovery of glycogen from biomass was carried out as follows: harvested cells were washed several times with deionized water and resuspended in 500 µL enzyme solution, containing 70 U mL^−1^ amyloglucosidase (Sigma-Aldrich) and 1.2 kU mL^−1^ lysozyme (0.02 M Tris HCl, pH 7.0) (Sigma-Aldrich). Subsequent hydrolysis occurred at 37 °C for 3 h. After cell debris removal via filtration (0.2 µm, Merck Millipore), the glucose concentration was determined enzymatically, using a d-glucose assay kit according to instructions by the manufacturer (R-Biopharm, Darmstadt, Germany). At least 40 µg glucose were then dried by lyophilization, followed by a two-step derivatization using first 50 µL 2% methoxylamine in pyridine (80 °C, 25 min) and then adding 50 µL *N,O*-bis-trimethylsilyl-trifluoroacetamide (80 °C, 30 min). Measurement of the obtained trimethylsilyl *O*-methyloxime derivative of glucose was carried out using the following temperature profile: 3 min/150 °C, temperature increase to 230 °C at a rate of 8 °C/min, temperature increase to 325 °C at a rate of 25 °C/min. The temperature of the inlet, the interface, and the quadrupole was set to 280 °C. All samples were first measured in scan mode to check for isobaric interference. The mass isotopomer distribution was then determined in technical duplicates using selection ion monitoring of the ion cluster [M-15], which represents all six carbon atoms of glucose [31]. The resulting MIDs were corrected as described above (see Additional file 1 for details).

### Quantification of positional ^13^C enrichment of riboflavin by ^13^C NMR and ^1^H NMR

The quantification of positional ^13^C enrichment of riboflavin was done using ^1^H and ^13^C NMR. Riboflavin was harvested and prepared for analysis as previously described [16]. All NMR spectra were recorded on a Bruker Ascend Avance III 800 MHz spectrometer (Bruker, Rheinstetten, Germany) with the Bruker TopSpin software for data analysis [32]. The settings for the NMR analysis of riboflavin are described elsewhere [16]. The relative ^13^C enrichment of every carbon atom of riboflavin was corrected for the natural ^13^C background and the dilution of ^13^C labeling of the respective ^13^C tracer resulting from naturally labeled pre-culture medium originating from the inoculum (see Additional file 1).

### Estimation of metabolic fluxes during growth

On the basis of ^13^C labeling data and interpreting the individual labeling information plus measured extracellular fluxes from the different parallel ^13^C tracer studies, carbon fluxes were calculated with respect to rapeseed oil as major carbon source. The definition of the metabolic network is given in the supplement, the derived metabolite balances and the calculation leading to the final flux distribution are all described below.

For that, the exact demand of anabolic precursors for cell growth had to be derived. To a large extent, the biomass composition for *A.* *gossypii* was accessible from literature data: the genome-scale metabolic model of *A.* *gossypii* provided most of the information for the biomass composition of the fungus [33]. The cellular demand for acetyl–CoA and glyceraldehyde 3-phosphate, the building blocks of lipids, had to be adjusted to growth on vegetable oil. While growth on glucose leads to a lipid content of about 0.08–0.15 g g_CDW_^−1^, growth on vegetable oil results in a lipid content of 0.22 g g_CDW_^−1^ [24]. The precursor demand for riboflavin was derived from the literature and KEGG via the underlying pathway stoichiometry [34–37]. The resulting values for the eleven anabolic precursors are listed in Table 1. As described below, biomass constituents  were derived via de-novo synthesis and via uptake from medium components. This issue was resolved by the help of  ^13^C labeling data, which yielded the final demand for growth under the complex conditions. For this purpose, the uptake of each amino acid was calculated by addition of the respective SFL_corr_ for that particular amino acid from the single isotope experiments ([U^13^C] yeast extract and [^13^C_5_] glutamate conducted in this study as well as [^13^C_2_] glycine and [^13^C] formate completed in a previously published work [16]) (Additional file 1: Tables S2, S3). The remaining fraction of the amino acid was then attributed to de novo synthesis from vegetable oil. Each cellular building block, e.g. amino acids or nucleotides, is derived from one or more out of a total of eleven precursors (Additional file 1: Table S4). The demand for a single precursor is, therefore, the sum of the cellular demand of its building blocks. Pyruvate, e.g., is the metabolic precursor of alanine, valine, isoleucine, and leucine (Additional file 1: Table S4). In order to calculate carbon fluxes during the growth phase of *A. gossypii*, the following balances [Eqs. (1) to (29)] were formulated for the network depicted in Additional file 1: Fig. S3. This metabolic network represents growth on vegetable oil and yeast extract. Metabolite balances were expressed using the numbering of the fluxes as presented in Additional file 1: Fig. S2.

1 $${\text{FA}}_{\text{P}} \quad 0\, = \, 2 6v_{ 1} {-}v_{ 2}$$

2 $${\text{AcCoA}}_{\text{P}} \quad 0\, = \,v_{ 2} {-}v_{ 3} {-}v_{ 4} {-}v_{ 5} {-}v_{ 6}$$

3 $${\text{ICIT}}\quad 0\, = \,v_{ 6} {-}v_{ 7}$$

4 $${\text{GLYOX}} \quad 0\, = \,v_{ 7} {-}v_{ 5}$$

5 $${\text{SUC}}\quad 0\, = \,v_{ 7} {-}v_{ 8}$$

6 $${\text{MAL}}\quad 0\, = \,v_{ 5} \, + \,v_{ 8} {-}v_{ 6} {-}v_{ 9}$$

7 $${\text{OAA}}/{\text{MAL}} \quad 0\, = \,v_{ 9} \, + \,v_{ 20} {-}v_{ 10} {-}v_{ 2 1}$$

8 $${\text{AcCoA}}_{\text{M}} \quad 0\, = \,v_{ 4} {-}v_{ 10}$$

9 $${\text{CIT}} \quad 0\, = \,v_{ 10} {-}v_{ 1 1}$$

10 $${\text{AKG}} \quad 0\, = \,v_{ 1 1} \, + \,v_{ 1 3} {-}v_{ 1 2} {-}v_{ 20}$$

11 $${\text{GLU}}_{\text{INTR}} \quad 0\, = \,v_{ 1 2} \, + \,v_{ 1 4} {-}v_{ 1 3} {-}v_{ 1 5}$$

12 $${\text{OAA}} \quad 0\, = \,v_{ 2 1} {-}v_{ 2 2} {-}v_{ 2 6}$$

13 $${\text{PEP}}/{\text{PYR}} \quad 0\, = \,v_{ 2 6} \, + \,v_{ 3 3} {-}v_{ 2 7} {-}v_{ 3 1}$$

14 $$3 {\text{PG}} \quad 0\, = \,v_{ 3 1} {-}v_{ 3 2} {-}v_{ 3 9}$$

15 $${\text{SER}}_{\text{INTR}} \quad 0\, = \,v_{ 3 2} \, + \,v_{ 3 4} \, + \,v_{ 3 6} {-}v_{ 3 3} {-}v_{ 3 5}$$

16 $${\text{GLY}}_{\text{INTR}} \quad 0\, = \,v_{ 3 7} {-}v_{ 3 6} {-}v_{ 3 8} {-}v_{ 5 5}$$

17 $${\text{G3P}} \quad 0\, = \,v_{ 3 9} \, + \,v_{ 4 3} {-}v_{ 40} {-}v_{ 4 1} {-}v_{ 4 2} {-}v_{ 4 4} {-}v_{ 4 5}$$

18 $${\text{DHAP}}\quad 0\, = \,v_{ 4 1} {-}v_{ 4 4}$$

19 $${\text{F6P}} \quad 0\, = \,v_{ 4 4} {-}v_{ 4 3} {-}v_{ 4 5} {-}v_{ 4 6} {-}v_{ 4 7}$$

20 $${\text{S7P}} \quad 0\, = \,v_{ 4 3} {-}v_{ 4 2}$$

21 $${\text{E4P}} \quad 0\, = \,v_{ 4 5} {-}v_{ 4 3} {-}v_{ 5 3}$$

22 $${\text{G6P}} \quad 0\, = \,v_{ 4 7} {-}v_{ 4 8} {-}v_{ 50}$$

23 $${\text{Ru5P}} \quad 0\, = \,v_{ 50} \, + \,v_{ 5 2} {-}v_{ 5 1} {-}v_{ 6 3} {-}v_{ 6 4}$$

24 $${\text{Xu5P}} \quad 0\, = \,v_{ 4 2} \, + \,v_{ 4 5} {-}v_{ 5 2}$$

25 $${\text{R5P}} \quad 0\, = \,v_{ 4 2} \, + \,v_{ 5 1} {-}v_{ 5 4} {-}v_{ 5 5}$$

26 $${\text{IMP}} \quad 0\, = \,v_{ 5 5} {-}v_{ 5 8} {-}v_{ 5 9}$$

27 $${\text{GTP}}\quad 0\, = \,v_{ 5 8} \, + \,v_{ 6 1} {-}v_{ 60} {-}v_{ 6 3}$$

28 $${\text{DRL}} \quad 0\, = \,v_{ 6 3} {-}v_{ 6 4}$$

29 $${\text{RF}}\quad 0\, = \,v_{ 6 4} {-}v_{ 6 5}$$

**Table 1:** Estimation of the de novo demand for anabolic precursors during growth of *A.* *gossypii* on complex medium and rapeseed oil

Anabolic precursor	Total demand (µmol g_CDW_^−1^)		Uptake (%)^a^	De novo biosynthesis (%)	Resulting de novo demand (µmol g_CDW_^−1^)
G6P	604.8		12.5 ± 0.4	87.5 ± 0.4	529.2 ± 2.7
F6P^b^	821.0		0.0	100.0	821.0
R5P^b^	329.9		94.8	5.2	17.3
E4P	238.8		98.0 ± 2.9	2.0 ± 3.4	4.8 ± 7.2
G3P^b^	240.4		0.0	100.0	240.4
3PG	707.4		94.8 ± 5.1	5.2 ± 5.0	37.1 ± 36.0
PEP	449.6		97.9 ± 2.9	2.1 ± 2.7	9.6 ± 14.3
PYR	1783.1		96.0 ± 1.0	4.0 ± 1.0	71.5 ± 14.9
AcCoA	6572.7		n.d.	n.d.	n.d.
OAA	1124.5		88.5 ± 1.9	11.5 ± 1.9	129.8 ± 20.5
AKG	800.4		60.8 ± 1.5	39.2 ± 1.5	313.7 ± 10.7
NADPH	10,659.9		93.3 ± 0.1	6.7 ± 0.2	721.1 ± 62.3

First, the total precursor demand (total demand) was taken from literature [33] and adjusted for growth on vegetable oil based on [24]. The total demand for each anabolic precursor was covered by two routes: (i) uptake of external building blocks from complex ingredients, which biosynthetically originate from the respective precursor (e.g. alanine, valine, etc. from pyruvate) and (ii) de-novo synthesis of the building blocks from vegetable oil. Correlation of the total demand values with experimental summed fractional labeling (SFL_corr_) data from combined results of parallel ^13^C isotope studies with [^13^C_2_] glycine, [^13^C] formate, [^13^C_5_] glutamate, and [U^13^C] yeast extract (Additional file 1: Tables S2, S3) yielded the measured percentage of (i) the anabolic precursor that could be neglected due to the uptake of advanced metabolites (e.g. amino acids, nucleotides) and (ii) the resulting percentage of de novo precursor demand for growth on complex medium and rapeseed oil, which was then converted into the resulting de novo demand. The full length bar indicates the individual contributions visually: the purple fraction depicts the percentage covered from complex ingredients, while the grey fraction depicts the resulting de novo biosynthetic fraction of the precursor. The de novo demand for acetyl CoA was not specified, which is explained in more detail in Additional file 1: Table S5. 3PG, 3-phosphoglycerate; AcCoA, acetyl-CoA; AKG, α-ketoglutarate; E4P, erythrose 4-phosphate; F6P, fructose 6-phosphate; G3P, glyceraldehyde 3-phosphate; G6P, glucose 6-phosphate; OAA, oxaloacetate; PEP, phosphoenolpyruvate; PYR, pyruvate, R5P, ribose 5-phosphate. Contribution of nutrient uptake from the medium and de novo synthesis of precursors for *A.* *gossypii*

^a^Percentage negligible due to uptake of advanced building blocks, i.e. amino acids or nucleotides, from the medium. In the case of NADPH, the negligible fraction stems from the reduced de novo synthesis and thus, reduced NADPH demand of e.g. amino acids that are readily taken up from the medium

^b^The demand for the precursor is assumed. Therefore, no standard deviation could be calculated for the according values

The stoichiometric factor for *v*_1_ in Eq. (1) is derived from the assumption that one molecule oil is composed of one molecule glycerol and three fatty acid chains with a chain length of 17.3 carbon atoms each. Thus, all three chains would contribute 52 carbon atoms, which are degraded to 26 molecules acetyl-CoA. The rank of the stoichiometric matrix, formulated for the Eqs. (1) to (29) equaled 29. This indicated that all 29 metabolite balances were linearly independent. Since the complete network comprised a total of 65 fluxes, additional 36 pieces of information were required in addition to the stoichiometric balances. Anabolic fluxes based on the biomass composition of *A.* *gossypii* (Additional file 1: Table S4), which were correlated with the amount of precursor taken up by the cell versus the amount of de novo biosynthesis (i.e. ^13^C labeling information, Additional file 1: Tables S4, S5), delivered another 28 measured fluxes (*v*_3_, *v*_15_, *v*_16_, *v*_17_, *v*_18_; *v*_19_, *v*_22_, *v*_23_, *v*_24_, *v*_25_, *v*_27_, *v*_28_, *v*_29_, *v*_30_, *v*_35_, *v*_38_, *v*_40_, *v*_46_, *v*_48_; *v*_49_, *v*_53_, *v*_54_, *v*_56_, *v*_57_, *v*_59_, *v*_60_, *v*_61_, *v*_62_). The remaining 8 pieces of information were obtained through the measurement of extracellular fluxes, i.e. substrate uptake rates (*v*_1_, *v*_14_, *v*_37_) and riboflavin secretion rate (*v*_65_), and the measurement of intracellular fluxes derived from additional ^13^C labeling information (*v*_12_, *v*_33_, *v*_34_, *v*_36_) (Additional file 1: Tables S2, S3). Anabolic fluxes were calculated by multiplying the anabolic demand with the biomass yield, Y_X/Oil_, and the uptake rate of the main carbon source, q_S,Oil_ (Table 2). In total, 65 pieces of information were obtained, which rendered a fully determined metabolic network for flux calculations.

**Table 2:** Growth and production kinetics of *A.* *gossypii* B2 grown on complex medium with rapeseed oil as main carbon source

Rates		
µ_max_ (h^−1^)		0.07 ± 0.00
q_S,Oil_ (mmol g^−1^ h^−1^)		0.43 ± 0.06
q_S,Glu_ (mmol g^−1^ h^−1^)		0.14 ± 0.02
q_S,Gly_ (mmol g^−1^ h^−1^)		0.08 ± 0.02
q_P,max_ (µmol g^−1^ h^−1^)		16.96 ± 1.96
Q_P,max_ (µmol L^−1^ h^−1^)		352.04 ± 52.20
Yields		
Y_X/Oil_ (g mol^−1^)		186.1 ± 21.4
Y_X/Glu_ (g mmol^−1^)		0.56 ± 0.08
Y_X/Gly_ (g mmol^−1^)		0.98 ± 0.21
Y_RF/Oil_ (mmol mol^−1^)		281.6 ± 36.1
Y_RF/Glu_ (mmol mol^−1^)		886.4 ± 131.4
Y_RF/Gly_ (mmol mol^−1^)		152.4 ± 14.4

Rates (q, Q) and yield coefficient (Y) represent mean values from three independent replicates. RF, riboflavin; Glu, glutamate; Gly, glycine; P, product; S, substrate; X, biomass

### Estimation of metabolic fluxes during riboflavin biosynthesis

During riboflavin biosynthesis on vegetable oil (Additional file 1: Fig. S4), the fluxes to be determined were calculated and expressed as fluxes of single carbon atoms. This facilitated flux calculations, because every carbon atom of riboflavin had a unique labeling fingerprint obtained from the four tracer studies with [^13^C_2_] glycine, [^13^C] formate, [^13^C_5_] glutamate, and [U^13^C] yeast extract. Since a single carbon atom of riboflavin could originate from a number of different medium components as well as intracellular metabolites, single carbon atom balancing met the challenges of complex data handling the best. Therefore, for every carbon atom in riboflavin, the metabolic precursor carbon atom and most likely potential other donor atoms were considered (Additional file 1: Figs. S6–S8) and the flux (*v*) into the respective carbon atom was set to 1 and formulated as follows:

30 $$v_{i} = \mathop \sum \limits_{j = 1}^{5} v_{i,j} = 1$$

31 $$v_{i,j} = x_{i,j}$$

32 $$v_{i,Gly} = x_{i,Gly}$$

33 $$v_{i,For} = x_{i,For}$$

34 $$v_{i,Glu} = x_{i,Glu}$$

35 $$v_{i,YE} = x_{i,YE}$$

36 $$v_{i,Oil} = 1 - v_{i,Gly} - v_{i,For} - v_{i,Glu} - v_{i,YE}$$

with

$$v_{i} \quad {\text{Carbon atom of riboflavin with number}}\,i$$

$$\begin{aligned} j\quad {\text{Index specifying the}}\; ^{ 1 3} {\text{C tracer used }} (& 1{:} {\text{ glycine}};\; 2{:}{\text{ formate}}; \\ & \; {3}{:} {\text{ glutamate}};\; {4}{:}{\text{ yeast extract }}\left( {\text{YE}}\right); \; {5}{:}{\text{ oil}}) \\ \end{aligned}$$

$$x_{i} \quad {\text{Specific}}\; ^{ 1 3} {\text{C enrichment at carbon atom}}\; i \;{\text{of riboflavin from a certain}}\; ^{ 1 3} {\text{C tracer}}\; j$$

Note that oil was not used as ^13^C tracer, but the enrichment (*x*_*Oil*_) and the resulting flux (*v*_*Oil*_) was a result of the combined enrichment of all ^13^C tracers used (formate, glycine, glutamate, and yeast extract). The Eqs. (30) to (36) could then be applied in detail to every carbon atom in riboflavin (Additional file 1).

Based on the carbon atom fluxes, the calculation of fluxes between metabolites (designated by a capital *V*), i.e. a sum of several carbon atoms, could then be formulated for the three major structural units that comprise the vitamin (Additional file 1: Figs. S4, S7–S9). Note that riboflavin contains 17 carbon atoms in total, the xylene ring eight carbon atoms, the ribityl side chain five carbon atoms, and the pyrimidine ring four carbon atoms:

37 $$V_{55} = V_{RF} = \mathop \sum \limits_{i = 1}^{17} v_{i} = \mathop \sum \limits_{i = 1}^{17} \mathop \sum \limits_{j = 1}^{5} v_{i,j} = 17$$

38 $$V_{40} = V_{Xylene} = \mathop \sum \limits_{i = 5a}^{9a} v_{i} = \mathop \sum \limits_{i = 5a}^{9a} \mathop \sum \limits_{j = 1}^{5} v_{i,j} = 8$$

39 $$V_{Ribityl} = \mathop \sum \limits_{i = 1'}^{5'} v_{i} = \mathop \sum \limits_{i = 1'}^{5'} \mathop \sum \limits_{j = 1}^{5} v_{i,j} = 5$$

40 $$V_{Pyrimidine} = v_{2} + v_{4} + v_{4a} + v_{10a} = 4$$

41 $$V_{54} = V_{Ribityl} + V_{Pyrimidine}$$

Subsequent calculations and metabolite balances leading to the complete picture of carbon fluxes during the riboflavin biosynthetic phase, are given in the Additional file 1.

## Results

### *A. gossypii* B2 accumulates elevated amounts of riboflavin in a two-stage process

**Fig. 2:**
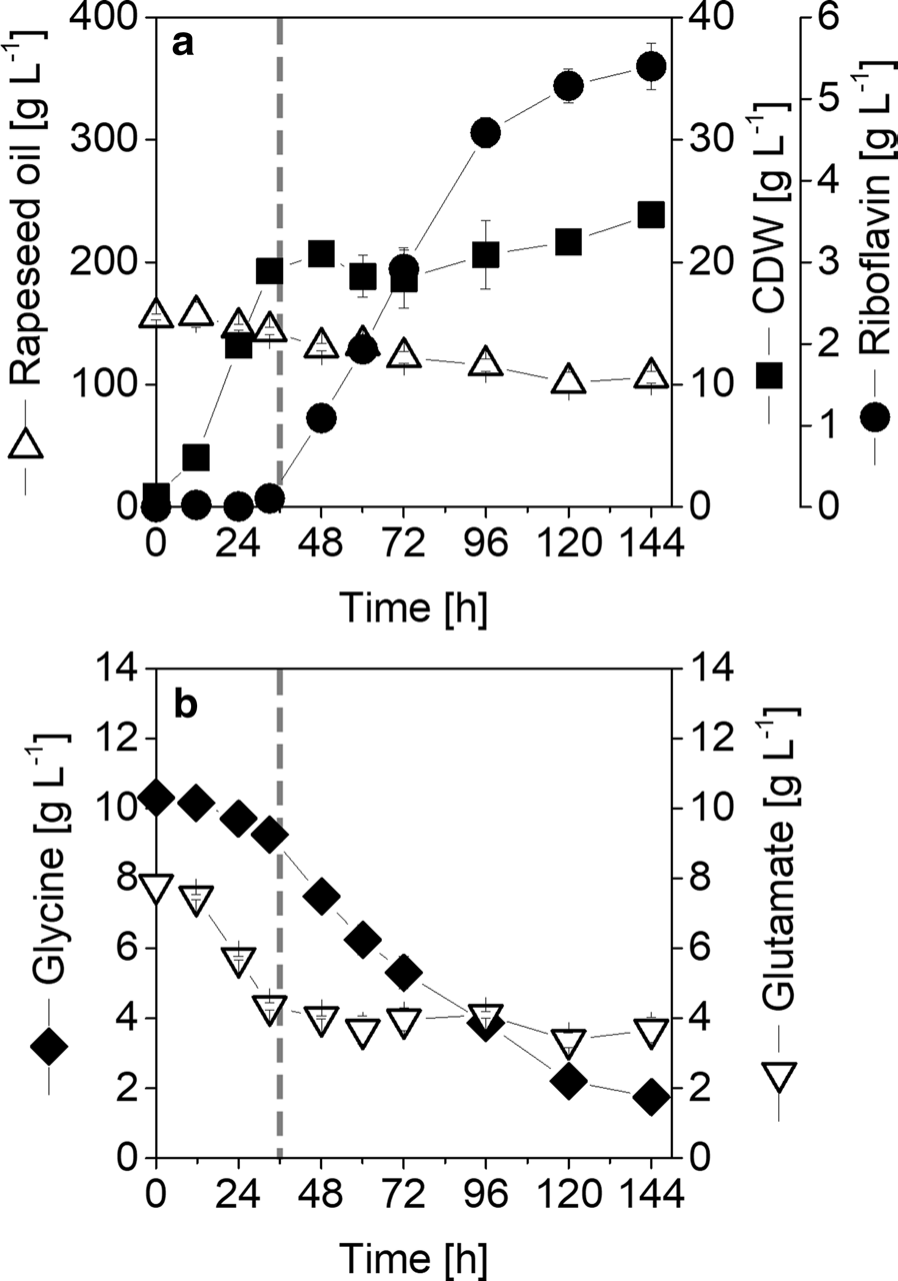
Growth and riboflavin production of *A.* *gossypii* B2 on complex medium with rapeseed oil. The data represent mean values and the deviation for three shake flasks, sacrificed per data point. CDW, cell dry weight

*Ashbya gossypii* B2 was cultivated on a typical production medium containing yeast extract, glycine, glutamate as well as formate with rapeseed oil as main carbon source. The two-phase cultivation profile was characterized by an initial growth phase, which lasted 36 h, and a subsequent riboflavin production phase (Fig. 2, Table 2). This process was now studied by isotope experiments, using fully ^13^C-labeled yeast extract as a replacement for the naturally labeled yeast extract. Since fully ^13^C-labeled yeast extract was not available, it was custom-synthesized by processing a yeast strain, commercially used for the production of yeast extract on ^13^C-labeled carbon source. Subsequently, the isotope purity as well as influence on productivity of the custom-synthesized yeast extract was inspected. For that, it was hydrolyzed and the resulting amino acids analyzed with GC/MS. The respective fully ^13^C-labeled mass isotopomer could be observed for all measured amino acids with high abundance (Fig. 1c). The obtained summed fractional labeling (SFL) was in the range of 98–99% for all amino acids (Additional file 1: Table S1). This isotope purity was optimal as it matched the ^13^C purity of commercially available ^13^C tracer substances, such as sugars and amino acids. Regarding its impact on the production performance of *A.* *gossypii* B2, riboflavin titers, observed on the custom-synthesized yeast extract did not significantly differ from the ones on naturally labeled yeast extract.

### Yeast extract is the primary carbon source during growth

**Fig. 3:**
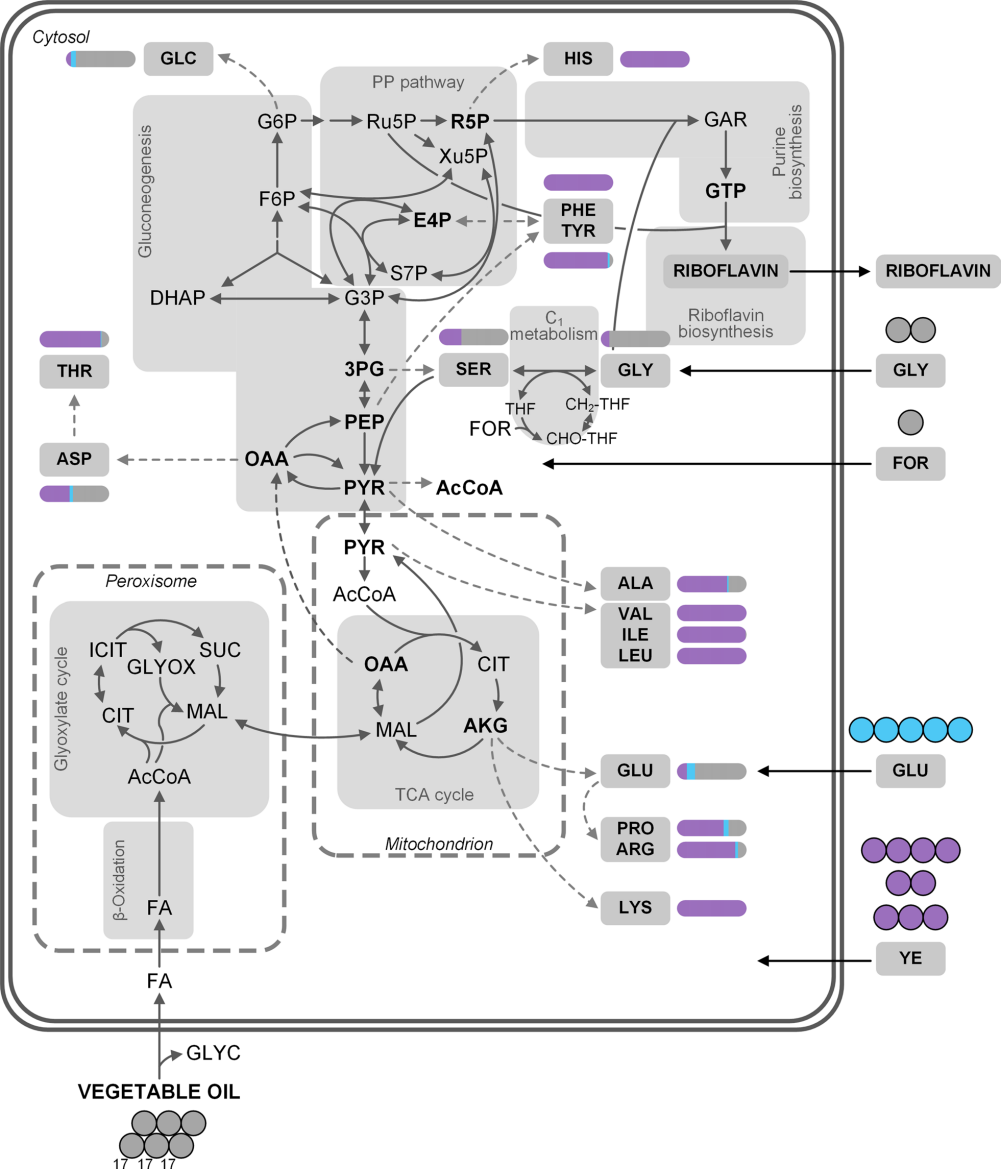
Relative contribution of ^13^C tracers to amino acids from hydrolyzed cell protein and glycogen of *A.* *gossypii* B2 grown on complex medium with rapeseed oil. The ^13^C labeling patterns of the amino acids were obtained at the end of the growth phase after 36 h. Respective contribution of [U^13^C] yeast extract (purple), [^13^C_5_] glutamate (light blue), and other media components (grey) are depicted by the bar next to the respective amino acid. The full length bar represents 100%. Data represent summed fractional labelings (SFL_corr_), corrected for natural labeling and dilution effects through unlabeled pre-culture medium. Data were obtained from three independent replicates. Note that the conversion of citrate to isocitrate via aconitase most likely does not occur in the peroxisome [68]. 3PG, 3-phosphoplycerate; CH_2_-THF, 5,10-methylenetetrahydrofolate; AcCoA, acetyl-CoA; AKG, α-ketoglutarate; ALA, alanine; ARG, arginine; ASP, aspartate; CHO-THF, 10-formyltetrahydrofolate; CIT, citrate; DHAP, dihydroxyacetone phosphate; E4P, erythrose 4-phosphate; F6P, fructose 6-phosphate; FA, fatty acids; FOR, formate; G3P, glyceraldehyde 3-phosphate; G6P, glucose 6-phosphate; GAR, glycineamide ribonucleotide; GLU, glutamate; GLY, glycine; GLYC, glycerol; GLYOX, glyoxylate; GTP, guanosine triphosphate; HIS, histidine; ICIT, isocitrate; ILE, isoleucine; LEU, leucine; MAL, malate; OAA, oxaloacetate; PEP, phosphoenolpyruvate; PHE, phenylalanine; PP pathway, pentose phosphate pathway; PRO, proline; PYR, pyruvate; R5P, ribose 5-phosphate; Ru5P, ribulose 5-phosphate; S7P, sedoheptulose 7-phosphate; SER, serine; TCA cycle, tricarboxylic acid cycle; THF, tetrahydrofolate; THR, threonine; TYR, tyrosine; VAL, valine; Xu5P, xylulose 5-phosphate; YE, yeast extract

**Fig. 4:**
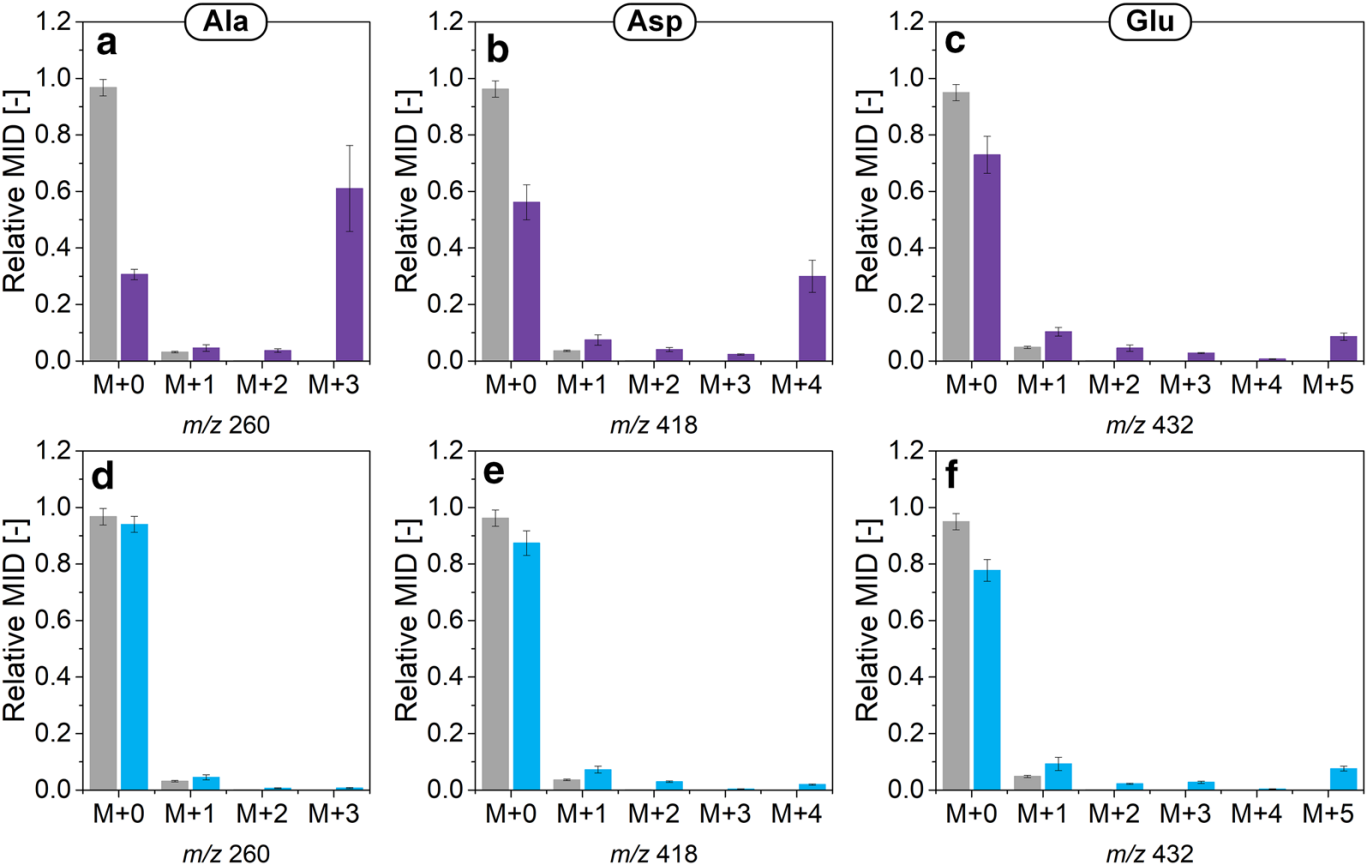
Relative mass isotopomer distributions (MIDs) of proteinogenic alanine (**a**, **d**), aspartate (**b**, **e**), and glutamate (**c**, **f**) from *A.* *gossypii* B2 grown on complex medium with rapeseed oil as main carbon source. MIDs are presented for medium with naturally labeled substrates (grey), [U^13^C] yeast extract (purple), and [^13^C_5_] glutamate (light blue). Data were obtained from three individual replicates

In a first experiment, the formation of cell building blocks from yeast extract was investigated. When fully ^13^C-labeled yeast extract was added to the medium and substituted the naturally labeled yeast extract, amino acids from biomass harvested at the end of the exponential growth phase (36 h), were largely ^13^C-labeled (Fig. 3, Additional file 1: Table S3), which indicated a strong uptake of yeast extract-based components and subsequent incorporation into the cell protein (Fig. 3, Additional file 1: Table S3). A few selected amino acids, however, exhibited less ^13^C incorporation: alanine, aspartate, and glutamate (SFL_corr_ of 72 ± 4%, 43 ± 3%, and 14 ± 1%, respectively) (Figs. 3, 4), pointing to  a different metabolic origin for those amino acids. To analyze this process in more detail, an additional study with [^13^C_5_] glutamate as the only labeled medium ingredient was carried out. Still, intracellular alanine, aspartate, and glutamate were only slightly ^13^C-enriched (Figs. 3, 4), suggesting the oil, the only remaining nutrient, as the main donor for these three amino acids (see Additional file 1). Serine and glycine also showed comparatively low ^13^C enrichments with SFL_corr_ of 32 ± 1% and 12 ± 1%, respectively (Fig. 3, Additional file 1: Table S3). This matched the previous observation that, in fact, the supplemented glycine was the major contributor to the intracellular serine and glycine pools [16].

In addition, the ^13^C-labeling incorporation of glycogen-derived cellular glucose was inspected. The replacement of yeast extract or glutamate with their fully ^13^C-labeled isotopomers resulted both in a SFL_corr_ of about 6 ± 0% for glucose, respectively (Fig. 3, Additional file 1: Table S3). The single labeled mass isotopomer (M + 1) was threefold increased, as compared to the naturally labeled compound (Additional file 1: Table S3). Accordingly, the majority of cellular glucose and glycogen originated from vegetable oil as well (Fig. 3). Taken together this indicated, at least on an additive level, that the tricarboxylic acid (TCA) cycle was active (glutamate conversion) as also were the glyoxylate shunt and the gluconeogenic route (oil conversion into glycogen).

### Metabolic fluxes into anabolism during growth on complex medium

The data gained in the ^13^C tracer studies mentioned above, gave a detailed overview of the origin of growth associated building blocks. The results in the previous chapters demonstrated that a large fraction of cellular protein and glycogen was covered by mere uptake of amino acids and yeast extract supplemented in the medium, while the impact of extracellular glutamate was reduced in comparison (Fig. 3, Additional file 1: Tables S2, S3). These data were now used to estimate the remaining fluxes from carbon core pathway intermediates into biomass precursors and building blocks, i.e. providing a realistic picture on the metabolic fluxes into anabolism for the given complex nutrient conditions. Because most of the amino acids were either fully or partially taken up from the culture medium, the precursor demand to form these molecules de novo was tremendously decreased. As example, the demand for pyruvate, expected for growth on minimal medium (1783.1 µmol g_CDW_^−1^) was only 71.5 ± 14.9 µmol g_CDW_^−1^ for growth on the complex medium, when ^13^C labeling data were taken into account. Thus, the experimental de novo precursor demand for growth on complex medium differed greatly from values, expected for full de novo synthesis of biomass (Table 1). This finding underlined the importance of taking the ^13^C labeling data into consideration as well as the impact of the medium to support intracellular pools. The estimated precursor demands could be taken as a representative picture of the studied process and provided consistent data for further flux calculations.

### Growth phase: *A.* *gossypii* reveals high flux through β-oxidation and TCA cycle

**Fig. 5:**
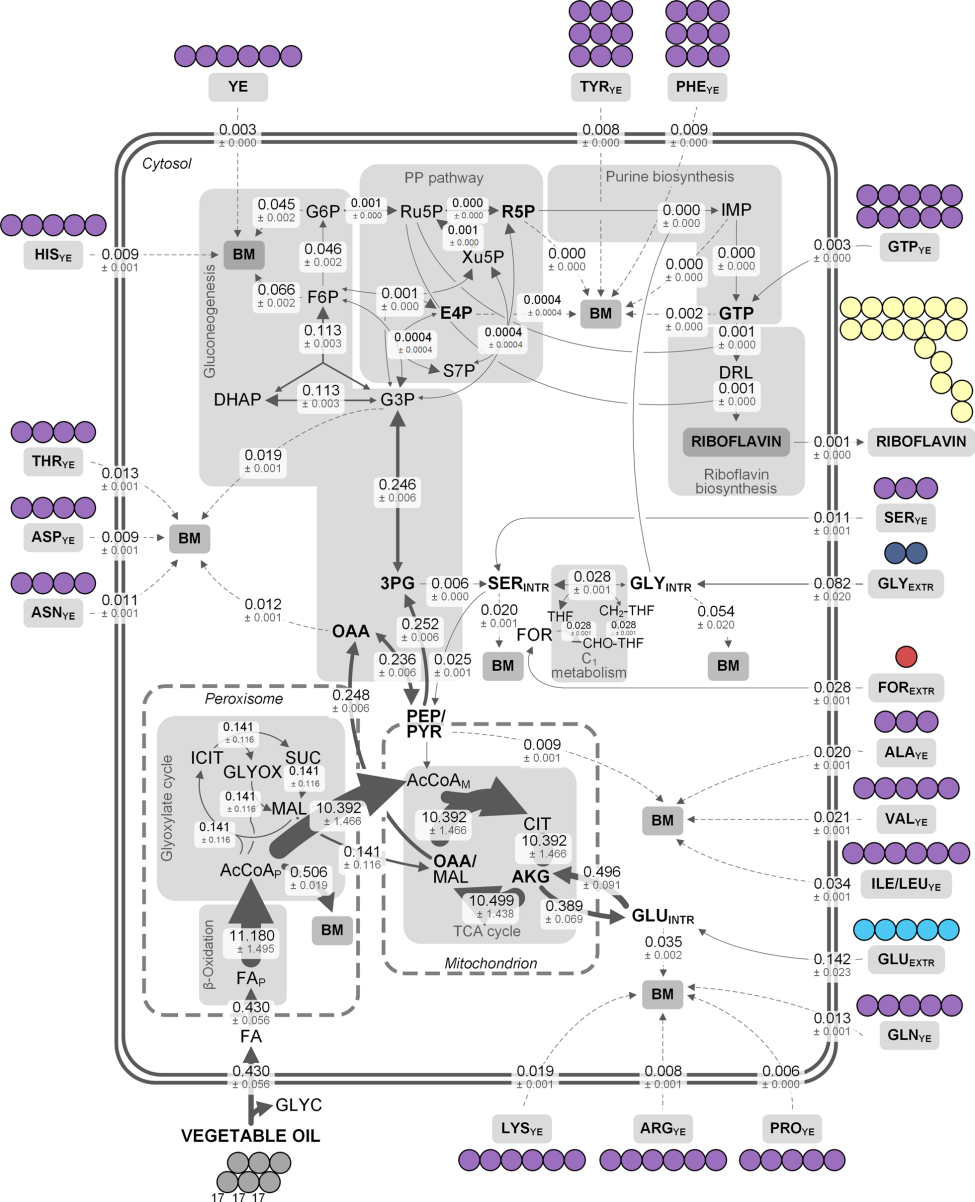
Carbon fluxes during growth of *A.* *gossypii* B2 on rapeseed oil and complex medium. Fluxes are given in mmol g_CDW_^−1^ h^−1^ and are normalized to the substrate uptake rate (0.43 ± 0.06 mmol g^−1^ h^−1^). Flux calculations were derived from parallel ^13^C tracer studies with [^13^C_2_] glycine, [^13^C] formate, [^13^C_5_] glutamate, and [U^13^C] yeast extract (Additional file 1: Tables S2, S3). The arrow thickness is proportional to the corresponding flux. The direction of net fluxes is indicated by size of arrow head. Dashed arrows represent fluxes into biomass. Reactions at the OAA/MAL and PEP/PYR node could not be resolved by this approach and represent lumped fluxes. The rate of the pyruvate dehydrogenase could not be determined. In order to simplify the figure, only GTP uptake is depicted as immediate riboflavin precursor. Other nucleotides as well as cysteine and methionine, which are also taken up from the medium, are not depicted. Note that the conversion of citrate to isocitrate via aconitase most likely does not occur in the peroxisome [68]. 3PG, 3-phosphoplycerate; AcCoA_P/M_, peroxisomal/mitochondrial acetyl-CoA; AKG, α-ketoglutarate; ALA, alanine; ARG, arginine; ASN, asparagine; ASP, aspartate; BM, biomass; CH_2_-THF, 5,10-methylenetetrahydrofolate; CHO-THF, 10-formyltetrahydrofolate; CIT, citrate; DHAP, dihydroxyacetone phosphate; E4P, erythrose 4-phosphate; F6P, fructose 6-phosphate; FA, fatty acids (here three FA with an average chain length of 17.3 carbon atoms); FOR, formate; G3P, glyceraldehyde 3-phosphate; G6P, glucose 6-phosphate; GAR, glycineamide ribonucleotide; GLN, glutamine; GLU, glutamate; GLY, glycine; GLYC, glycerol; GLYOX, glyoxylate; GTP, guanosine triphosphate; IMP, inosine monophosphate; MAL, malate; OAA, oxaloacetate; PEP, phosphoenolpyruvate; PYR, pyruvate; R5P, ribose 5-phosphate; Ru5P, ribulose 5-phosphate; S7P, sedoheptulose 7-phosphate; SER, serine; THF, tetrahydrofolate; Xu5P, xylulose 5-phosphate; YE, yeast extract

Measured ^13^C labeling data from the cell protein, the determined anabolic fluxes from medium ingredients and from pathway precursors, and measured extracellular fluxes provided a rich data set for the flux calculation. The network was fully determined so that the fluxes could be obtained through the established set of mass balances (see above and Additional file 1). Growth of *A.* *gossypii* on vegetable oil and complex medium resulted in a strong carbon flux through the ß-oxidation pathway as well as the TCA cycle (Fig. 5). Vegetable oil was cleaved into three fatty acids (average chain length of 17.3 carbon atoms, denoted as FA in Fig. 5) [24] and glycerol in the extracellular space [38]. Fatty acids were then taken up by the cell (0.430 ± 0.056 mmol g^−1^h^−1^) and oxidized to acetyl-CoA through the ß-oxidation pathway, located in the peroxisome [39, 40]. The carbon flux from acetyl-CoA into biomass (0.506 ± 0.019 mmol g^−1^h^−1^) assumes that the intracellular storage lipids have to be synthesized de novo, which is, however, unlikely as described below. A small fraction of acetyl-CoA, i.e. 0.141 ± 0.116 mmol g^−1^h^−1^, was then assimilated through the glyoxylate shunt, yielding an overall carbon flux into malate of 0.141 ± 0.116 mmol g^−1^h^−1^. The majority of acetyl-CoA, however, was channeled into the TCA cycle, where it was decarboxylated and finally contributed to cellular respiration. The highly reduced fatty acids are oxidized in order to become accessible for the cell. The strong decarboxylation of acetyl-CoA in the TCA cycle is balanced by oxygen consumption during cellular respiration. These facts match the typically observed RQ of < 1 for growth on lipids [24], which is characterized by high oxygen consumption and low carbon dioxide formation.

The acetyl-CoA that was channeled through the TCA cycle plus the oxaloacetate from the glyoxylate shunt together formed citrate, which was then decarboxylated to α-ketoglutarate (Fig. 5). The large flux from α-ketoglutarate to intracellular glutamate (0.389 ± 0.069 mmol g^−1^h^−1^) matched the observed ^13^C enrichment of glutamate (combined SFL_corr_ of 26.5%), which was largely naturally labeled, given the strong uptake of extracellular glutamate during growth (0.142 ± 0.023 mmol g^−1^h^−1^). The intracellular glutamate pool fed into biomass: while Fig. 5 only shows lumped reactions, Additional file 1: Fig.S3a displays a more detailed picture. Carbon fluxes into the single proteinogenic amino acids illustrate the strong regulation of their biosynthesis: intracellular glutamate only supplied small fractions of the respective amino acids, while most of their demand was covered through uptake from the medium (Additional file 1: Fig. S3a). The strong backflux from glutamate to α-ketoglutarate resulted in an α-ketoglutarate-synthesizing netflux (0.107 mmol g^−1^ h^−1^) (Fig. 5). Reactions involving the OAA/malate pool as well as the PEP/pyruvate pool could not be resolved by this approach and were calculated as lumped reactions, i.e. a net flux from oxaloacetate to the combined PEP/pyruvate pool (0.236 ± 0.006 mmol g^−1^ h^−1^). Oxaloacetate is an important anabolic precursor, contributing to the synthesis of various amino acids as well as pyrimidine biosynthesis (Additional file 1: Table S4). The detailed distribution of carbon fluxes into biomass starting from the precursor oxaloacetate is presented in Additional file 1: Fig. S3b. Intracellular aspartate is derived from extracellular aspartate and to similar extent from oxaloacetate. However, there was only a small flux of 0.003 ± 0.000 mmol g^−1^ h^−1^ from intracellular aspartate to asparagine and threonine, on the other hand, indicating strong uptake of these two amino acids from the medium (fluxes of 0.011 ± 0.001 mmol g^−1^h^−1^ and 0.013 ± 0.001 mmol g^−1^ h^−1^, respectively) (Additional file 1: Fig. S3b).

### Growth phase: Gluconeogenesis and pentose phosphate pathway fluxes are low

The carbon fluxes through the upper metabolism, i.e. gluconeogenesis and PP pathway were comparatively low. This pattern can be attributed to the high uptake of amino acids from the culture medium and the resulting low de novo precursor demand. Here, the precursor demand for ribulose 5-phosphate, ribose 5-phosphate, and erythrose 4-phosphate was almost completely covered by the reactions of the non-oxidative part of the PP pathway (Fig. 5). A small carbon flux of 0.001 ± 0.000 mmol g^−1^ h^−1^ was assigned to the oxidative branch. The flux distribution presented shows that the glyoxylate shunt plays an important role in carbon assimilation during growth on vegetable oil, which is then channeled into gluconeogenesis. The decreased carbon flux through the upper metabolism, i.e. gluconeogenesis and PP pathway, reflects the strong regulation of amino acid de novo biosynthesis due to the ample supply of amino acids as well as nucleotides in the culture supernatant [41]. While pioneering ^13^C flux studies used ethanol or glucose as carbon sources on semi-defined media [25, 42], this is the first report of a carbon flux distribution with *A.* *gossypii* on vegetable oil and complex medium during growth. The concerted action of the main carbon source vegetable oil and the complex substrate yeast extract regarding the formation of cell building blocks, illustrated by the flux map (Fig. 5), highlights the complexity of the given process.

### Differences in positional ^13^C enrichment indicate direct incorporation of complex building blocks into riboflavin

**Fig. 6:**
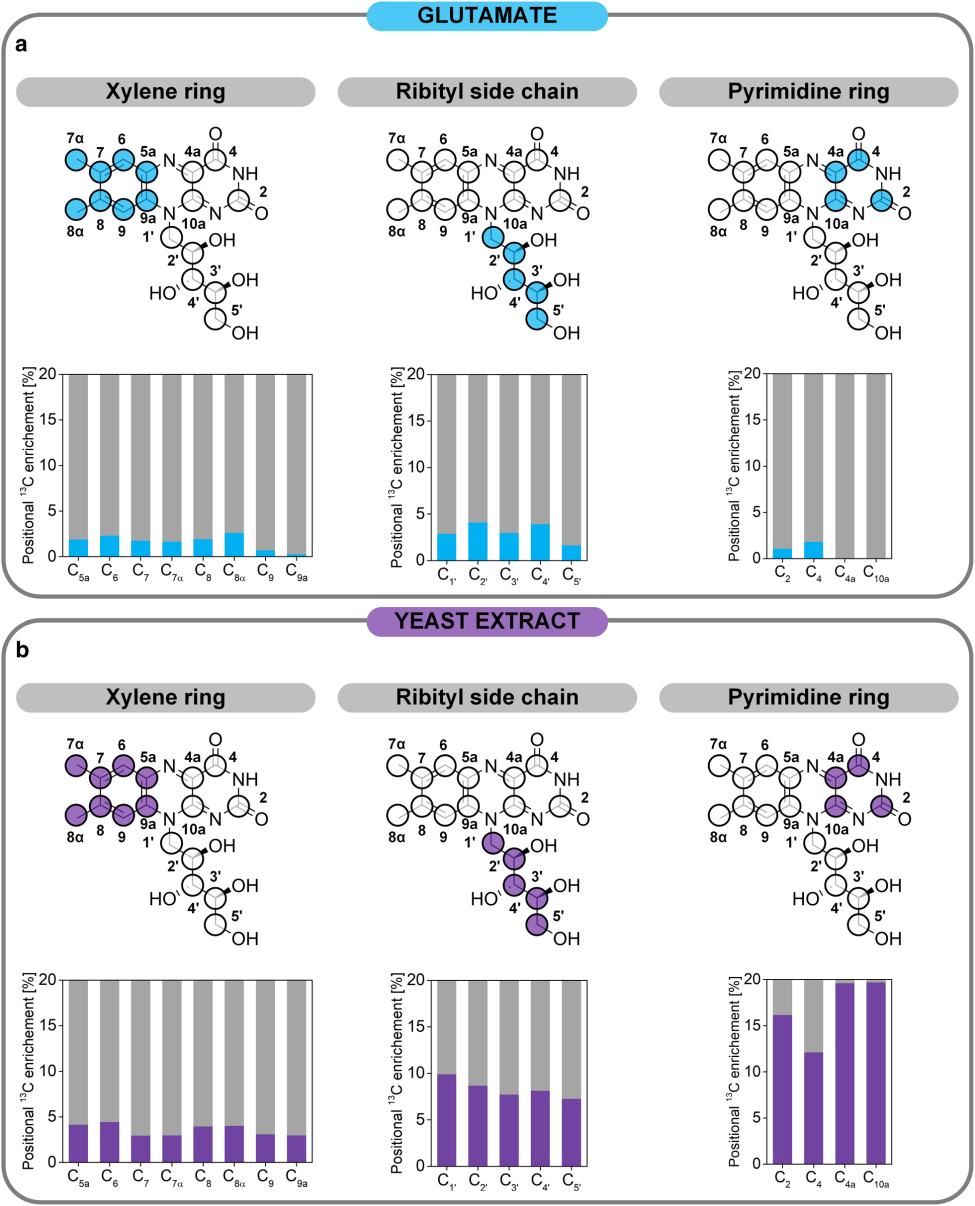
Experimental ^13^C enrichment of riboflavin produced on [^13^C_5_] glutamate (**a**) and fully ^13^C-labeled yeast extract (**b**) measured via ^13^C NMR. The initial naturally labeled medium was replaced by medium containing ^13^C-labeled yeast extract via centrifugation after 32 h of cultivation. The fully ^13^C-labeled glutamate replaced the naturally labeled glutamate in the medium at the beginning of a parallel experiment with otherwise naturally labeled medium compounds. The circles of the riboflavin molecules depict carbon atoms. Colored circles denote the part of the riboflavin molecule under investigation. The colors purple and light blue in the bar charts represent the percentage originating from the respective ^13^C-labeled precursor, while the grey bars indicate origin from a different medium ingredient. Data denote values from three independent experiments with a mean standard deviation of 5%

**Fig. 7:**
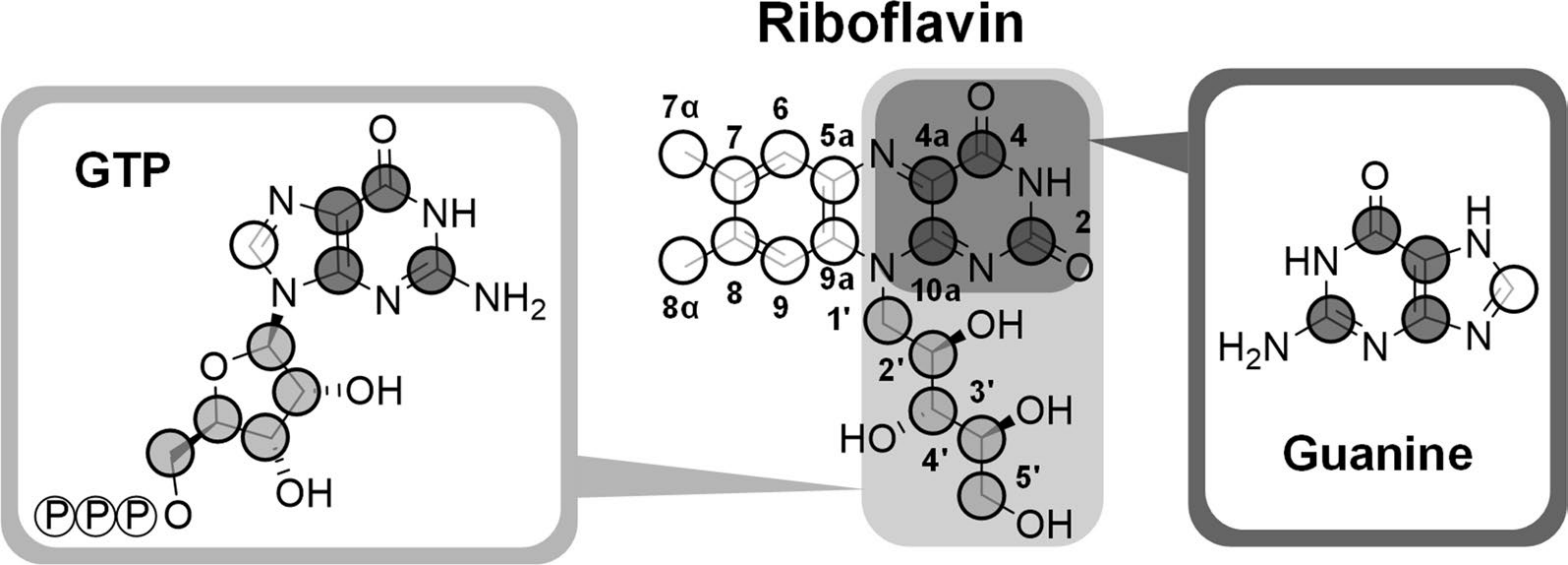
Structures of riboflavin, guanosine triphosphate (GTP), and guanine. GTP shares nine of its ten carbon atoms with riboflavin. Guanine shares all five carbon atoms with GTP and four of those with riboflavin. Only the xylene ring (white circles in riboflavin) is the structurally exclusive unit only found in the vitamin compared to the other two compounds. Grey circles denote carbon atoms that are shared between GTP, guanine, and riboflavin

Specific experiments with fully labeled yeast extract and positional ^13^C analysis of the riboflavin carbon backbone by NMR could distinguish whether ^13^C-labeled yeast extract-based compounds were incorporated into riboflavin in the early riboflavin production phase (the first 32 h of cultivation) or if the incorporation occurred after growth had ceased (32 h to 144 h) (Additional file 1). The NMR results revealed that during growth, ^13^C incorporation into riboflavin was below the detection limit and thus negligible (data not shown). Replacement of the naturally labeled medium with medium containing [U^13^C] yeast extract (32 h post inoculation) resulted in different degrees of ^13^C enrichment in every carbon atom of the vitamin (Table 3, Fig. 6b). Obviously, yeast extract-related compounds, present in the medium at the beginning of riboflavin accumulation were used for the formation of the product. The labeling results can be lumped into three structural subunits of the vitamin: ribityl side chain, xylene ring, and pyrimidine ring. On average 8.4 ± 0.2% of ^13^C labeling originating from ^13^C-labeled yeast extract could be detected in the ribityl side chain. Surprisingly, the xylene ring, which originates from the same intracellular precursor, the pentose 5-phosphate pool [20, 34, 43], exhibited 2.3-fold less ^13^C incorporation (3.6 ± 0.1%). The pyrimidine ring, containing the four remaining carbon atoms C_2_, C_4_, C_4a_, and C_10a_, was labeled the most with 16.2 ± 1.3%, 12.1 ± 1.0%, 19.6 ± 1.6%, and 19.7 ± 1.5%, respectively. The ^13^C labeling of C_4a_ and C_10a_ most likely originated from glycine and serine present in the yeast extract. Previous studies showed that serine is converted into glycine via the SHMT [16, 19], rendering a carbon-one unit, which is then also incorporated into the vitamin at carbon atom C_2_. There are two interesting details about the obtained labeling data: first, the heavy labeling of the C_4_ atom, which metabolically originates from carbon dioxide fixation in the purine biosynthesis and second, the difference in labeling between ribityl side chain and xylene ring. In a previous study investigating the carbon origin of the xylene ring using precursors such as [1,3-^13^C] or [2,3-^13^C] ribose, labeling between those two structural groups did not differ to such great extent [20, 43]. However, in that study only the amount of de novo synthesized riboflavin from the respective precursor was assessed. In riboflavin, the xylene ring is the riboflavin-exclusive structural unit that contains eight carbon atoms. All other carbon is derived from the immediate riboflavin precursor GTP (Fig. 7). Yeast extract is a complex nutrient, obtained from autolyzed yeast cells, which contains proteins to the largest extent, but also nucleotides or nucleobases from RNA or DNA and sugar compounds [41, 44]. The stronger ^13^C labeling in the ribityl side chain compared to the xylene ring as well as the heavy labeling of the C_4_ atom suggest that GTP or ATP were taken up from the yeast extract and incorporated into the vitamin. Thus, a maximum of 4 ± 0% ^13^C enrichment, i.e. the difference in labeling of the xylene ring and the ribityl side chain, could be attributed to nucleotides in the medium originating from yeast extract. Consequently, 8 ± 1% of ^13^C enrichment from ^13^C-labeled yeast extract at the C_4_ atom of riboflavin had to be attributed to a different origin. The ^13^C labeling could originate from ^13^CO_2_, which was produced during a decarboxylation reaction of a yeast extract compound. Considering the extent of ^13^C labeling (8%) the more likely explanation would be that guanine or adenine, also present in yeast extracts [41], was incorporated into riboflavin via the formation of GTP (Fig. 7).

**Table 3:** Relative ^13^C enrichment of all seventeen carbon atoms of riboflavin produced by *A.* *gossypii* B2 from different ^13^C-labeled tracer substrates

C-atom	Chemical shift (ppm)	Corrected relative enrichment (%)					
		Nat. lab. precursors	[^13^C] For		[^13^C_2_] Gly	[U^13^C] YE	[^13^C_5_] Glu
		0 h	0 h	48 h	0 h	32 h	0 h
2	155.5	0.0	3.7	11.6	0.0	16.2	1.1
4	159.9	0.0	0.0	0.0	0.0	12.1	1.8
4a	136.8	0.0	0.0	0.0	72.1	19.6	0.0
5a	134	0.0	0.0	0.0	0.0	4.2	1.9
6	130	0.0	0.0	0.0	0.0	4.5	2.3
7	137.1	0.0	0.0	0.0	0.0	3.0	1.8
7α	18.8	0.0	0.0	0.0	0.0	3.0	1.7
8	146	0.0	0.0	0.0	0.0	4.0	1.9
8α	20.8	0.0	0.0	0.0	0.0	4.0	2.6
9	117.4	0.0	0.0	0.0	0.0	3.1	0.7
9a	132.1	0.0	0.0	0.0	0.0	3.0	0.3
10a	150.8	0.0	0.0	0.0	71.6	19.7	0.0
1′	47.3	0.0	0.0	0.0	0.0	9.9	2.9
2′	68.8	0.0	0.0	0.0	0.0	8.7	4.1
3′	73.6	0.0	0.0	0.0	0.0	7.7	3.0
4′	72.8	0.0	0.0	0.0	0.0	8.1	3.9
5′	63.4	0.0	0.0	0.0	0.0	7.3	1.6

The labeling was analyzed by ^13^C NMR. The measurement data (uncorrected, Additional file 1: Table S6) were corrected values for naturally occurring isotopes and dilution through naturally labeled pre-culture medium. The time refers to the respective tracer addition. Data denote mean values of three independent replicates with a mean standard deviation of 8%

For, formate; Glu, glutamate; Gly, Glycine; Ser, serine; YE, yeast extract

### Riboflavin production phase: Vegetable oil is the main donor for the vitamin

**Fig. 8:**
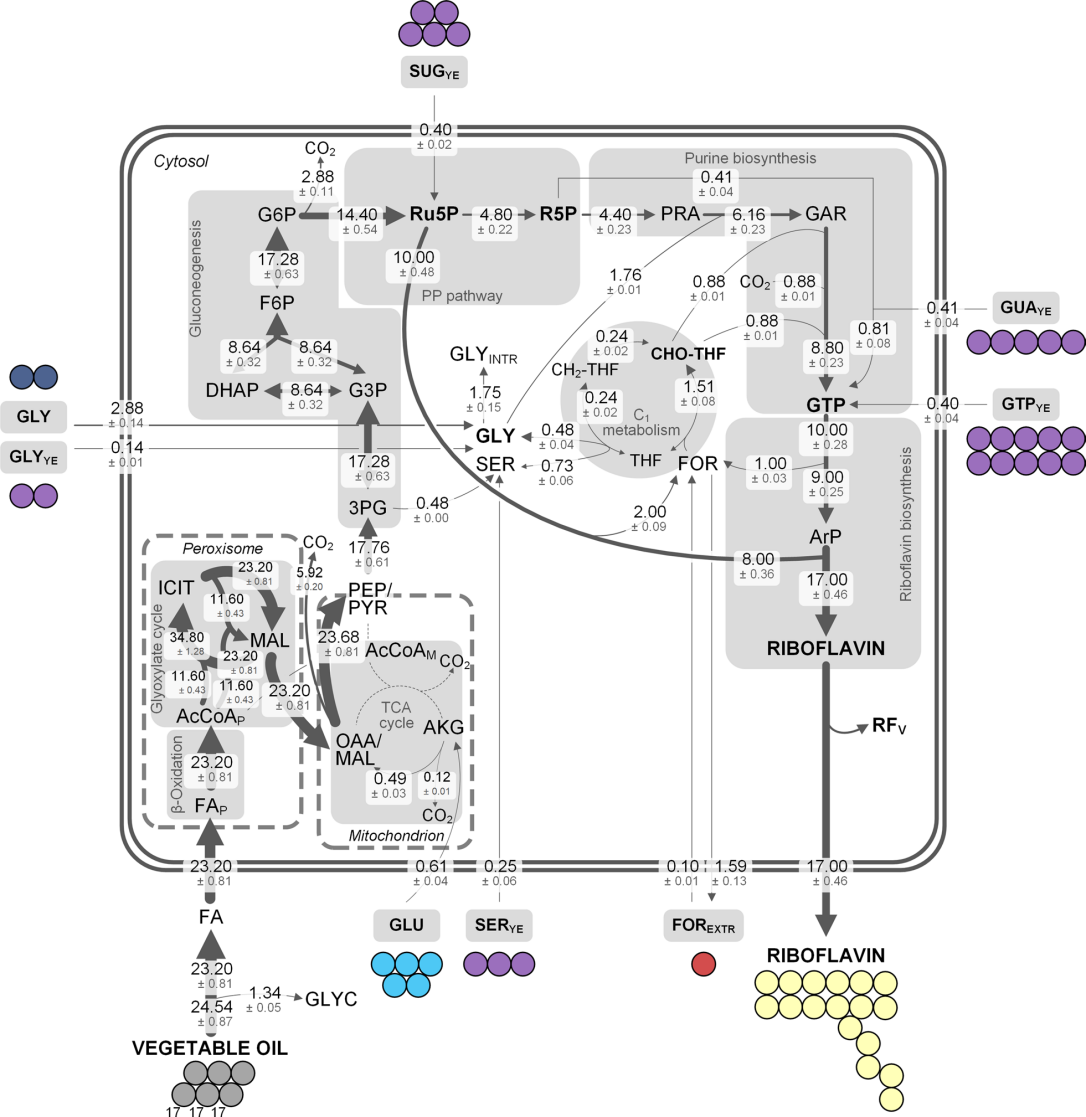
Carbon contribution of medium ingredients to riboflavin based on combined parallel ^13^C-labeled tracer studies using [^13^C_2_] glycine, [^13^C] formate, [^13^C_5_] glutamate, and [U^13^C] yeast extract. Riboflavin was synthesized by *A.* *gossypii* B2 grown on complex medium with rapeseed oil. Riboflavin was obtained at the end of the growth phase of riboflavin producing *A.* *gossypii* after 144 h. Data are derived from positional ^13^C enrichment obtained from ^13^C NMR measurements (Table 3), corrected for natural labeling and dilution effects through unlabeled pre-culture medium. Values denote carbon atoms and are normalized to 17 carbon atom influx into riboflavin. All values were multiplied by the number of carbon atoms of the reactants. Note that the model is simplified and cannot distinguish between carbon flux through e.g. gluconeogenesis or lower PP pathway as well as pyruvate dehydrogenase. Reaction between OAA/MAL and PEP/PYR pool is a lumped flux. Only reactions necessary for riboflavin biosynthesis were considered and all reactions represent net fluxes. Note that the conversion of citrate to isocitrate via aconitase most likely does not occur in the peroxisome [68]. 3PG, 3-phosphoplycerate; AcCoA_P/M_, peroxisomal/mitochondrial acetyl-CoA; AKG, α-ketoglutarate; ArP, 5-amino-6-ribitylamino-2,4(1H,3H)-pyrimidinedione; CH_2_-THF, 5,10-methylenetetrahydrofolate; CHO-THF, 10-formyltetrahydrofolate; FA, fatty acids (here: three C17.3 FA); FOR, formate; GAR, glycineamide ribonucleotide; GLU, glutamate; GLY, glycine; GLY_INTR_, intracellular glycine pool; GTP, guanosine triphosphate; PRA, 5-phosphoribosylamine; PYR, pyruvate; R5P, ribose 5-phosphate; Ru5P, ribulose 5-phosphate; RF_V_, riboflavin stored in the vacuole; SER, serine; THF, tetrahydrofolate; YE, yeast extract

**Fig. 9:**
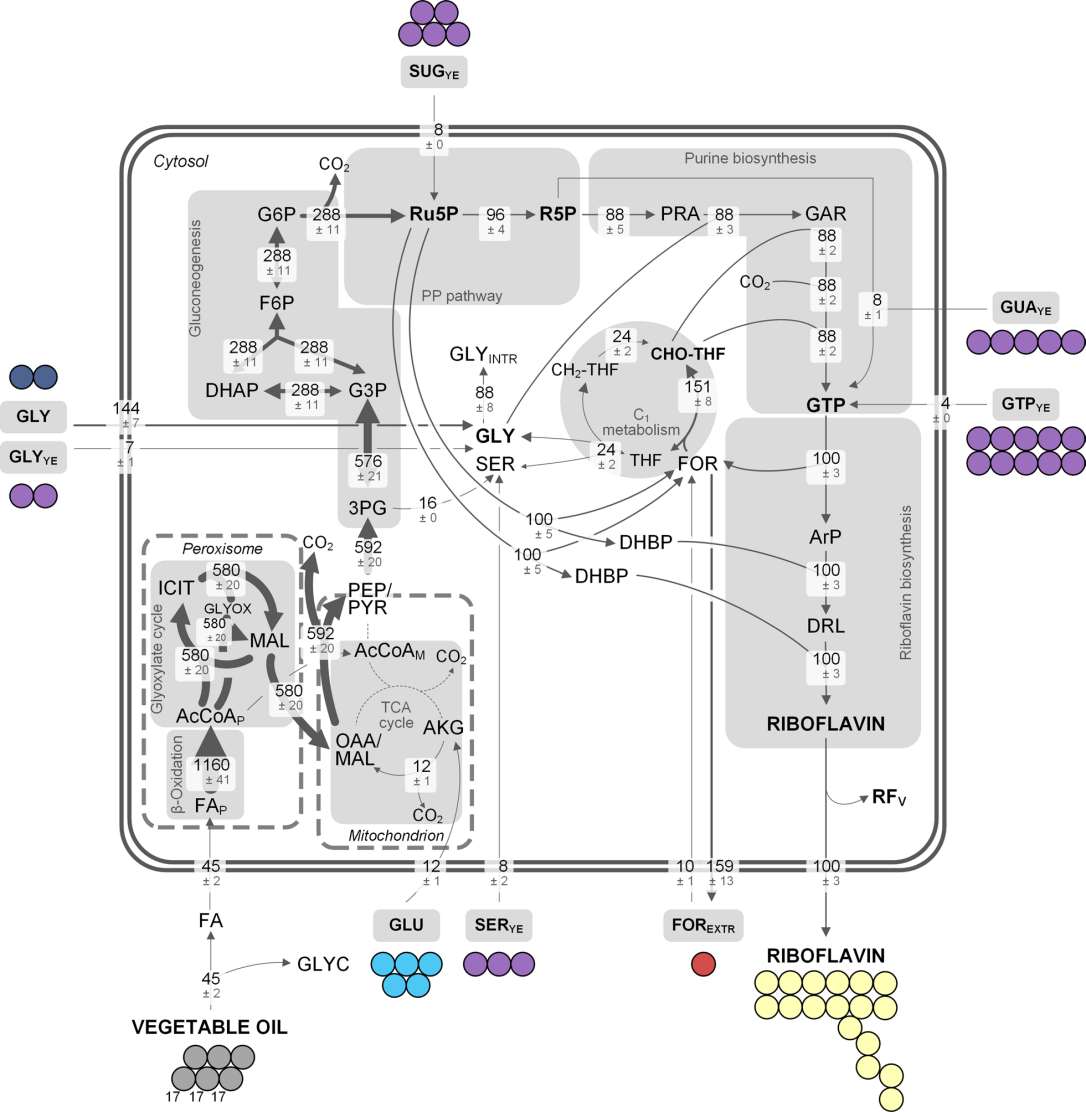
Intracellular carbon fluxes of riboflavin production with *A.* *gossypii* B2 on rapeseed oil and complex medium, which were determined by four parallel ^13^C-labeled tracer studies using [^13^C_2_] glycine, [^13^C] formate, [^13^C_5_] glutamate, and [U^13^C] yeast extract. The carbon fluxes were normalized to the specific riboflavin production rate (8.7 ± 2.3 µmol g^−1^ h^−1^), which was set to 100%. Riboflavin was obtained at the end of the growth phase of riboflavin producing *A.* *gossypii* after 144 h. Data are derived from positional ^13^C enrichment obtained from ^13^C NMR measurements (Table 3), corrected for natural labeling and dilution effects through unlabeled pre-culture medium. Note that the model is simplified and cannot distinguish between carbon flux through e.g. gluconeogenesis or lower PP pathway as well as pyruvate dehydrogenase. Reaction between OAA/MAL and PEP/PYR pool is a lumped flux. Only reactions necessary for riboflavin biosynthesis were considered and all reactions represent net fluxes. Note that some reactions as shown in Fig. 7 can be expressed as a single reaction, i.e. all decarboxylation reactions and in general all reactions with more than one educt or product can be expressed as a single flux. All fluxes from the medium into the cell are not fluxes based on concentrations, but solely derived from ^13^C labeling of riboflavin. Note that the conversion of citrate to isocitrate via aconitase most likely does not occur in the peroxisome [68]. 3PG, 3-phosphoplycerate; AcCoA_P/M_, peroxisomal/mitochondrial acetyl-CoA; AKG, α-ketoglutarate; ArP, 5-amino-6-ribitylamino-2,4(1H,3H)-pyrimidinedione; DRL, 6,7-dimethyl-8-ribityllumazine; CH_2_-THF, 5,10-methylenetetrahydrofolate; CHO-THF, 10-formyltetrahydrofolate; FA, fatty acids (here: three C17.3 FA); FOR, formate; GAR, glycineamide ribonucleotide; GLU, glutamate; GLY, glycine; GLY_INTR_, intracellular glycine pool; GTP, guanosine triphosphate; PRA, 5-phosphoribosylamine; PYR, pyruvate; R5P, ribose 5-phosphate; Ru5P, ribulose 5-phosphate; RF_V_, riboflavin stored in the vacuole; SER, serine; THF, tetrahydrofolate; YE, yeast extract

The integrated data set enabled the calculation of a metabolic flux map, comprising the respective contributions of single medium ingredients to riboflavin in this environment (Fig. 8). While the single carbon fluxes in Fig. 8 are expressed as single carbon atoms based on calculations as described above and in the Additional file 1, these fluxes were then converted into molar fluxes: first, each flux was divided by the number of carbon atoms of the respective metabolite, then, the flux into riboflavin was set to 100 ± 3% and represented the specific riboflavin production rate (8.7 ± 2.3 µmol g^−1^ h^−1^). Subsequently, all other fluxes were normalized to that flux into riboflavin. The resulting relative carbon fluxes are given in Fig. 9. Rapeseed oil was cleaved via the extracellular lipase into one mole glycerol and three moles fatty acids [24]. A high flux through the ß-oxidation pathway (1160 ± 41%) and the glyoxylate shunt (580 ± 20%) could be observed. Acetyl-CoA generated in the ß-oxidation pathway was then converted into malate through the glyoxylate shunt in order to enter gluconeogenesis and subsequently serve as carbon donor from vegetable oil for riboflavin. Therefore, all carbon in riboflavin that was derived from rapeseed oil, was assimilated via the glyoxylate shunt. Glutamate most likely entered the metabolism through α-ketoglutarate with a flux of 12 ± 1%, which resulted in a comparably low flux from α-ketoglutarate to oxaloacetate (12 ± 1%). A flux of 592 ± 20% was directed through the lower gluconeogenic pathway. Here, the map representing single carbon atoms (Fig. 8), highlights the loss of carbon (5.92 ± 0.20) via decarboxylation of the OAA/malate pool to the PEP/pyruvate pool. The assembly of two three-carbon units to one six-carbon molecule resulted in a decreased flux (288 ± 11%) in the upper gluconeogenesis (Fig. 9). Another decarboxylation reaction resulted in loss of carbon upon entry into the PP pathway (2.88 ± 0.11) (Fig. 8). The carbon flux of 288 ± 11% was split at ribulose 5-phosphate, an immediate precursor of riboflavin. The exact pathway used to synthesize this pool, gluconeogenesis or PP pathway, could not be distinguished by our approach. The combined carbon flux from ribulose 5-phosphate pool, which contributes eight carbon atoms in total to riboflavin, towards riboflavin was 200 ± 7%, with a corresponding carbon flux towards the intracellular formate pool of 100 ± 5% each. The second branch from ribulose 5-phosphate was directed into purine biosynthesis (96 ± 4%). The flux through the complete de novo biosynthesis of purines (GTP pool) equaled 88 ± 5%. Throughout that pathway, one-carbon units as well as carbon dioxide and glycine were incorporated into the final GTP molecule. Nucleotides from yeast extract (ATP or GTP) donated another 4 ± 0% to that pool. However, ^13^C labeling data also suggested that adenine or guanine from the supplemented yeast extract together with ribose 5-phosphate contributed 8 ± 1% to the GTP pool of riboflavin (Fig. 9). The carbon flux distribution highlights the dominant role of vegetable oil in riboflavin biosynthesis. Indeed, the overall flux of rapeseed oil into riboflavin equaled 13.8 ± 0.1 carbon atoms (81 ± 1% of the molecule). However, the contribution of other medium ingredients to riboflavin, especially to the pyrimidine ring, was as high as 19 ± 1%. This finding  included glycine and C_1_ donors in particular. As shown in our previous work, overcoming a limitation in the one-carbon pool, which makes up only 6% (1 carbon atom) of the vitamin, can have a great impact on product titers [16]. Consequently, the knowledge gained in such ^13^C tracer studies conveys a valuable starting point for rational strain engineering as well as carefully designed process control.

## Discussion

Riboflavin biosynthesis with *A.* *gossypii* is one of the success stories of biotechnology [8]. Due to the industrial importance of the fungus and its product, understanding the underlying metabolic pathways is the key step towards superior producer strains and in turn, meeting constantly increasing market demands. In this work, the complex medium component yeast extract was ^13^C-labeled for the first time and gave valuable insights into growth and production behavior of *A.* *gossypii*. In order to complement the data set and be able to fully trace carbon origin in riboflavin, glutamate was also added as fully labeled isotopomer. The ^13^C labeling data obtained in this study together with data gained in a previous study [16] allowed the calculation of carbon fluxes in the growth phase of *A.* *gossypii* on rapeseed oil and complex medium. In addition, a carbon flux map could be calculated for the riboflavin biosynthetic phase of the process using an industrial cultivation set-up. The data showed that while yeast extract was the main carbon source for growth (Fig. 3), its impact on riboflavin biosynthesis was still significant with an overall contribution of 8 ± 0%. In addition, glycine as two-carbon unit, which is readily incorporated into the vitamin, also made up 9 ± 0% of the product. By far the greatest impact had rapeseed oil (81 ± 1%). This highlights the importance of this carbon source for riboflavin production [45]. Although various other nutrients were present, the cells efficiently incorporated the oil into the product.

### Yeast extract efficiently supports growth of *A. gossypii*

As shown, large amounts of advanced building blocks such as amino acids were directly incorporated into cell constituents (Fig. 3). The organism saved significant amounts of anabolic precursors, redox power and energy by taking up costly building blocks from the medium. This drastically reduced the resources needed for growth. As example, the synthesis of one molecule of histidine requires 1 NADPH and 6 ATP, while isoleucine formation demands for 5 NADPH and 2 ATP in addition to carbon. Their uptake therefore displays an efficient way for the cell to save these resources. The benefit of this strategy is impressively visualized by the fact that the demand for NADPH (721 ± 62 µmol g^−1^) on the complex medium was only 7 ± 1% of the value, expected for growth on minimal medium (10,660 µmol g^−1^) (Table 1). Likewise, other microbes exploit yeast extract in a similar manner [46].

### Yeast extract-derived nucleotides and nucleobases contribute significantly to the formation of the vitamin

Yeast extract is a complex raw material that is often employed in large-scale industrial fermentation processes. However, there are large lot-to-lot variations of a single yeast extract, not to mention differences between yeast extracts produced with different strains or on different media [44, 47]. The recombinant production of immunoglobulin by *S.* *cerevisiae* was reported to be heavily dependent on a certain yeast extract [41]. Zhang et al. [41] especially identified adenine as a ‘principle component’ in the yeast extract that had a great influence on production performance of the strain used. In total, yeast extract contributed about 8% of carbon to riboflavin. The majority of which was contributed by nucleobases and nucleotides from the yeast extract as the ^13^C NMR data suggested. Most metabolic engineering efforts for improved riboflavin production have focused on increasing the precursor supply of glycine [19, 48] and GTP [49–52], which are valuable components of yeast extract. In addition, the supplementation of culture media with hypoxanthine was reported to be beneficial for riboflavin production [45]. Therefore, the yeast extract chosen for the production medium should be selected and screened carefully, since a nucleotide rich yeast extract would certainly be advantageous for an increased production performance. The efficient support of growth plus the donation of valuable product precursors underlines the importance of yeast extract as medium ingredient.

### *A. gossypii* displays high TCA cycle fluxes during growth and during riboflavin production

The integrated results from ^13^C tracer studies (Additional file 1: Tables S2, S3) revealed a high TCA cycle flux during the growth phase of *A.* *gossypii* B2 (Fig. 5). Likewise, the TCA cycle flux seemed high during riboflavin biosynthesis, also. The absence of growth and by-product formation (Fig. 2a) and the low overall yield for riboflavin biosynthesis on oil (Table 2), which equaled 0.09 ± 0.01 C-mol_RF_ C-mol_Oil_^−1^, i.e. 9 ± 1%, showed that 91% of carbon from rapeseed oil, which was taken up by the cell, was likely decarboxylated via a highly active TCA cycle (Fig. 5). This seemed surprising at first, given the resulting low demand of cellular ATP for growth and biosynthetic purposes (Table 2). Yet, the question remains, why the TCA cycle is so active. During growth, most cellular building blocks were taken up from the medium, thus resulting in a low ATP demand (Fig. 5). During riboflavin biosynthesis, ninefold more oil-based carbon was taken up than was used for the product. Little is known about the TCA cycle activity of riboflavin producing *A.* *gossypii* on vegetable oil or pathway activity of other microorganisms on oil as a substrate. It was shown that *A.* *gossypii* accumulates intracellular *trans*-aconitate during growth [53]. This known inhibitor of the enzyme aconitase, which catalyzes the conversion of citrate to isocitrate via the intermediate cis-aconitate, apparently is formed spontaneously from cis-aconitate [53]. During the riboflavin production phase, expression levels of another enzyme, *trans*-aconitate 3-methyltransferase, were increased nearly threefold compared to the growth phase. Through methylation of *trans*-aconitate, this enzyme relieved the inhibition of the TCA cycle enzyme aconitase and consequently, enabled an increased flux through the TCA cycle as well as the glyoxylate shunt [53]. In a study with mice that were put on a high-fat diet, liver metabolism was investigated [54]. The consequences of the fatty diet were increasing hepatic insulin resistance due to the lipid overload, but also an increased TCA cycle as well as elevated gluconeogenesis. The increased flux through the TCA cycle possibly compensated for an inefficiently coupled oxygen consumption and ATP synthesis, i.e. a dysfunctional mitochondrial respiration. The authors also observed the production of reactive oxygen species, thereby linking the induction of a highly active TCA cycle to oxidative stress [54]. Indeed, mitochondria produce reactive oxygen species when e.g. the NADH pool is reduced as a result of low ATP demand [55]. This is especially interesting, because riboflavin biosynthesis in *A.* *gossypii* is associated with oxidative stress [56, 57]. These studies provide evidence for an increased TCA cycle flux during growth on lipid-rich media, which might also apply for *A.* *gossypii*. In this regard, the high TCA cycle activity could compensate for a high maintenance demand. In a ^13^C metabolic flux study with *Sorangium cellulosum*, a slow growing myxobacterium, a high flux through the TCA cycle was linked to a high maintenance metabolism [58]. Wild type *B.* *subtilis* also requires a lot of energy for its maintenance [59]. However, knockout of the terminal cytochrome *bd* oxidase in the respiratory chain of a riboflavin producing *Bacillus* strain, resulted in more efficient proton pumping, thereby decreasing the high maintenance metabolism, and thus, even improving riboflavin production performance [60]. While these connections offer interesting starting points for a deeper assessment of the underlying mechanisms, further work will undoubtedly be needed to resolve the fine structures of riboflavin biosynthesis with *A.* *gossypii* on rapeseed oil.

### The low-flux oxidative PP pathway does not supply sufficient redox power

An interesting characteristic was the reduced flux through the upper metabolism, i.e. gluconeogenesis and oxidative PP pathway. This pathway, comprising the NADPH-supplying enzymes glucose-6-phosphate dehydrogenase and phosphogluconate dehydrogenase is often regarded as main donor for cytosolic NADPH [61]. However, the flux through this route (up to 0.002 mmol g^−1^ h^−1^) (Fig. 5) was far below the resulting anabolic demand, corrected for uptake of medium ingredients (0.058 ± 0.005 mmol g^−1^ h^−1^) (Table 1). Thus, only 2–3% of NADPH required for growth was covered through the oxidative PP pathway, raising the need for an alternative NADPH supply in the cell. There are different potentially NADPH-generating candidates in *A.* *gossypii*: isocitrate dehydrogenase (IDH), malic enzyme (MaE), and NADH kinase. In *A.* *gossypii* there are two isoforms of the IDH, which converts isocitrate into α-ketoglutarate: the mitochondrial isoenzyme is NAD-specific, whereas the NADP-dependent enzyme is localized to the peroxisome [62], where it is thought to be involved in cofactor regeneration during oxidation of unsaturated fatty acids via 2,4-dienoyl-CoA reductase [62]. The enzyme for the decarboxylation of malate to pyruvate, catalyzed by MaE, is annotated for *A.* *gossypii* (KEGG, AGL068 W), however, no experimental data for the cofactor specificity are available in the literature [35–37]. The MaE of *Aspergillus niger* was found to be NADP-dependent [63], whereas the *S.* *cerevisiae* enzyme was reported to use both NAD and NADP [64]. Another NADPH-providing reaction is the phosphorylation of NADH by NADH kinase. This reaction is located in the mitochondria and was described, among others, for *S.* *cerevisiae* and *Aspergillus nidulans* [61, 65] and is annotated for *A.* *gossypii* (KEGG, AFL063W) [35].

## Conclusion

A detailed analysis of carbon fluxes during growth and riboflavin production in a very complex cultivation set-up using *A.* *gossypii* was presented in this work. The ubiquitous industrial medium ingredient yeast extract was ^13^C-labeled for the first time and thus, its impact on the metabolism studied in greater detail. Yeast extract could be identified as the main carbon source during growth, while rapeseed oil was the main carbon source during riboflavin production. However, the ^13^C labeling data and resulting carbon fluxes demonstrated that yeast extract also contributed a significant amount of carbon to the product. Other industrial processes also rely heavily on yeast extract as complex nitrogen source [21, 41]. The industrial production of l-lysine with bacteria uses yeast extract as medium ingredient [66] as well as the commercial antibiotics fermentation with filamentous fungi [21, 22]. *Bacillus thuringiensis* is industrially employed as insecticide producer, a process which depends on complex compounds such as corn steep liquor or soybean meal. Since those ingredients might not be readily available at every production site, yeast extract was tested as alternative complex nutrient [67]. These studies highlight the importance of complex media for industrial fermentation processes and the need for a more sophisticated experimental set-up in order to study and improve such systems in great detail. The ^13^C labeling approach presented here, provides an excellent and promising starting point for the improvement of other microbial processes under complex and commercial cultivation conditions.

## Additional file

**Additional file 1.** Additional figures and tables.

## Abbreviations

3PG: 3-phosphoglycerate
AcCoA: acetyl-CoA
AKG: α-ketoglutarate
Ala: alanine
Arg: arginine
ArP: 5-amino-6-ribitylamino-2,4(1H,3H)-pyrimidinedione
Asp: aspartate
CH_2_-THF: 5,10-methylenetetrahydrofolate
CHO-THF: 10-formyltetrahydrofolate
Cit: citrate
DHAP: dihydroxyacetone phosphate
E4P: erythrose 4-phosphate
F6P: fructose 6-phosphate
FA: fatty acid
For: formate
G3P: glyceraldehyde 3-phosphate
G6P: glucose 6-phosphate
GAR: glycineamide ribonucleotide
Gln: glutamine
Glu: glutamate
Gly: glycine
Glyc: glycerol
Glyox: glyoxylate
GTP: guanosine triphosphate
His: histidine
Icit: isocitrate
IDH: isocitrate dehydrogenase
Ile: isoleucine
Leu: leucine
MaE: malic enzyme
Mal: malate
MID: mass isotopomer distribution
OAA: oxaloacetate
P: product
PEP: phosphoenolpyruvate
Phe: phenylalanine
PP pathway: pentose phosphate pathway
Pro: proline
PYR: pyruvate
R5P: ribose 5-phosphate
RF: riboflavin
Ru5P: ribulose 5-phosphate
S: substrate
S7P: sedoheptulose 7-phosphate
Ser: serine
SFL: summed fractional labeling
TCA cycle: tricarboxylic acid cycle
THF: tetrahydrofolate
Thr: threonine
Tyr: tyrosine
Val: valine
X: biomass
Xu5P: xylulose 5-phosphate
YE: yeast extract
