# Supplementary material for: Metabolic flux analysis in Ashbya gossypii using 13C-labeled yeast extract: industrial riboflavin production under complex nutrient conditions
Source: Microb Cell Fact. 2018 Oct 16;17:162. doi: 10.1186/s12934-018-1003-y (PMC6190667; doi:10.1186/s12934-018-1003-y)
Supplement: Supplementary file 1 — Additional file 1. Additional figures and tables. [file 12934_2018_1003_MOESM1_ESM.pdf]

Additional file to

Metabolic flux analysis in *Ashbya gossypii* using ^{13}C-labeled yeast extract: industrial riboflavin production under complex nutrient conditions

Susanne Katharina Schwechheimer[1], Judith Becker[1], Lindsay Peyriga[2,3,4], Jean-Charles Portais[2,3,4], Christoph Wittmann[1*]

[1] Institute of Systems Biotechnology, Saarland University, Germany

[2] Université de Toulouse; INSA, UPS, INP, Toulouse, France

[3]INRA, UMR792 Ingénerie des Systèmes Biologiques et des Procédés, Toulouse, France

[4]CNRS, UMR5504, Toulouse, France

susanne.schwechheimer@uni-saarland.de

judith.becker@uni-saarland.de

peyriga@insa-toulouse.fr

portais@insa-toulouse.fr

christoph.wittmann@uni-saarland.de

[*]Corresponding address: Campus A1.5, 66123 Saarbrücken, Germany, christoph.wittmann@uni-saarland.de, Phone: +49-681-302-71970, FAX: +49-681-302-71972.

Correction of ^{13}C labeling data from hydrolyzed cell protein and glycogen

As described before [1], all MIDs were corrected for natural isotopes [2] as well as for the fraction of naturally labeled amino acids originating from the inoculum. The output of the correction was a mass isotopomer distribution of the carbon skeleton of the analytes formed during the corresponding isotope experiment (MID_{corr}). The total ^{13}C enrichment of the carbon skeleton of a compound with *n* carbon atoms, termed summed fractional labeling (SFL) [3], was calculated according to Equation (S1). The data were given in percent, whereby 100 % represents a fully ^{13}C-labeled carbon backbone and *i* represents the number of ^{13}C atoms of this isotopomer.

$$SFL = \sum_{i=1}^{n+1} \frac{i \cdot MID_{i,corr}}{n} \cdot 100 \tag{S1}$$

To calculate the net incorporation of ^{13}C label from the tracers, the SFL was corrected for the natural ^{13}C background of the carbon backbone (1.07 %) [4] as well as fraction of labeling originating from unlabeled pre-culture of the respective tracer at the beginning of the cultivation (**Fig. S1**). Equation (S2) then yielded the corrected SFL_{corr} (all SFLs are given in percent).

$$SFL_{corr} = \frac{SFL - SFL_{nat}}{SFL_{Tr} - SFL_{Tr,nat}} \cdot 100 \tag{S2}$$

with

SFL_{corr} for natural background and dilution of ^{13}C tracer enrichment through pre-culture corrected SFL of a proteinogenic amino acid, glucose or riboflavin [%]

SFL	SFL of a proteinogenic amino acid or riboflavin calculated according to Equation (1) [%]
SFL_{nat}	SFL of proteinogenic amino acid or riboflavin when synthesized on naturally labeled medium [%]
SFL_{Tr}	SFL of the respective tracer at the beginning of the cultivation (0 h), when glycine, formate, glutamate, and yeast extract are fully labeled [%]
$SFL_{Tr,nat}$	SFL of the naturally labeled respective tracer [%]

Correction of LC/MS and ^{13}C NMR data of riboflavin

LC/MS derived MIDs for the 377 *m/z* parent ion of riboflavin were corrected for the natural abundance of isotopes [2]. SFLs were derived according to Equations (S1) and (S2) as described above and before [1].

Positional ^{13}C enrichment data ($\%^{13}C_{exp}$) of single carbon atoms, derived from ^{13}C NMR measurements were corrected in a similar manner according to Equation (S3).

$$\%^{13}C_{corr} = \frac{\%^{13}C_{exp} - \%^{13}C_{nat}}{SFL_{Tr} - SFL_{Tr,nat}} \cdot 100 \tag{S3}$$

with

$\%^{13}C_{corr}$	the real ^{13}C enrichment of the carbon of interest of riboflavin from a given tracer [%]
$\%^{13}C_{exp}$	measured ^{13}C enrichment for carbon atom of interest of riboflavin from a given tracer [%]
$\%^{13}C_{nat}$	the natural ^{13}C enrichment of the carbon of interest [%]

SFL_{Tr}	SFL of the respective tracer at the beginning of the cultivation (0 h), when glycine, formate, glutamate, and yeast extract are fully labeled [%]
$SFL_{Tr,nat}$	SFL of the naturally labeled respective tracer [%]

Glutamate-derived proteinogenic amino acids are not entirely taken up from ^{13}C-labeled yeast extract

As shown, the proteinogenic amino acids alanine, aspartate, and glutamate were less ^{13}C-enriched compared to the other amino acids (**Fig. 3, Fig. 4, Table S3**), when cells were grown on [$U^{13}C$] yeast extract. Of the three, proteinogenic alanine exhibited the largest fully labeled isotopomer (M+3) fraction with 61 ± 6 %. Unlabeled alanine from cell protein made up 31 ± 2 % of the intracellular pool (**Fig. 4a**). The majority of aspartate was non-labeled, however, as much as 30 ± 2 % of the pool were fully labeled (M+4), which indicated origin from the fully ^{13}C-labeled yeast extract (**Fig. 4b**). The intracellular glutamate pool was by far the least ^{13}C-labeled of those three amino acids: the unlabeled mass isotopomer (M+0) made up 73 ± 7 % of the amino acid, thereby being reduced only 1.3-fold compared to the naturally labeled control (**Fig. 4c**). While also the single labeled mass isotopomer (M+1) was slightly enriched, fully labeled glutamate (M+5) contributed to only 9 ± 1 % of the proteinogenic pool (**Fig. 4c**). Thus, fully ^{13}C-labeled yeast extract had only a marginal impact on the intracellular glutamate pool and a large percentage was derived from other medium ingredients, i.e. glutamate or vegetable oil. Considering the obvious tight regulation of amino acid biosynthesis observed for the majority of amino acids highlighted by the addition of [$U^{13}C$] yeast extract as well as glycine and serine as shown in our previous study [1], exogenous glutamate added to the medium might be the precursor of the intracellular alanine, aspartate, and glutamate pools. Therefore, [$^{13}C_5$] glutamate replaced the naturally labeled amino acid in the culture medium in a second experiment. Close inspection of the cell protein

which was harvested at the end of the exponential growth phase (36 h), formed under these conditions revealed only a minor contribution of the exogenous glutamate to the intracellular proteinogenic pools (**Fig. 3, Fig. 4, Table S3**). The added ^{13}C-labeled glutamate contributed merely 12 ± 1 % (SFL_{corr}) to the respective intracellular pool. This was highlighted by the small fraction of fully labeled glutamate from cell protein: only 8 % displayed the M+5 mass isotopomer. Unlabeled glutamate made up 78 % of the intracellular pool (**Fig. 4f**). Aspartate was even less ^{13}C-labeled with a SFL_{corr} of 5 ± 0 %. Here, the unlabeled mass isotopomer was 1.1-fold reduced compared to the naturally labeled control. While the single (M+1) and double labeled (M+2) mass isotopomers were elevated, the fully ^{13}C-labeled aspartate was only slightly enriched (**Fig. 4e**). Alanine showed an enrichment of 2 ± 0 % (**Table S3**) and exhibited almost the same mass isotopomer distribution as the naturally labeled control (**Fig. 4**). These data confirmed that, while glutamate was taken up by the cell and present in large amounts in the medium (**Fig. 2**), *de novo* biosynthesis of the amino acid seems to be active.

The positional ^{13}C enrichment of riboflavin, which was synthesized by *A. gossypii* B2 grown on complex medium with fully ^{13}C-labeled glutamate, was analyzed via NMR. The obtained ^{13}C NMR data of riboflavin revealed that glutamate contributed ^{13}C labeling to almost all carbon atoms in riboflavin (**Table 3**, **Fig. 6a**). These data underlined that glutamate, which is not an immediate riboflavin precursor, is tightly connected with the α-ketoglutarate pool and thus, contributed to the carbon core metabolism via the TCA cycle. This is highlighted by the ^{13}C enrichment of the amino acids aspartate and

alanine from [$^{13}C_5$] glutamate, which are derived from different metabolic precursors (**Fig. 4**). Hence, the strong *de novo* biosynthesis of glutamate diluted the ^{13}C labeling of the intracellular pool. This resulted in only slight ^{13}C enrichment of the respective carbon atoms of riboflavin: 3.1 ± 0.1 %, 1.7 ± 0.0 %, and 0.7 ± 0.0 % on average for ribityl side chain, xylene ring, and pyrimidine ring, respectively (**Fig. 6a**).

Experimental approach to assess the direct incorporation of complex building blocks into riboflavin

In our previous study, the addition of labeled glycine, formate, and serine gave valuable insights into the riboflavin metabolism of *A. gossypii* B2 [1]. However, rapeseed oil and yeast extract, the two most abundant medium ingredients for the riboflavin production with *A. gossypii*, remained to be tested. Since ^{13}C-labeled rapeseed oil was unavailable, custom-synthesized fully labeled yeast extract studies were conducted. In order to be able to distinguish between incorporation of ^{13}C labeling during the growth and subsequent riboflavin production phase, two cultivations were carried out in parallel: (i) growth on fully labeled yeast extract until 32 h and (ii) growth on naturally labeled yeast extract until 32 h. Once growth had ceased and cells started to accumulate riboflavin, the cultures were centrifuged and the medium was exchanged. That way it could be distinguished, whether ^{13}C-labeled yeast extract-based compounds were incorporated into riboflavin in the early riboflavin production phase (the first 32 h of cultivation) or if the incorporation occurred after growth had ceased (32 h to 144 h). At the end of the cultivation (144 h), the positional ^{13}C enrichment of riboflavin was analyzed via ^{13}C NMR.

Data from GC/MS analyses

Table S1: Summed fractional labeling (SFL) of amino acids from hydrolyzed [$U^{13}C$] yeast extract from the company Ohly (Hamburg, Germany). The relative average error for the labeling measurement was below 1 %.

**Analyte**	**SFL [%]**
Ala_260	98.6
Gly_246	98.4
Val_288	98.7
Leu_274	98.7
Ile_274	98.4
Ser_390	98.6
Thr_404	98.4
Phe_336	99.8
Asp_418	98.5
Glu_432	98.7
Lys_431	98.3
Arg_442	98.5
Tyr_466	99.7
His_440	98.1

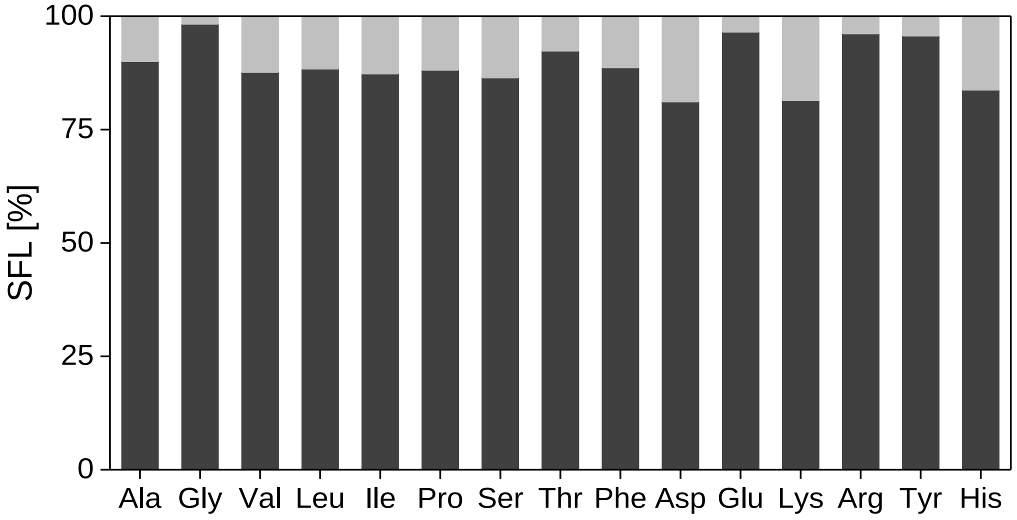

Figure S1: Summed fractional labeling (SFL) derived from GC/MS measurements of the culture supernatant of *A. gossypii* B2 cells grown on complex medium with [$U^{13}C$] yeast extract, [$^{13}C_2$] glycine, [^{13}C] formate, and [$^{13}C_5$] glutamate in a single experiment. Labeling data derived from that cultivation are depicted in dark grey while the light grey bars indicate the resulting contribution of non-labeled pre-culture medium to the supernatant. Data were obtained from three individual replicates and denote mean values with a mean standard deviation of 5 %.

Table S2: Relative mass isotopomer fractions of amino acids from hydrolyzed cell protein of *A. gossypii* B2 grown on naturally labeled vegetable oil and 99 % [$^{13}C_2$] glycine, [$^{13}C_3$] serine, or [^{13}C] formate. Cultivation of *A. gossypii* on naturally labeled medium served as control. Data denote corrected labeling patterns. Data were corrected for fraction of unlabeled biomass in the inoculum as well as occurrence of natural isotopes. The mass isotopomer M+0 represents the relative amount of non-labeled, M+1 the amount of singly-labeled mass isotopomer fraction and so on. Data were obtained from three individual replicates in our previous study [1]. In some cases of compounds only very little or even non-enriched in ^{13}C, the correction yielded negative SFL values. In these cases, the SFL was corrected to 0.00.

**Analyte**		**Control**	**[$^{13}C_2$] Gly**	**[^{13}C] For**	**[$^{13}C_3$] Ser**
Ala_260	M+0	0.96 ± 0.00	0.91 ± 0.00	0.91 ± 0.00	0.92 ± 0.00
	M+1	0.03 ± 0.00	0.03 ± 0.00	0.09 ± 0.00	0.04 ± 0.00
	M+2	0.00 ± 0.00	0.06 ± 0.00	0.00 ± 0.00	0.00 ± 0.00
	M+3	0.00 ± 0.00	0.00 ± 0.00	0.00 ± 0.00	0.03 ± 0.00
	SFL [%]	1.15 ± 0.08	5.17 ± 0.11	3.01 ± 0.11	4.89 ± 0.11
	**SFL_{corr} [%]**	**0.08 ± 0.01**	**4.22 ± 0.08**	**2.00 ± 0.07**	**4.05 ± 0.09**
Gly_246	M+0	0.98 ± 0.00	0.15 ± 0.06	0.98 ± 0.00	0.89 ± 0.12
	M+1	0.02 ± 0.00	0.02 ± 0.00	0.02 ± 0.00	0.02 ± 0.00
	M+2	0.00 ± 0.00	0.83 ± 0.11	0.00 ± 0.00	0.08 ± 0.01
	SFL [%]	1.15 ± 0.08	84.07 ± 10.64	1.09 ± 0.16	9.44 ± 1.13
	**SFL_{corr} [%]**	**0.08 ± 0.01**	**85.41 ± 10.81**	**0.16 ± 0.02**	**8.92 ± 1.07**
Val_288	M+0	0.95 ± 0.00	0.94 ± 0.00	0.94 ± 0.00	0.94 ± 0.00
	M+1	0.05 ± 0.00	0.05 ± 0.00	0.05 ± 0.00	0.05 ± 0.00
	M+2	0.00 ± 0.00	0.00 ± 0.00	0.00 ± 0.00	0.00 ± 0.00
	M+3	0.00 ± 0.00	0.00 ± 0.00	0.00 ± 0.00	0.00 ± 0.00
	M+4	0.00 ± 0.00	0.00 ± 0.00	0.00 ± 0.00	0.00 ± 0.00
	M+5	0.00 ± 0.00	0.00 ± 0.00	0.00 ± 0.00	0.00 ± 0.00
	SFL [%]	1.12 ± 0.03	1.3 ± 0.04	1.24 ± 0.13	1.24 ± 0.11
	**SFL_{corr} [%]**	**0.05 ± 0.00**	**0.21 ± 0.01**	**0.15 ± 0.02**	**0.15 ± 0.01**
Leu_302	M+0	0.93 ± 0.00	0.93 ± 0.00	0.93 ± 0.00	0.93 ± 0.00
	M+1	0.07 ± 0.00	0.07 ± 0.00	0.06 ± 0.00	0.07 ± 0.00
	M+2	0.00 ± 0.00	0.00 ± 0.00	0.00 ± 0.00	0.00 ± 0.00
	M+3	0.00 ± 0.00	0.00 ± 0.00	0.00 ± 0.00	0.00 ± 0.00
	M+4	0.00 ± 0.00	0.00 ± 0.00	0.00 ± 0.00	0.00 ± 0.00
	M+5	0.00 ± 0.00	0.00 ± 0.00	0.00 ± 0.00	0.00 ± 0.00
	M+6	0.00 ± 0.00	0.00 ± 0.00	0.00 ± 0.00	0.00 ± 0.00
	SFL [%]	1.16 ± 0.04	1.17 ± 0.01	1.21 ± 0.08	1.21 ± 0.10
	**SFL_{corr} [%]**	**0.09 ± 0.00**	**0.07 ± 0.00**	**0.12 ± 0.01**	**0.12 ± 0.01**

Table S2: Relative mass isotopomer fractions of amino acids from hydrolyzed cell protein of *A. gossypii* B2 grown on naturally labeled vegetable oil and 99 % [$^{13}C_2$] glycine, [$^{13}C_3$] serine, or [^{13}C] formate **(continued)**.

**Analyte**		**Control**	**[$^{13}C_2$] Gly**	**[^{13}C] For**	**[$^{13}C_3$] Ser**
Ile_302	M+0	0.93 ± 0.00	0.93 ± 0.00	0.93 ± 0.00	0.93 ± 0.00
	M+1	0.06 ± 0.00	0.07 ± 0.00	0.07 ± 0.00	0.06 ± 0.00
	M+2	0.00 ± 0.00	0.00 ± 0.00	0.00 ± 0.00	0.00 ± 0.00
	M+3	0.00 ± 0.00	0.00 ± 0.00	0.00 ± 0.00	0.00 ± 0.00
	M+4	0.00 ± 0.00	0.00 ± 0.00	0.00 ± 0.00	0.00 ± 0.00
	M+5	0.00 ± 0.00	0.00 ± 0.00	0.00 ± 0.00	0.00 ± 0.00
	M+6	0.00 ± 0.00	0.00 ± 0.00	0.00 ± 0.00	0.00 ± 0.00
	SFL [%]	1.15 ± 0.08	1.19 ± 0.01	1.20 ± 0.03	1.20 ± 0.10
	**SFL_{corr} [%]**	**0.05 ± 0.00**	**0.09 ± 0.00**	**0.10 ± 0.00**	**0.11 ± 0.01**
Pro_286	M+0	0.94 ± 0.00	0.95 ± 0.00	0.94 ± 0.00	0.95 ± 0.00
	M+1	0.05 ± 0.00	0.05 ± 0.00	0.06 ± 0.00	0.05 ± 0.00
	M+2	0.00 ± 0.00	0.00 ± 0.00	0.00 ± 0.00	0.00 ± 0.00
	M+3	0.00 ± 0.00	0.00 ± 0.00	0.00 ± 0.00	0.00 ± 0.00
	M+4	0.00 ± 0.00	0.00 ± 0.00	0.00 ± 0.00	0.00 ± 0.00
	M+5	0.00 ± 0.00	0.00 ± 0.00	0.00 ± 0.00	0.00 ± 0.00
	SFL [%]	1.09 ± 0.12	1.05 ± 0.05	1.13 ± 0.02	1.13 ± 0.01
	**SFL_{corr} [%]**	**0.02 ± 0.00**	**0.00 ± 0.00**	**0.03 ± 0.00**	**0.03 ± 0.00**
Ser_390	M+0	0.97 ± 0.00	0.37 ± 0.03	0.57 ± 0.02	0.29 ± 0.04
	M+1	0.03 ± 0.00	0.02 ± 0.00	0.43 ± 0.03	0.14 ± 0.02
	M+2	0.00 ± 0.00	0.6 ± 0.03	0.01 ± 0.00	0.04 ± 0.01
	M+3	0.00 ± 0.00	0.01 ± 0.00	0.00 ± 0.00	0.53 ± 0.07
	SFL [%]	1.05 ± 0.11	41.59 ± 2.29	14.68 ± 0.85	60.13 ± 3.61
	**SFL_{corr} [%]**	**0.00 ± 0.00**	**41.67 ± 2.29**	**14.25 ± 0.85**	**63.13 ± 3.79**
Thr_404	M+0	0.96 ± 0.00	0.96 ± 0.00	0.96 ± 0.00	0.95 ± 0.00
	M+1	0.04 ± 0.00	0.04 ± 0.00	0.04 ± 0.00	0.04 ± 0.00
	M+2	0.00 ± 0.00	0.00 ± 0.00	0.00 ± 0.00	0.00 ± 0.00
	M+3	0.00 ± 0.00	0.00 ± 0.00	0.00 ± 0.00	0.00 ± 0.00
	M+4	0.00 ± 0.00	0.00 ± 0.00	0.00 ± 0.00	0.01 ± 0.00
	SFL [%]	1.07 ± 0.03	1.21 ± 0.03	1.04 ± 0.03	1.04 ± 0.01
	**SFL_{corr} [%]**	**0.00 ± 0.00**	**0.11 ± 0.00**	**0.00 ± 0.00**	**0.00 ± 0.00**

Table S2: Relative mass isotopomer fractions of amino acids from hydrolyzed cell protein of *A. gossypii* B2 grown on naturally labeled vegetable oil and 99 % [$^{13}C_2$] glycine, [$^{13}C_3$] serine, or [^{13}C] formate **(continued)**.

**Analyte**		**Control**	**[$^{13}C_2$] Gly**	**[^{13}C] For**	**[$^{13}C_3$] Ser**
Phe_336	M+0	0.91 ± 0.00	0.90 ± 0.00	0.91 ± 0.00	0.90 ± 0.00
	M+1	0.09 ± 0.00	0.08 ± 0.00	0.09 ± 0.00	0.09 ± 0.00
	M+2	0.00 ± 0.00	0.00 ± 0.00	0.00 ± 0.00	0.00 ± 0.00
	M+3	0.00 ± 0.00	0.00 ± 0.00	0.00 ± 0.00	0.00 ± 0.00
	M+4	0.00 ± 0.00	0.00 ± 0.00	0.00 ± 0.00	0.00 ± 0.00
	M+5	0.00 ± 0.00	0.01 ± 0.00	0.00 ± 0.00	0.00 ± 0.00
	M+6	0.00 ± 0.00	0.00 ± 0.00	0.00 ± 0.00	0.00 ± 0.00
	M+7	0.00 ± 0.00	0.00 ± 0.00	0.00 ± 0.00	0.00 ± 0.00
	M+8	0.00 ± 0.00	0.00 ± 0.00	0.00 ± 0.00	0.00 ± 0.00
	M+9	0.00 ± 0.00	0.00 ± 0.00	0.00 ± 0.00	0.00 ± 0.00
	SFL [%]	1.06 ± 0.03	1.39 ± 0.33	1.06 ± 0.01	1.06 ± 0.03
	**SFL_{corr} [%]**	**0.00 ± 0.00**	**0.30 ± 0.07**	**0.00 ± 0.00**	**0.00 ± 0.00**
Asp_418	M+0	0.96 ± 0.00	0.96 ± 0.00	0.96 ± 0.00	0.95 ± 0.00
	M+1	0.04 ± 0.00	0.04 ± 0.00	0.04 ± 0.00	0.04 ± 0.00
	M+2	0.00 ± 0.00	0.00 ± 0.00	0.00 ± 0.00	0.00 ± 0.00
	M+3	0.00 ± 0.00	0.00 ± 0.00	0.00 ± 0.00	0.00 ± 0.00
	M+4	0.00 ± 0.00	0.00 ± 0.00	0.00 ± 0.00	0.00 ± 0.00
	SFL [%]	0.99 ± 0.08	1.17 ± 0.05	1.13 ± 0.10	1.13 ± 0.05
	**SFL_{corr} [%]**	**0.00 ± 0.00**	**0.07 ± 0.00**	**0.03 ± 0.00**	**0.03 ± 0.00**
Glu_432	M+0	0.94 ± 0.00	0.94 ± 0.00	0.94 ± 0.00	0.94 ± 0.00
	M+1	0.05 ± 0.00	0.06 ± 0.00	0.05 ± 0.00	0.06 ± 0.00
	M+2	0.00 ± 0.00	0.00 ± 0.00	0.00 ± 0.00	0.00 ± 0.00
	M+3	0.00 ± 0.00	0.00 ± 0.00	0.00 ± 0.00	0.00 ± 0.00
	M+4	0.00 ± 0.00	0.00 ± 0.00	0.00 ± 0.00	0.00 ± 0.00
	M+5	0.00 ± 0.00	0.00 ± 0.00	0.00 ± 0.00	0.00 ± 0.00
	SFL [%]	1.10 ± 0.10	1.30 ± 0.11	1.29 ± 0.10	1.31 ± 0.09
	**SFL_{corr} [%]**	**0.03 ± 0.03**	**0.21 ± 0.02**	**0.20 ± 0.02**	**0.22 ± 0.02**
Lys_431	M+0	0.90 ± 0.02	0.94 ± 0.00	0.94 ± 0.00	0.94 ± 0.00
	M+1	0.06 ± 0.00	0.06 ± 0.00	0.05 ± 0.00	0.06 ± 0.00
	M+2	0.04 ± 0.02	0.00 ± 0.00	0.01 ± 0.00	0.00 ± 0.00
	M+3	0.00 ± 0.00	0.00 ± 0.00	0.00 ± 0.00	0.00 ± 0.00
	M+4	0.00 ± 0.00	0.00 ± 0.00	0.00 ± 0.00	0.00 ± 0.00
	M+5	0.00 ± 0.00	0.00 ± 0.00	0.00 ± 0.00	0.00 ± 0.00
	M+6	0.00 ± 0.00	0.00 ± 0.00	0.00 ± 0.00	0.00 ± 0.00
	SFL [%]	1.77 ± 0.68	1.06 ± 0.05	1.06 ± 0.09	1.06 ± 0.01
	**SFL_{corr} [%]**	**0.70 ± 0.27**	**0.00 ± 0.00**	**0.00 ± 0.00**	**0.00 ± 0.00**

Table S2: Relative mass isotopomer fractions of amino acids from hydrolyzed cell protein of *A. gossypii* B2 grown on naturally labeled vegetable oil and 99 % [$^{13}C_2$] glycine, [$^{13}C_3$] serine, or [^{13}C] formate **(continued)**.

**Analyte**		**Control**	**[$^{13}C_2$] Gly**	**[^{13}C] For**	**[$^{13}C_3$] Ser**
Arg_442	M+0	0.94 ± 0.00	0.94 ± 0.00	0.93 ± 0.00	0.93 ± 0.00
	M+1	0.06 ± 0.00	0.06 ± 0.00	0.07 ± 0.00	0.06 ± 0.00
	M+2	0.00 ± 0.00	0.00 ± 0.00	0.00 ± 0.00	0.00 ± 0.00
	M+3	0.00 ± 0.00	0.00 ± 0.00	0.00 ± 0.00	0.00 ± 0.00
	M+4	0.00 ± 0.00	0.00 ± 0.00	0.00 ± 0.00	0.00 ± 0.00
	M+5	0.00 ± 0.00	0.00 ± 0.00	0.00 ± 0.00	0.00 ± 0.00
	M+6	0.00 ± 0.00	0.00 ± 0.00	0.00 ± 0.00	0.00 ± 0.00
	SFL [%]	1.12 ± 0.06	1.08 ± 0.06	1.11 ± 0.09	1.11 ± 0.09
	**SFL_{corr} [%]**	**0.05 ± 0.00**	**0.00 ± 0.00**	**0.01 ± 0.00**	**0.01 ± 0.00**
Tyr_466	M+0	0.92 ± 0.00	0.90 ± 0.00	0.91 ± 0.00	0.90 ± 0.00
	M+1	0.07 ± 0.00	0.09 ± 0.00	0.09 ± 0.00	0.09 ± 0.00
	M+2	0.00 ± 0.00	0.01 ± 0.00	0.00 ± 0.00	0.01 ± 0.00
	M+3	0.00 ± 0.00	0.00 ± 0.00	0.00 ± 0.00	0.00 ± 0.00
	M+4	0.00 ± 0.00	0.00 ± 0.00	0.00 ± 0.00	0.00 ± 0.00
	M+5	0.00 ± 0.00	0.00 ± 0.00	0.00 ± 0.00	0.00 ± 0.00
	M+6	0.00 ± 0.00	0.00 ± 0.00	0.00 ± 0.00	0.00 ± 0.00
	M+7	0.00 ± 0.00	0.00 ± 0.00	0.00 ± 0.00	0.00 ± 0.00
	M+8	0.00 ± 0.00	0.00 ± 0.00	0.00 ± 0.00	0.00 ± 0.00
	M+9	0.00 ± 0.00	0.00 ± 0.00	0.00 ± 0.00	0.00 ± 0.00
	SFL [%]	1.00 ± 0.03	1.27 ± 0.16	1.08 ± 0.03	1.08 ± 0.07
	**SFL_{corr} [%]**	**0.00 ± 0.00**	**0.17 ± 0.02**	**0.00 ± 0.00**	**0.00 ± 0.00**

Table S3: Relative mass isotopomer fractions of amino acids from hydrolyzed cell protein of *A. gossypii* B2 grown on naturally labeled vegetable oil and 99 % [$U^{13}C$] yeast extract, [$^{13}C_5$] glutamate, or the combined addition of [$^{13}C_2$] glycine, [^{13}C] formate, [$^{13}C_5$] glutamate, and [$U^{13}C$] yeast extract. Cultivation of *A. gossypii* on naturally labeled medium served as control. Data denote corrected labeling patterns. Data were corrected for fraction of unlabeled biomass in the inoculum as well as occurrence of natural isotopes. The mass isotopomer M+0 represents the relative amount of non-labeled, M+1 the amount of singly-labeled mass isotopomer fraction and so on. Data were obtained from three individual replicates.

**Analyte**		**Control**	**[$U^{13}C$] YE**	**[$^{13}C_5$] Glu**	**[$U^{13}C$] Gly, For, Glu, YE**
Ala_260	M+0	0.97 ± 0.00	0.31 ± 0.02	0.94 ± 0.05	0.22 ± 0.03
	M+1	0.03 ± 0.00	0.05 ± 0.00	0.05 ± 0.00	0.04 ± 0.00
	M+2	0.00 ± 0.00	0.04 ± 0.00	0.01 ± 0.00	0.04 ± 0.00
	M+3	0.00 ± 0.00	0.61 ± 0.06	0.01 ± 0.00	0.7 ± 0.08
	SFL [%]	1.13 ± 0.05	65.06 ± 4.01	2.71 ± 0.17	73.96 ± 3.04
	**SFL_{corr} [%]**	**0.03 ± 0.00**	**71.89 ± 4.43**	**1.69 ± 0.04**	**81.89 ± 3.37**
Gly_246	M+0	0.98 ± 0.00	0.86 ± 0.09	0.98 ± 0.00	0.01 ± 0.00
	M+1	0.02 ± 0.00	0.02 ± 0.00	0.02 ± 0.00	0.02 ± 0.00
	M+2	0.00 ± 0.00	0.12 ± 0.01	0.00 ± 0.00	0.97 ± 0.00
	SFL [%]	1.11 ± 0.04	12.95 ± 1.25	1.23 ± 0.05	97.89 ± 2.64
	**SFL_{corr} [%]**	**0.01 ± 0.00**	**12.20 ± 1.18**	**0.13 ± 0.01**	**99.61 ± 2.69**
Val_288	M+0	0.95 ± 0.00	0.07 ± 0.00	0.95 ± 0.00	0.07 ± 0.00
	M+1	0.05 ± 0.00	0.01 ± 0.00	0.05 ± 0.00	0.01 ± 0.00
	M+2	0.00 ± 0.00	0.01 ± 0.00	0.00 ± 0.00	0.01 ± 0.00
	M+3	0.00 ± 0.00	0.01 ± 0.00	0.00 ± 0.00	0.01 ± 0.00
	M+4	0.00 ± 0.00	0.05 ± 0.01	0.00 ± 0.00	0.05 ± 0.00
	M+5	0.00 ± 0.00	0.86 ± 0.8	0.00 ± 0.00	0.86 ± 0.9
	SFL [%]	1.09 ± 0.03	91.05 ± 7.50	1.15 ± 0.02	90.89 ± 9.01
	**SFL_{corr} [%]**	**0.00 ± 0.00**	**103.92 ± 8.56**	**0.05 ± 0.01**	**103.74 ± 10.28**
Leu_274	M+0	0.95 ± 0.00	0.06 ± 0.00	0.95 ± 0.00	0.07 ± 0.00
	M+1	0.05 ± 0.00	0.00 ± 0.00	0.05 ± 0.00	0.00 ± 0.00
	M+2	0.00 ± 0.00	0.00 ± 0.00	0.00 ± 0.00	0.00 ± 0.00
	M+3	0.00 ± 0.00	0.00 ± 0.00	0.00 ± 0.00	0.00 ± 0.00
	M+4	0.00 ± 0.00	0.05 ± 0.00	0.00 ± 0.00	0.05 ± 0.00
	M+5	0.00 ± 0.00	0.88 ± 0.10	0.00 ± 0.00	0.87 ± 0.08
	SFL [%]	1.09 ± 0.01	92.16 ± 4.36	1.11 ± 0.02	91.42 ± 5.47
	**SFL_{corr} [%]**	**0.00 ± 0.00**	**104.33 ± 4.94**	**0.05 ± 0.01**	**103.48 ± 6.19**

Table S3: Relative mass isotopomer fractions of amino acids from hydrolyzed cell protein of *A. gossypii* B2 grown on naturally labeled vegetable oil and 99 % [U^{13}C] yeast extract, [$^{13}C_5$] glutamate, or the combined addition of [$^{13}C_2$] glycine, [^{13}C] formate, [$^{13}C_5$] glutamate, and [U^{13}C] yeast extract **(continued)**.

**Analyte**		**Control**	**[U^{13}C] YE**	**[$^{13}C_5$] Glu**	**[U^{13}C] Gly, For, Glu, YE**
Ile_274	M+0	0.94 ± 0.00	0.09 ± 0.01	0.93 ± 0.07	0.09 ± 0.02
	M+1	0.06 ± 0.00	0.01 ± 0.00	0.06 ± 0.00	0.01 ± 0.00
	M+2	0.00 ± 0.00	0.04 ± 0.00	0.00 ± 0.00	0.04 ± 0.00
	M+3	0.00 ± 0.00	0.01 ± 0.00	0.00 ± 0.00	0.01 ± 0.00
	M+4	0.00 ± 0.00	0.05 ± 0.00	0.00 ± 0.00	0.05 ± 0.00
	M+5	0.00 ± 0.00	0.8 ± 0.07	0.00 ± 0.00	0.79 ± 0.08
	SFL [%]	1.24 ± 0.12	86.35 ± 6.53	1.39 ± 0.21	86.08 ± 10.11
	**SFL_{corr} [%]**	**0.14 ± 0.01**	**98.87 ± 7.48**	**0.10 ± 0.02**	**98.56 ± 11.58**
Pro_258	M+0	0.95 ± 0.00	n.d.*	0.85 ± 0.09	0.26 ± 0.03
	M+1	0.05 ± 0.00	n.d.	0.08 ± 0.02	0.06 ± 0.00
	M+2	0.00 ± 0.00	n.d.	0.02 ± 0.00	0.03 ± 0.00
	M+3	0.00 ± 0.00	n.d.	0.00 ± 0.00	0.03 ± 0.00
	M+4	0.00 ± 0.00	n.d.	0.04 ± 0.00	0.62 ± 0.05
	SFL [%]	1.17 ± 0.10	n.d.	7.59 ± 0.60	67.25 ± 4.02
	**SFL_{corr} [%]**	**0.07 ± 0.01**	**n.d.**	**6.81 ± 0.54**	**76.00 ± 4.54**
Ser_390	M+0	0.97 ± 0.00	0.58 ± 0.04	0.96 ± 0.02	0.01 ± 0.00
	M+1	0.03 ± 0.00	0.17 ± 0.02	0.04 ± 0.00	0.02 ± 0.00
	M+2	0.00 ± 0.00	0.08 ± 0.00	0.00 ± 0.00	0.11 ± 0.01
	M+3	0.00 ± 0.00	0.17 ± 0.01	0.00 ± 0.00	0.87 ± 0.09
	SFL [%]	1.08 ± 0.05	28.01 ± 1.32	1.33 ± 0.11	94.47 ± 6.78
	**SFL_{corr} [%]**	**0.00 ± 0.00**	**31.52 ± 1.49**	**0.24 ± 0.02**	**109.38 ± 7.85**
Thr_404	M+0	0.96 ± 0.00	0.16 ± 0.02	0.94 ± 0.03	0.13 ± 0.02
	M+1	0.04 ± 0.00	0.02 ± 0.00	0.05 ± 0.00	0.02 ± 0.00
	M+2	0.00 ± 0.00	0.01 ± 0.00	0.01 ± 0.00	0.01 ± 0.00
	M+3	0.00 ± 0.00	0.04 ± 0.00	0.00 ± 0.00	0.04 ± 0.00
	M+4	0.00 ± 0.00	0.77 ± 0.06	0.01 ± 0.00	0.79 ± 0.08
	SFL [%]	1.09 ± 0.03	81.01 ± 7.98	2.26 ± 0.35	83.14 ± 4.41
	**SFL_{corr} [%]**	**0.00 ± 0.00**	**87.60 ± 8.63**	**1.22 ± 0.19**	**89.93 ± 4.77**

* n.d. = not determined

Table S3: Relative mass isotopomer fractions of amino acids from hydrolyzed cell protein of *A. gossypii* B2 grown on naturally labeled vegetable oil and 99 % [U^{13}C] yeast extract, [$^{13}C_5$] glutamate, or the combined addition of [$^{13}C_2$] glycine, [^{13}C] formate, [$^{13}C_5$] glutamate, and [U^{13}C] yeast extract **(continued)**.

**Analyte**		**Control**	**[U^{13}C] YE**	**[$^{13}C_5$] Glu**	**[U^{13}C] Gly, For, Glu, YE**
Phe_336	M+0	0.91 ± 0.00	0.06 ± 0.00	0.91 ± 0.00	0.06 ± 0.00
	M+1	0.08 ± 0.00	0.01 ± 0.00	0.09 ± 0.00	0.01 ± 0.00
	M+2	0.00 ± 0.00	0.00 ± 0.00	0.00 ± 0.00	0.00 ± 0.00
	M+3	0.00 ± 0.00	0.00 ± 0.00	0.00 ± 0.00	0.00 ± 0.00
	M+4	0.00 ± 0.00	0.00 ± 0.00	0.00 ± 0.00	0.00 ± 0.00
	M+5	0.00 ± 0.00	0.00 ± 0.00	0.00 ± 0.00	0.00 ± 0.00
	M+6	0.00 ± 0.00	0.00 ± 0.00	0.00 ± 0.00	0.00 ± 0.00
	M+7	0.00 ± 0.00	0.01 ± 0.00	0.00 ± 0.00	0.01 ± 0.00
	M+8	0.00 ± 0.00	0.08 ± 0.00	0.00 ± 0.00	0.08 ± 0.00
	M+9	0.00 ± 0.00	0.85 ± 0.09	0.00 ± 0.00	0.84 ± 0.08
	SFL [%]	1.14 ± 0.07	92.29 ± 6.52	1.05 ± 0.02	91.9 ± 8.71
	**SFL_{corr} [%]**	**0.04 ± 0.00**	**104.11 ± 6.99**	**0.00 ± 0.00**	**103.67 ± 9.83**
Asp_418	M+0	0.96 ± 0.00	0.56 ± 0.05	0.87 ± 0.08	0.49 ± 0.06
	M+1	0.04 ± 0.00	0.07 ± 0.00	0.07 ± 0.00	0.10 ± 0.01
	M+2	0.00 ± 0.00	0.04 ± 0.00	0.03 ± 0.00	0.06 ± 0.00
	M+3	0.00 ± 0.00	0.02 ± 0.00	0.00 ± 0.00	0.03 ± 0.00
	M+4	0.00 ± 0.00	0.3 ± 0.02	0.02 ± 0.00	0.32 ± 0.04
	SFL [%]	1.00 ± 0.08	35.62 ± 2.44	5.6 ± 0.52	39.72 ± 2.02
	**SFL_{corr} [%]**	**0.00 ± 0.00**	**43.11 ± 2.95**	**4.71 ± 0.44**	**48.23 ± 2.45**
Glu_432	M+0	0.95 ± 0.00	0.73 ± 0.07	0.78 ± 0.09	0.57 ± 0.07
	M+1	0.05 ± 0.00	0.10 ± 0.01	0.09 ± 0.02	0.13 ± 0.02
	M+2	0.00 ± 0.00	0.05 ± 0.00	0.02 ± 0.00	0.06 ± 0.00
	M+3	0.00 ± 0.00	0.03 ± 0.00	0.03 ± 0.00	0.05 ± 0.00
	M+4	0.00 ± 0.00	0.01 ± 0.00	0.00 ± 0.00	0.01 ± 0.00
	M+5	0.00 ± 0.00	0.09 ± 0.01	0.08 ± 0.00	0.17 ± 0.01
	SFL [%]	1.03 ± 0.04	14.78 ± 1.12	12.32 ± 1.31	26.11 ± 1.03
	**SFL_{corr} [%]**	**0.00 ± 0.00**	**14.33 ± 1.09**	**11.76 ± 1.25**	**26.21 ± 1.03**
Lys_431	M+0	0.94 ± 0.00	0.07 ± 0.00	0.94 ± 0.00	0.07 ± 0.00
	M+1	0.05 ± 0.00	0.00 ± 0.00	0.06 ± 0.01	0.00 ± 0.00
	M+2	0.00 ± 0.00	0.00 ± 0.00	0.00 ± 0.00	0.00 ± 0.00
	M+3	0.00 ± 0.00	0.00 ± 0.00	0.00 ± 0.00	0.00 ± 0.00
	M+4	0.00 ± 0.00	0.04 ± 0.00	0.00 ± 0.00	0.02 ± 0.00
	M+5	0.00 ± 0.00	0.07 ± 0.00	0.00 ± 0.00	0.06 ± 0.00
	M+6	0.00 ± 0.00	0.81 ± 0.09	0.00 ± 0.00	0.84 ± 0.09
	SFL [%]	0.98 ± 0.12	89.61 ± 9.01	0.94 ± 0.20	90.68 ± 8.69
	**SFL_{corr} [%]**	**0.00 ± 0.00**	**110.19 ± 11.08**	**0.00 ± 0.00**	**111.51 ± 10.69**

Table S3: Relative mass isotopomer fractions of amino acids from hydrolyzed cell protein of *A. gossypii* B2 grown on naturally labeled vegetable oil and 99 % [$U^{13}C$] yeast extract, [$^{13}C_5$] glutamate, or the combined addition of [$^{13}C_2$] glycine, [^{13}C] formate, [$^{13}C_5$] glutamate, and [$U^{13}C$] yeast extract **(continued)**.

**Analyte**		**Control**	**[$U^{13}C$] YE**	**[$^{13}C_5$] Glu**	**[$U^{13}C$] Gly, For, Glu, YE**
Arg_442	M+0	0.94 ± 0.00	0.14 ± 0.01	0.85 ± 0.08	0.08 ± 0.00
	M+1	0.05 ± 0.00	0.03 ± 0.00	0.09 ± 0.01	0.03 ± 0.00
	M+2	0.00 ± 0.00	0.01 ± 0.00	0.01 ± 0.00	0.01 ± 0.00
	M+3	0.00 ± 0.00	0.00 ± 0.00	0.01 ± 0.00	0.01 ± 0.00
	M+4	0.00 ± 0.00	0.01 ± 0.00	0.00 ± 0.00	0.01 ± 0.00
	M+5	0.00 ± 0.00	0.08 ± 0.00	0.02 ± 0.00	0.08 ± 0.01
	M+6	0.00 ± 0.00	0.73 ± 0.08	0.00 ± 0.00	0.79 ± 0.08
	SFL [%]	1.10 ± 0.04	80.83 ± 4.74	5.04 ± 0.67	87.24 ± 10.41
	**SFL_{corr} [%]**	**0.00 ± 0.00**	**83.87 ± 4.92**	**4.13 ± 0.55**	**90.61 ± 10.81**
Tyr_466	M+0	0.92 ± 0.00	0.07 ± 0.00	0.8 ± 0.09	0.06 ± 0.00
	M+1	0.08 ± 0.00	0.02 ± 0.00	0.13 ± 0.03	0.02 ± 0.00
	M+2	0.00 ± 0.00	0.01 ± 0.00	0.04 ± 0.00	0.01 ± 0.00
	M+3	0.00 ± 0.00	0.01 ± 0.00	0.02 ± 0.00	0.01 ± 0.00
	M+4	0.00 ± 0.00	0.00 ± 0.00	0.01 ± 0.00	0.00 ± 0.00
	M+5	0.00 ± 0.00	0.00 ± 0.00	0.00 ± 0.00	0.00 ± 0.00
	M+6	0.00 ± 0.00	0.00 ± 0.00	0.00 ± 0.00	0.00 ± 0.00
	M+7	0.00 ± 0.00	0.01 ± 0.00	0.00 ± 0.00	0.01 ± 0.00
	M+8	0.00 ± 0.00	0.08 ± 0.01	0.00 ± 0.00	0.07 ± 0.00
	M+9	0.00 ± 0.00	0.80 ± 0.07	0.00 ± 0.00	0.81 ± 0.09
	SFL [%]	1.14 ± 0.05	88.17 ± 6.35	3.45 ± 0.31	89.8 ± 11.04
	**SFL_{corr} [%]**	**0.04 ± 0.00**	**92.08 ± 6.63**	**2.47 ± 0.22**	**93.80 ± 11.53**
His_440	M+0	0.93 ± 0.00	0.07 ± 0.00	0.93 ± 0.00	n.d.*
	M+1	0.06 ± 0.00	0.05 ± 0.00	0.06 ± 0.00	n.d.
	M+2	0.00 ± 0.00	0.01 ± 0.00	0.00 ± 0.00	n.d.
	M+3	0.00 ± 0.00	0.01 ± 0.00	0.01 ± 0.00	n.d.
	M+4	0.00 ± 0.00	0.01 ± 0.00	0.00 ± 0.00	n.d.
	M+5	0.00 ± 0.00	0.06 ± 0.02	0.00 ± 0.00	n.d.
	M+6	0.00 ± 0.00	0.80 ± 0.10	0.00 ± 0.00	n.d.
	SFL [%]	1.21 ± 0.13	86.75 ± 4.01	1.43 ± 0.35	n.d.
	**SFL_{corr} [%]**	**0.11 ± 0.01**	**103.64 ± 4.79**	**0.34 ± 0.08**	**n.d.**

* n.d. = not determined

Table S3: Relative mass isotopomer fractions of amino acids from hydrolyzed cell protein of *A. gossypii* B2 grown on naturally labeled vegetable oil and 99 % [U^{13}C] yeast extract, [$^{13}C_5$] glutamate, or the combined addition of [$^{13}C_2$] glycine, [^{13}C] formate, [$^{13}C_5$] glutamate, and [U^{13}C] yeast extract **(continued)**.

**Analyte**		**Control**	**[U^{13}C] YE**	**[$^{13}C_5$] Glu**	**[U^{13}C] Gly, For, Glu, YE**
Glc_554	M+0	0.94 ± 0.00	0.73 ± 0.07	0.74 ± 0.08	0.55 ± 0.06
	M+1	0.05 ± 0.00	0.17 ± 0.02	0.16 ± 0.02	0.23 ± 0.02
	M+2	0.00 ± 0.00	0.05 ± 0.00	0.05 ± 0.00	0.11 ± 0.01
	M+3	0.00 ± 0.00	0.04 ± 0.00	0.04 ± 0.00	0.09 ± 0.01
	M+4	0.00 ± 0.00	0.01 ± 0.00	0.00 ± 0.00	0.02 ± 0.00
	M+5	0.00 ± 0.00	0.00 ± 0.00	0.00 ± 0.00	0.01 ± 0.00
	M+6	0.00 ± 0.00	0.00 ± 0.00	0.00 ± 0.00	0.00 ± 0.00
	SFL [%]	1.14 ± 0.10	7.24 ± 0.50	7.12 ± 0.39	13.78 ± 1.52
	**SFL_{corr} [%]**	**0.04 ± 0.00**	**6.20 ± 0.43**	**6.30 ± 0.35**	**12.81 ± 1.41**

Biomass composition of *A. gossypii* for flux calculations

Table S4: Anabolic precursor demand of *A. gossypii* derived from the genome-scale metabolic model [5], adjusted for the content and composition of lipids for growth on vegetable oil [6] and underlying pathway stoichiometry for riboflavin production [7-10]. 3PG, 3-phosphoglycerate; AcCoA, acetyl-CoA; AKG, α-ketoglutarate; G3P, glyceraldehyde 3-phosphate; G6P, glucose 6-phosphate; E4P, erythrose 4-phosphate; F6P, fructose 6-phosphate; OAA, oxaloacetate; PEP, phosphoenolpyruvate; PYR, pyruvate; R5P, ribose 5-phosphate.

Precursor	Demand [µmol g^{-1}]	G6P	F6P	R5P	E4P	G3P	3PG	PEP	PYR	AcCoA	OAA	AKG	NADPH
Ala	357.3								1				1
Arg	135.8											1	4
Asn	171.5										1		1
Asp	171.5										1		1
Cys	42.9						1						5
Gln	268.0											1	1
Glu	268.0											1	1
Gly	325.2						1						1
His	75.0			1									1
Ile	171.5								1		1		5
Leu	250.1								2	1			2
Lys	239.4								1		1		3
Met	50.0										1		8
Phe	114.3				1			2					2
Pro	128.6											1	3
Ser	253.7						1						1
Thr	196.5										1		3
Trp	28.0			1	1			1					2
Tyr	96.5				1			2					2
Val	257.3								2				2
**Protein**		**0**	**0**	**103**	**239**	**0**	**622**	**450**	**1783**	**250**	**1001**	**800**	**7092**
ATP	51.0			1			1						1
GTP	20.0			1			1						
CTP	51.0			1							1		1
UTP	67.0			1							1		1
**RNA**		**0**	**0**	**189**	**0**	**0**	**71**	**0**	**0**	**0**	**118**	**0**	**169**
dATP	3.6			1			1						2
dGTP	2.4			1			1						1
dCTP	2.4			1							1		2
dTTP	3.6			1							1		3
**DNA**		**0**	**0**	**12**	**0**	**0**	**6**	**0**	**0**	**0**	**6**	**0**	**25**
Lipid	240.4					1				26			3
Glycogen	581.5	1											
Mannan	821.0		1										
Trehalose	23.3	1											
Riboflavin	8.6			3			1						1
**Total**		**605**	**821**	**330**	**239**	**240**	**707**	**450**	**1783**	**6573**	**1125**	**800**	**10660**

Table S5: ***(For Table see next page)*** Contribution of nutrient uptake from the medium and *de novo* synthesis of precursors to the supply of cellular building blocks for *A. gossypii*. Values for the precursor demand were taken from Ledesma-Amaro et al. [5] and adjusted for growth on vegetable oil based on Stahmann et al. [6]. Correlation of the demand values with experimental summed fractional labeling (SFL) data from combined results of parallel ^{13}C isotope studies (**Table S2** and **Table S3**) yielded the *de novo* precursor demand for growth on the studied growth conditions: complex medium and rapeseed oil. The full length bar indicates the total precursor demand: the purple fraction depicts the measured percentage taken up from the medium, while the grey fraction depicts the resulting *de novo* biosynthetic fraction of the precursor. Since the ^{13}C labeling could not be measured for all metabolites, following assumptions were made. For the amino acids cysteine, methionine, tryptophan 100 % uptake were defined, since the measurable and measured amino acids with a complex biosynthesis were generally taken up from the medium (e.g. phenylalanine, isoleucine). Since the purine and pyrimidine biosyntheses are feedback regulated pathways [11-13] and the presence of nucleotides in yeast extract has been reported [14] and could also be measured in riboflavin samples even at a late stage of cultivation (after 144 h), uptake of DNA and RNA building blocks as well as the GTP-precursor for riboflavin (**Fig. 9**) was defined as 100 %. For trehalose the same ratio was assumed as for the glycogen pool, since the two building blocks share the same precursor glucose 6-phosphate. For the flux calculations lipids were considered to be completely *de novo* synthesized, however the more likely scenario is that fatty acids are taken up by the cell and re-esterified with glycerol inside the cell. Stahmann et al. [6] reported that the lipid composition of the substrate oil resembled the lipid composition of storage triacylglycerides in the *A. gossypii* cells. Due to that, the acetyl-CoA *de novo* demand is not specified in this table. ATP, adenosine triphosphate; CTP, cytidine triphosphate; dATP, deoxyadenosine triphosphate; dCTP, deoxycytidine triphosphate; dGTP, deoxyguanosine triphosphate; dTTP, deoxythymidine triphosphate; GTP, guanosine triphosphate; UTP, uridine triphosphate.

Precursor	Total demand [µmol g^{-1}]	Uptake [%]	*De novo* biosynthesis [%]	Resulting *de novo* demand [µmol g^{-1}]
Alanine	357.3	80 ± 5	20 ± 5	71.5 ± 15.4
Arginine	135.8	90 ± 4	10 ± 4	13.6 ± 5.4
Asparagine	171.5	89 ± 9	11 ± 9	18.9 ± 14.3
Aspartate	171.5	48 ± 4	52 ± 4	89.4 ± 5.2
Cysteine‡	42.9	100	0	0.0
Glutamine	268.0	74 ± 4	26 ± 4	69.7 ± 9.4
Glutamate	268.0	27 ± 2	74 ± 2	197.0 ± 4.0
Glycine	325.2	98 ± 1	2 ± 1	6.5 ± 30.5
Histidine	75.0	100 ± 5	0 ± 5	0.0 ± 0.0
Isoleucine	171.5	100 ± 7	0 ± 7	0.0 ± 0.0
Leucine	250.1	100 ± 4	0 ± 4	0.0 ± 0.0
Lysine	239.4	100 ± 11	0 ± 11	0.0 ± 0.1
Methionine‡	50.0	100	0	0.0
Phenylalanine	114.3	100 ± 7	0	0.0 ± 0.0
Proline	128.6	74 ± 4	26 ± 4	33.4 ± 4.8
Serine	253.7	88 ± 3	12 ± 3	30.4 ± 8.0
Threonine	196.5	89 ± 9	11 ± 9	21.6 ± 17.5
Tryptophan‡	28.0	100	0	0.0
Tyrosine	96.5	95 ± 7	5 ± 7	4.8 ± 6.7
Valine	257.3	100 ± 9	0 ± 9	0.0 ± 0.0
ATP‡	51.0	100	0	0.0
GTP‡	20.0	100	0	0.0
CTP‡	51.0	100	0	0.0
UTP‡	67.0	100	0	0.0
dATP‡	3.6	100	0	0.0
dGTP‡	2.4	100	0	0.0
dCTP‡	2.4	100	0	0.0
dTTP‡	3.6	100	0	0.0
Lipid	240.4	n.d.	n.d.	n.d.
Glycogen	581.5	13 ± 0	88 ± 0	508.8 ± 2.6
Mannan‡	821.0	0	100	821.0
Trehalose	23.3	13 ± 0	88 ± 0	20.4 ± 0.0
Riboflavin‡	8.6	53	47	4.0

‡ The demand for the precursor is assumed. Therefore, no standard deviation could be calculated for the according values.

Determination of measured fluxes and metabolite balances for flux calculations during growth of *A. gossypii*

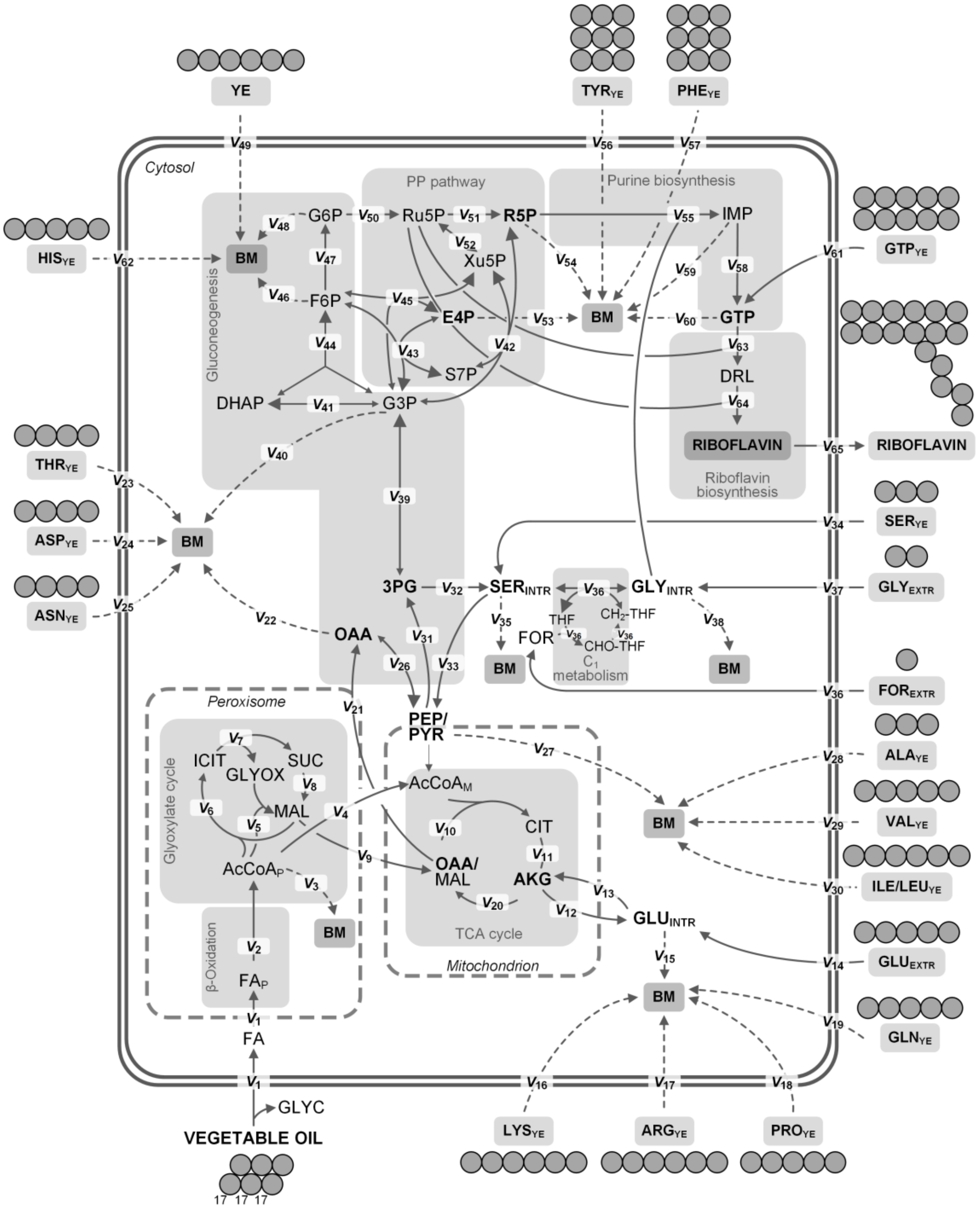

Figure S2: Metabolic network of *A. gossypii*, including extracellular reactions, reactions between intermediary metabolite pools, and anabolic reactions. The reactions numbers refer to the formulation of metabolite balances (see below). The direction of net reactions is indicated by size of arrow head. For abbreviations see **Fig. 3**.

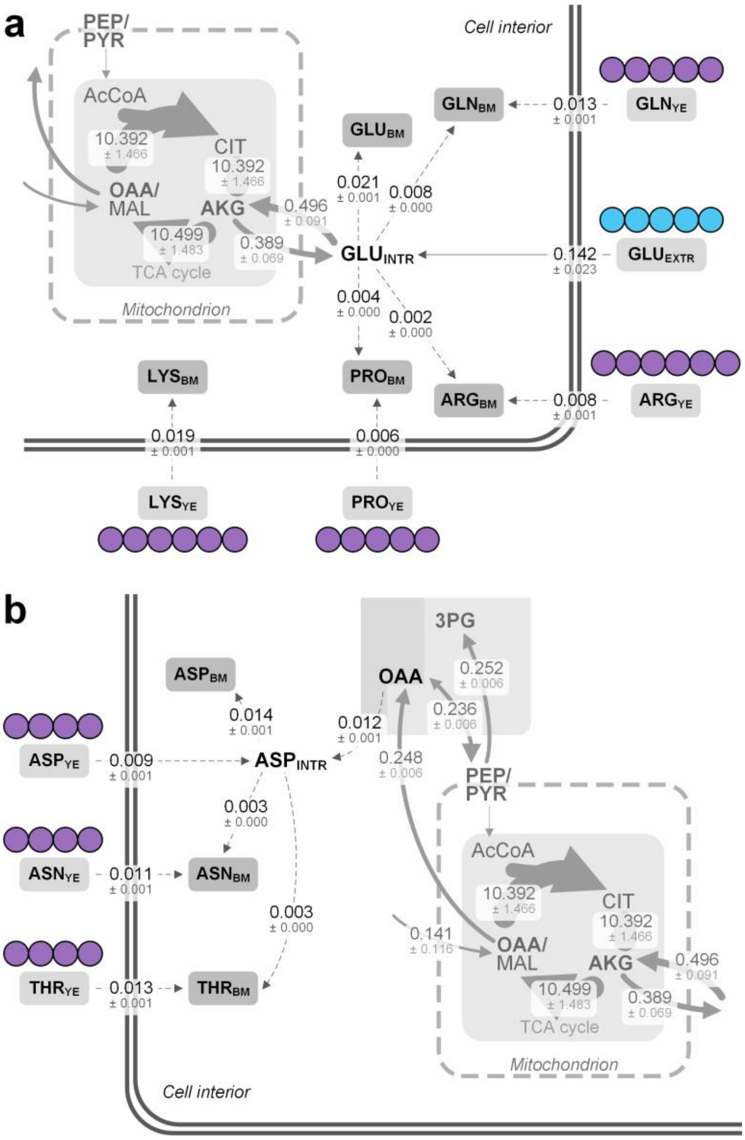

Figure S3: Detailed view of carbon fluxes into biomass for amino acids stemming from glutamate (a) or aspartate (b) during growth of *A. gossypii* B2 on vegetable oil and complex medium. Fluxes are given in mmol g_{CDW}^{-1} h^{-1} and are normalized to the substrate uptake rate (0.43 mmol g^{-1} h^{-1}). Flux calculations were derived from parallel ^{13}C tracer studies with [$^{13}C_2$] glycine, [^{13}C] formate, [$^{13}C_5$] glutamate, and [$U^{13}C$] yeast extract (**Table S2**, **Table S3**). The arrow thickness is proportional to the corresponding flux. The direction of net fluxes is indicated by size of arrow head. Dashed arrows represent fluxes into biomass. Reactions at the OAA/MAL and PEP/PYR node could not be resolved by this approach and represent lumped fluxes. The rate of the pyruvate dehydrogenase could not be determined. The contribution of intracellular glutamate or aspartate to glutamine and asparagine, respectively, could not be measured and was estimated based on the ^{13}C labeling data for proline and threonine. The full figure is presented in **Fig. 5**. 3PG, 3-phosphoplycerate; AcCoA, acetyl-CoA; AKG, α-ketoglutarate; ALA, alanine; ARG, arginine; ASN, asparagine; ASP, aspartate; BM, biomass; CIT, citrate; GLN, glutamine; GLU, glutamate; MAL, malate; OAA, oxaloacetate; PEP, phosphoenolpyruvate; PRO, proline; PYR, pyruvate; THR, threonine; YE, yeast extract.

Data from ^{13}C NMR analyses

Table S6: Relative ^{13}C enrichment of all seventeen carbon atoms of riboflavin produced by *A. gossypii* B2 from different ^{13}C-labeled tracer substrates. The labeling was analyzed by ^{13}C NMR. The time refers to the time point of respective tracer addition. For, formate; Glu, glutamate; Gly, Glycine; Ser, serine; YE, yeast extract. Data denote mean values for three independent replicates with a mean standard deviation of 5 %. Data for [^{13}C] formate, [$^{13}C_2$] glycine, and [3-^{13}C] serine were obtained in our previous study [1].

**C-atom**	**Chemical shift [ppm]**	**Relative enrichment [%]**						
		**Nat. lab. precursors**	**[^{13}C] For**		**[$^{13}C_2$] Gly**	**[3-^{13}C] Ser**	**[$U^{13}C$] YE**	**[$^{13}C_5$] Glu**
		0 h	0 h	48 h	0 h	48 h	32 h	0 h
2	155.5	1.1	**4.7**	**12.3**	1.1	**5.9**	**15.4**	**2.1**
4	159.9	1.1	1.1	1.1	1.0	1.0	**11.8**	**2.8**
4a	136.8	1.1	1.3	0.9	**71.2**	0.9	**18.4**	1.1
5a	134	1.1	1.1	0.9	1.4	1.0	**4.8**	**2.9**
6	130	1.1	1.1	1.1	1.0	1.1	**5.0**	**3.3**
7	137.1	1.1	0.9	0.7	1.3	0.8	**3.7**	**2.8**
7α	18.8	1.3	1.1	1.2	1.1	1.1	**3.8**	**2.7**
8	146	1.1	1.0	0.9	1.3	0.9	**4.6**	**2.9**
8α	20.8	1.3	1.1	1.2	1.1	1.1	**4.7**	**3.6**
9	117.4	1.0	1.5	1.0	0.9	1.2	**3.9**	**1.8**
9a	132.1	1.1	1.1	0.9	1.6	0.9	**3.7**	**1.4**
10a	150.8	1.1	1.0	0.8	**70.7**	0.9	**18.5**	1.0
1’	47.3	1.2	1.1	1.0	1.0	1.0	**9.9**	**3.9**
2’	68.8	1.1	1.1	1.0	1.2	1.0	**8.8**	**5.0**
3’	73.6	1.2	1.1	0.9	1.5	0.9	**7.9**	**3.9**
4’	72.8	1.3	1.1	1.0	0.8	0.9	**8.3**	**4.8**
5’	63.4	1.0	1.1	1.1	0.8	1.1	**7.5**	**2.7**

Determination of carbon fluxes and carbon balances for carbon flux calculations during the riboflavin biosynthetic phase of *A. gossypii*

The integrated rich data set enabled the calculation of a metabolic flux map, comprising the respective contributions of single medium ingredients to riboflavin in this environment (**Fig. 8**). A specific approach for data processing and carbon balancing was chosen to finally derive the flux distribution. Different from conventional flux studies on only one substrate, with precise molecular fluxes [15, 16], this work had to consider the positional contribution of individual precursors to specific parts or even single carbon atoms of the riboflavin molecule. That is, why the flux estimation here was taken to the level of single carbon atoms, and fluxes were split into fluxes from individual carbon atoms of nutrients, which converged within the metabolism to form molecules or parts of the vitamin. This perfectly matched the type of labeling information, i.e. positional ^{13}C enrichment that was available to great detail from the conducted labeling studies. The positional ^{13}C enrichments of the single tracer experiments (**Table 3**) were integrated with the pathway stoichiometry. In addition, carbon balancing was applied. The assumption of steady-state, required for this approach, appeared justified, considering that the change of intracellular metabolite pools was small compared to fluxes through those pools [1], similar to a previous approach [17]. Together this rendered a flux distribution based on single carbon atoms (**Fig. 8**). Therefore, the flux into a single carbon atom was set to 1. Likewise, the flux into riboflavin equaled 17, since the vitamin contains seventeen carbon atoms and the flux of e.g. GTP, a ten-carbon molecule, toward riboflavin biosynthesis corresponded to the value 10. Accordingly, fluxes between other metabolites were based on the

sum of the fluxes of their carbon atoms and additionally, considering the ^{13}C labeling from the respective ^{13}C tracers. For each carbon atom of riboflavin, the metabolic precursor was determined (**Fig. S6, Fig. S7, Fig. S8**). The way, in which the individual medium ingredients, e.g. yeast extract, would contribute to that metabolic precursor was evaluated and for each case, the entry point into the metabolism was chosen based on literature [18-21] as well as data obtained in this and our previous study (**Table 3**) [1]. As an example, the C_{4a} atom of riboflavin is derived from intracellular glycine (**Fig. S6**) [20]. In the presented set-up, glycine could originate from extracellular glycine, which was supplemented to the medium, glycine from yeast extract, but also serine from yeast extract, adenine/guanine and ATP/GTP from yeast extract or from rapeseed oil, via 3-phosphoglycerate and serine (**Fig. S6**). Thus, the glycine unit of riboflavin could be derived from three different medium compounds, i.e. glycine, vegetable oil, and yeast extract, and four different metabolites, i.e. glycine, serine, adenine/guanine, or ATP/GTP. Adenine/guanine as well as ATP/GTP are treated as one group, respectively, since the data did not allow a distinction between the individual compounds. Analogous considerations were made for every carbon atom and fluxes were calculated accordingly.

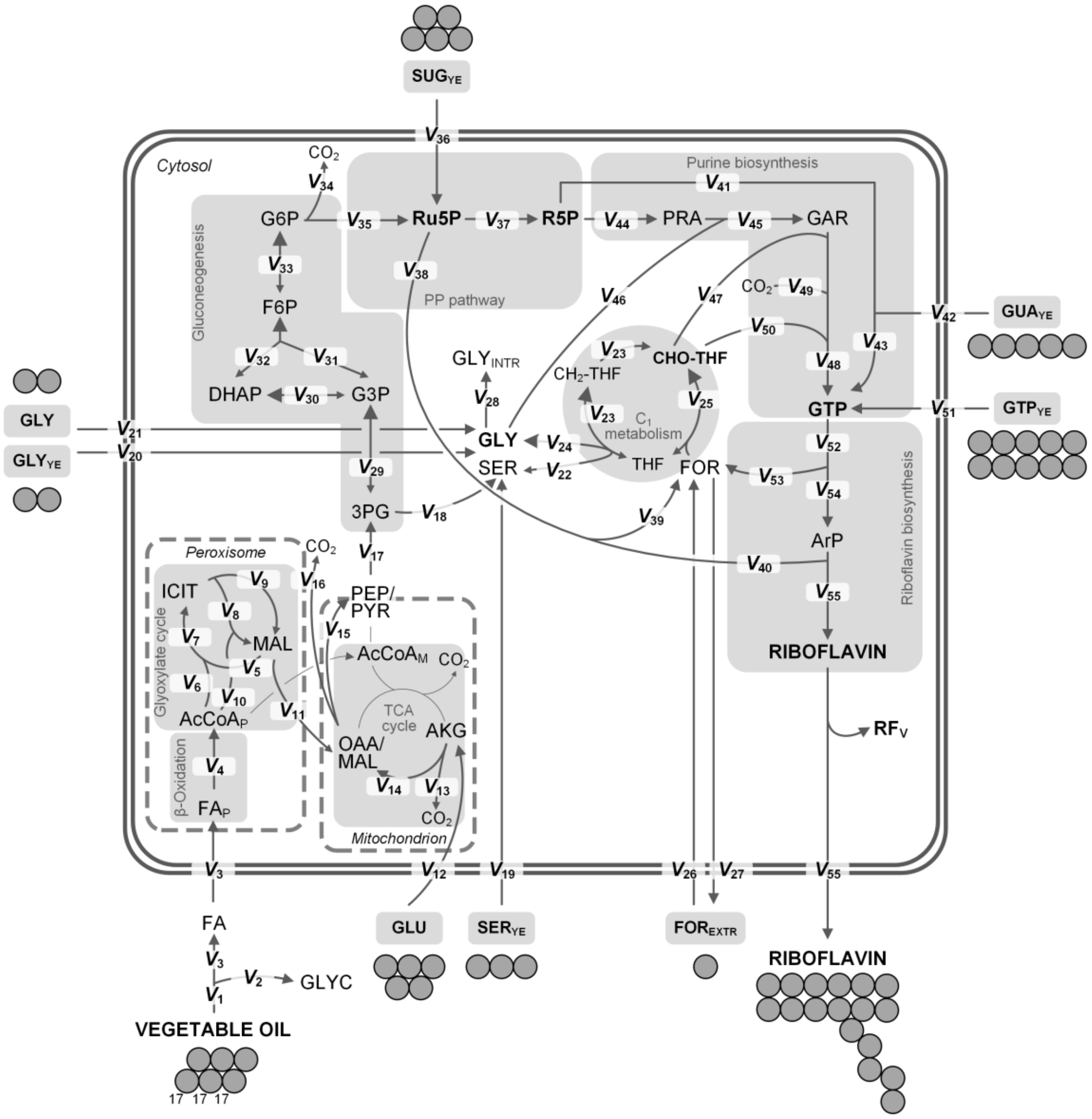

Figure S4: Metabolic network of riboflavin production in *A. gossypii*, including extracellular reactions, reactions between intermediary metabolite pools, and riboflavin biosynthetic reactions. The reaction numbers refer to the formulation of metabolite balances (see below). The direction of net reactions is indicated by size of arrow head. For abbreviations see **Fig. 8**.

The Equations (30) to (36) could be applied in detail to every carbon atom in riboflavin. Carbon atom C_4 of riboflavin will be discussed exemplary in greater detail below, followed by the other carbon atoms of riboflavin, however, to a less extensive extent.

Considerations regarding carbon atom **C_4**:

Equation (S4) was specified for carbon atom C_4 based on Equation (30):

$$v_4 = \sum_{j=1}^{5} v_{4,j} = v_{4,Gly} + v_{4,For} + v_{4,Glu} + v_{4,YE} + v_{4,Oil} = 1 \qquad \text{(S4)}$$

The carbon origin of C_4 of riboflavin is depicted in **Fig. S5**. Since its biosynthetic *de novo* origin is carbon dioxide, the oil and the glutamate fraction of Equation (S4) is defined as fraction derived from carbon dioxide instead, which is why the combined flux equals the flux V_{49} of the network (Equation S5) (**Fig. S4**), i.e. the uptake of single carbon atoms from oil. Carbon dioxide is produced from rapeseed oil (naturally labeled) and glutamate (^{13}C-labeled) through decarboxylation reactions in the TCA cycle as well as at the pyruvate node. Note that the equality in Equation (S5) is straightforward, as only single carbon atoms are considered.

$$v_{4,CO_2} = v_{4,Oil} + v_{4,Glu} = V_{49} \qquad \text{(S5)}$$

For the carbon flux from the yeast extract to C_4, two potential building blocks were considered: GTP (or ATP, however, since this study could not distinguish between the two, only GTP is used) or guanine (or adenine). GTP as well as guanine are part of the yeast extract and are incorporated into riboflavin almost as whole molecule only releasing one carbon atom (**Fig. 8**). Therefore, the flux from the yeast extract could be formulated as follows:

$$v_{4,YE} = v_{4,YE_{GTP}} + v_{4,YE_{GUA}} \quad \text{(S6)}$$

with

YE_{GTP}	GTP (or ATP) originating from ^{13}C-labeled yeast extract
YE_{GUA}	Guanine (or adenine) originating from ^{13}C-labeled yeast extract

GTP shares nine of its ten carbon atoms with riboflavin. The remaining eight carbon atoms comprise the xylene ring and are exclusive carbon atoms for the vitamin. Thus, the carbon flux from GTP from the yeast extract can be expressed as the difference between ^{13}C labeling from [U^{13}C] yeast extract of carbon atoms of the ribityl side chain ($C_{1'}$ to $C_{5'}$) and carbon atoms of the xylene ring (C_{5a} to C_{9a}). In order to simplify the complexity, mean values derived from the structural subunits were used ($\overline{v_{1'-5',YE}}$ and $\overline{v_{5a-9a,YE}}$, respectively).

$$v_{4,YE} = \left(\overline{v_{1'-5',YE}} - \overline{v_{5a-9a,YE}}\right) + v_{4,YE_{GUA}} \quad \text{(S7)}$$

Rearrangement of Equation (S7) leads to the carbon flux from yeast extract-based guanine to carbon atom C_4:

$$v_{4,YE_{GUA}} = v_{4,YE} - \overline{v_{1'-5',YE}} + \overline{v_{5a-9a,YE}} \quad \text{(S8)}$$

Since guanine is incorporated into riboflavin as a whole, it can be assumed that the carbon flux from yeast extract-derived guanine into C_4 also equals the carbon flux into the other three carbon atoms of riboflavin that originate from guanine (C_2, C_{4a}, C_{10a}). This correlation is expressed by Equation (S9).

$$v_{4,YE_{GUA}} = v_{2,YE_{GUA}} = v_{4a,YE_{GUA}} = v_{10a,YE_{GUA}} \quad \text{(S9)}$$

In a likewise manner, this can be applied to the carbon flux from yeast extract-based GTP to carbon atom C_4:

$$v_{4,YE_{GTP}} = v_{i,YE_{GTP}} \quad \text{with } i = 2, 4a, 10a, 1' – 5' \quad \text{(S10)}$$

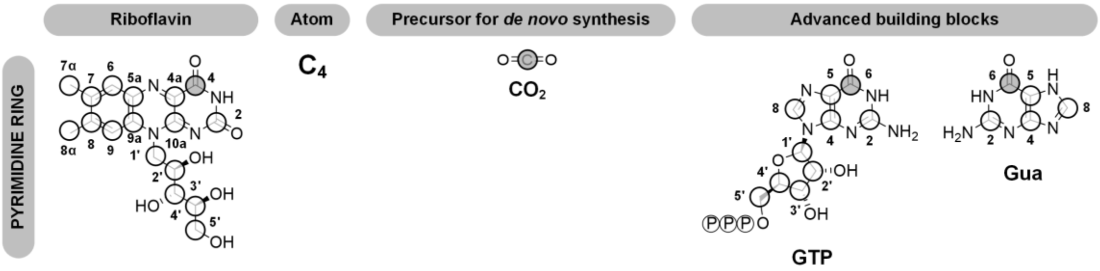

Figure S5: Metabolic origin of carbon atom C_4 of the pyrimidine ring in riboflavin. Grey circles denote the carbon atom of interest and its origin in the metabolic precursor or donor to the precursor. The numbering is specific for the molecule, therefore, e.g. carbon atom 4 of riboflavin does not equal carbon atom 4 of GTP. GTP, guanosine triphosphate; Gua, guanine. This is an excerpt of **Fig. S6**.

Considerations regarding reactions involved in the formation of carbon atom **C_2**:

$$v_2 = \sum_{j=1}^{5} v_{2,j} = 1 \quad \text{(S11)}$$

$$v_{2,YE} = v_{2,YE_{GTP}} + v_{2,YE_{GUA}} + v_{2,YE_{SER}} \quad \text{(S12)}$$

$$v_{2,YE_{SER}} = v_{2,YE} - v_{2,YE_{GTP}} - v_{2,YE_{GUA}} = v_{4a,YE_{SER}} \quad \text{(S13)}$$

$$= v_{10a,YE_{SER}}$$

$$v_{2,Oil} = v_{2,Oil_{Ru5P}} + v_{2,Oil_{SER}} \quad \text{(S14)}$$

$$v_{2,Oil_{SER}} = v_{4a,Oil} \quad \text{(S15)}$$

$$v_{2,Oil_{Ru5P}} = v_{2,Oil} - v_{4a,Oil} \quad \text{(S16)}$$

with

Oil_{Ru5P}	Ru5P originating from vegetable oil
Oil_{SER}	Serine originating from vegetable oil, i.e. 3PG
YE_{SER}	Serine originating from ^{13}C-labeled yeast extract

Considerations regarding reactions involved in the formation of carbon atom **C_{4a}**:

$$v_{4a} = \sum_{j=1}^{5} v_{4a,j} = 1 \quad \text{(S17)}$$

$$v_{4a,YE} = v_{4a,YE_{GTP}} + v_{4a,YE_{GUA}} + v_{4a,YE_{SER}} + v_{4a,YE_{Gly}} \quad \text{(S18)}$$

$$v_{4a,Oil} = v_{4a,Oil_{Ser}} \quad \text{(S19)}$$

with

YE_{GLY}	Glycine originating from ^{13}C-labeled yeast extract

Since the considerations for the formation of carbon atom C_{10a} equal the ones for carbon atom C_{4a}, they will not be described.

Considerations regarding reactions involved in the formation of carbon atom **C_{5a}**:

$$v_{5a} = \sum_{j=1}^{5} v_{5a,j} = 1 \quad \text{(S20)}$$

$$v_{5a,Ru5P} = v_{5a,Oil} + v_{5a,Glu} + v_{5a,YE} = 1 \quad \text{(S21)}$$

$$v_{5a,de\ novo} = v_{5a,Oil} + v_{5a,Glu} \quad \text{(S22)}$$

$$v_{5a,de\ novo} = \frac{1}{2} v_{5a,OAA} = v_{5a,G6P} \quad \text{(S23)}$$

with

$v_{5a,de\ novo}$	C_{5a} atom originating from *de novo* biosynthesis through glyoxylate or TCA cycle and gluconeogenesis
$v_{5a,OAA}$	C_{5a} atom originating from the C_2 atom of oxaloacetate
$v_{5a,G6P}$	C_{5a} atom originating from the C_2 atom of glucose 6-phosphate

Since the considerations for the formation of carbon atom C_5 through C_{9a} occurred in a likewise manner as the ones for carbon atom C_{5a}, they will not be described.

Considerations regarding reactions involved in the formation of carbon atom **$C_{1'}$**:

$$v_{1'} = \sum_{j=1}^{5} v_{1',j} = 1 \quad \text{(S24)}$$

$$v_{1',YE} = v_{1',YE_{SUGAR}} + v_{1',YE_{GTP}} \quad \text{(S25)}$$

$$v_{1',Ru5P} = v_{1',Oil} + v_{1',YE_{SUGAR}} + v_{1',Glu} \quad \text{(S26)}$$

$$v_{1',de\ novo} = v_{1',Oil} + v_{1',Glu} \quad \text{(S27)}$$

$$v_{1',de\ novo} = \frac{1}{2} v_{1',OAA} = v_{1',G6P} \quad \text{(S28)}$$

with

YE_{SUGAR}	Sugar originating from ^{13}C-labeled yeast extract
$v_{1',de\ novo}$	$C_{1'}$ atom originating from *de novo* biosynthesis through glyoxylate or TCA cycle and gluconeogenesis
$v_{1',OAA}$	$C_{1'}$ atom originating from the C_1 atom of oxaloacetate
$v_{1',G6P}$	$C_{1'}$ atom originating from the C_1 atom of glucose 6-phosphate

Since the considerations for the formation of carbon atom $C_{2'}$ through $C_{5'}$ equal the ones for carbon atom $C_{1'}$, they will not be described.

The sums of carbon atoms (Equations 37 to 41) provide three out of 55 pieces of information required for a fully determined metabolic network that consisted of a total of 55 fluxes (V_{55}, V_{40}, V_{54}) (**Fig. S4**). Another piece of information was defined in Equation (S5) (V_{49}). The labeling data for all seventeen carbon atoms of riboflavin from four different labeling experiments (**Table 2** and **Table S6**) and underlying pathway stoichiometry allowed the calculation of 28 fluxes. The calculation of those fluxes is given in Equations (S29) to (S55) (V_2, V_8, V_9, V_{12}, V_{13}, V_{16}, V_{18}, V_{19}, V_{20}, V_{21}, V_{22}, V_{23}, V_{24}, V_{25}, V_{26}, V_{34}, V_{36}, V_{37}, V_{38}, V_{39}, V_{41}, V_{42}, V_{43}, V_{46}, V_{47}, V_{50}, V_{51}, V_{53}). It should be noted that most of the numbers are derived from carbon atom stoichiometry (e.g. 52_{FA} designates that FA contains 52 carbon atoms, if it is defined as three FA chains of average length of 17.3 carbon atoms). In those cases, the origin of those carbon atoms is

specified by the index (for abbreviations refer to **Fig. 8**). Some carbon fluxes are presented as mean carbon flux from a structural group, e.g. xylene ring ($\overline{v_{5a-9a,Glu}}$ and $\overline{v_{5a-9a,YE}}$) or two-carbon unit of riboflavin ($\overline{v_{4a;10a,Gly}}$ and $\overline{v_{4a;10a,YE_{Gly}}}$). This was done in order to simplify the calculations and does not affect their outcome. The carbon fluxes v_{GUA_8} in Equation (S50) and v_{GTP_8} in Equation (S54) refer to the flux of the carbon atom number 8 in guanine or GTP (**Fig. S5**), respectively. Their values equal the ones in Equation (S9) and Equation (S10), respectively.

$$V_2 = V_3 \cdot \frac{3_{GLYC}}{52_{FA}} \tag{S29}$$

$$V_8 = \frac{V_{11}}{2_{GLYOX}} \tag{S30}$$

$$V_9 = V_{11} \tag{S31}$$

$$V_{12} = V_{15} \cdot \frac{\overline{v_{5a-9a,Glu}}}{1 - \overline{v_{5a-9a,YE}}} \cdot \frac{5_{GLU}}{4_{OAA}} \tag{S32}$$

$$V_{13} = \frac{V_{12}}{5_{GLU}} \tag{S33}$$

$$V_{16} = \frac{V_{15}}{4_{OAA}} \tag{S34}$$

$$V_{18} = V_{22} \cdot \frac{v_{2,Oil_{SER}}}{v_{2,YE_{SER}} + v_{2,Oil_{SER}}} \tag{S35}$$

$$V_{19} = V_{22} \cdot \frac{v_{2,YE_{SER}}}{v_{2,YE_{SER}} + v_{2,Oil_{SER}}} \tag{S36}$$

$$V_{20} = (V_{28} + V_{46}) \cdot \frac{\overline{v_{4a;10a,Gly}}}{1 - \overline{v_{4a;10a,YE_{GTP}}} - \overline{v_{4a;10a,YE_{GUA}}}} \tag{S37}$$

$$V_{21} = (V_{28} + V_{46}) \cdot \frac{\overline{v_{4a;10a,YE_{Gly}}}}{1 - \overline{v_{4a;10a,YE_{GTP}}} - \overline{v_{4a;10a,YE_{GUA}}}} \tag{S38}$$

$$V_{22} = 3_{SER} \cdot V_{23} \tag{S39}$$

$$V_{23} = 2 \cdot \left(v_{2,YE_{SER}} + v_{2,Oil_{SER}}\right) \tag{S40}$$

$$V_{24} = 2_{GLY} \cdot V_{23} \tag{S41}$$

$$V_{25} = 2 \cdot \left(v_{2,Oil_{Ru5P}} + v_{2,For}\right) \tag{S42}$$

$$V_{26} = V_{39} \cdot \frac{v_{2,For}}{v_{2,Oil_{Ru5P}}} \tag{S43}$$

$$V_{34} = \frac{V_{33}}{6_{G6P}} \tag{S44}$$

$$V_{36} = \sum_{i=1'}^{5'} v_{i,YE_{SUGAR}} + \sum_{i=5a}^{9a} v_{i,YE} \tag{S45}$$

$$V_{37} = \sum_{i=1'}^{5'} v_{i,Ru5P} \tag{S46}$$

$$V_{38} = V_{39} + V_{40} \tag{S47}$$

$$V_{39} = \frac{V_{40}}{4_{DHBP}} \tag{S48}$$

$$V_{41} = V_{42} \tag{S49}$$

$$\begin{aligned} V_{42} = {} & v_{2,YE_{GUA}} + v_{4,YE_{GUA}} + v_{4a,YE_{GUA}} + v_{10a,YE_{GUA}} \\ & + v_{1',YE_{GUA}} + v_{2',YE_{GUA}} + v_{3',YE_{GUA}} \\ & + v_{4',YE_{GUA}} + v_{5',YE_{GUA}} + v_{GUA_8} \end{aligned} \tag{S50}$$

$$V_{43} = V_{41} + V_{42} \tag{S51}$$

$$\begin{aligned} V_{46} = {} & \left(v_{4a} - v_{4a,YE_{GUA}} - v_{4a,YE_{GTP}}\right) \\ & + \left(v_{10a} - v_{10a,YE_{GUA}} - v_{10a,YE_{GTP}}\right) \end{aligned} \tag{S52}$$

$$V_{47} = V_{50} = v_2 - v_{2,YE_{GUA}} - v_{2,YE_{GTP}} \tag{S53}$$

$$V_{51} = v_{2,YE_{GTP}} + v_{4,YE_{GTP}} + v_{4a,YE_{GTP}} + v_{10a,YE_{GTP}} + v_{1',YE_{GTP}} + v_{2',YE_{GTP}} + v_{3',YE_{GTP}} + v_{4',YE_{GTP}} + v_{5',YE_{GTP}} + v_{GTP_8} \quad \text{(S54)}$$

$$V_{53} = \frac{V_{52}}{10_{GTP}} \quad \text{(S55)}$$

Together with the four fluxes determined above (V_{40}, V_{49}, V_{54}, V_{55}) this rendered 32 fluxes. Thus, at least 23 pieces of information were still required to obtain a fully determined network. Therefore, following balances were formulated for the metabolic network depicted in **Fig. S4**, which represents riboflavin production on vegetable oil and yeast extract. Metabolite balances were expressed using the numbering of the fluxes as presented in **Fig. S4**.

FA	$0 = V_1 - V_2 - V_3$	(S56)
FA_P	$0 = V_3 - V_4$	(S57)
$AcCoA_P$	$0 = V_4 - V_6 - V_{10}$	(S58)
ICIT	$0 = V_7 - V_8 - V_9$	(S59)
MAL	$0 = V_9 + V_{10} + V_8 - V_5 - V_{11}$	(S60)
OAA/MAL	$0 = V_{11} + V_{14} - V_{15} - V_{16}$	(S61)
AKG	$0 = V_{12} - V_{13} - V_{14}$	(S62)
PEP/PYR	$0 = V_{15} - V_{17}$	(S63)
3PG	$0 = V_{17} - V_{18} - V_{29}$	(S64)
G3P	$0 = V_{29} - V_{30} - V_{31}$	(S65)
DHAP	$0 = V_{30} - V_{32}$	(S66)
F6P	$0 = V_{31} + V_{32} - V_{33}$	(S67)

G6P	$0 = V_{33} - V_{34} - V_{35}$	(S68)
Ru5P	$0 = V_{35} + V_{36} - V_{37} - V_{38}$	(S69)
R5P	$0 = V_{37} - V_{41} - V_{44}$	(S70)
PRA	$0 = V_{44} + V_{46} - V_{45}$	(S71)
GAR	$0 = V_{45} + V_{47} + V_{49} + V_{50} - V_{48}$	(S72)
GTP	$0 = V_{43} + V_{48} + V_{51} - V_{52}$	(S73)
ArP	$0 = V_{40} + V_{54} - V_{55}$	(S74)
GLY	$0 = V_{20} + V_{21} + V_{24} - V_{28} - V_{46}$	(S75)
SER	$0 = V_{18} + V_{19} - V_{22}$	(S76)
FOR	$0 = V_{26} + V_{39} + V_{53} - V_{25} - V_{27}$	(S77)
CHO-THF	$0 = V_{23} + V_{25} - V_{47} - V_{50}$	(S78)

The rank of the stoichiometric matrix formulated for Equations (S56) to (S78) was 23. This indicated that the 23 metabolite balances were linearly independent. Since the complete network comprised a total of 55 fluxes the combined 55 pieces of information obtained through metabolite balancing and labeling information rendered a fully determined network for flux calculations.

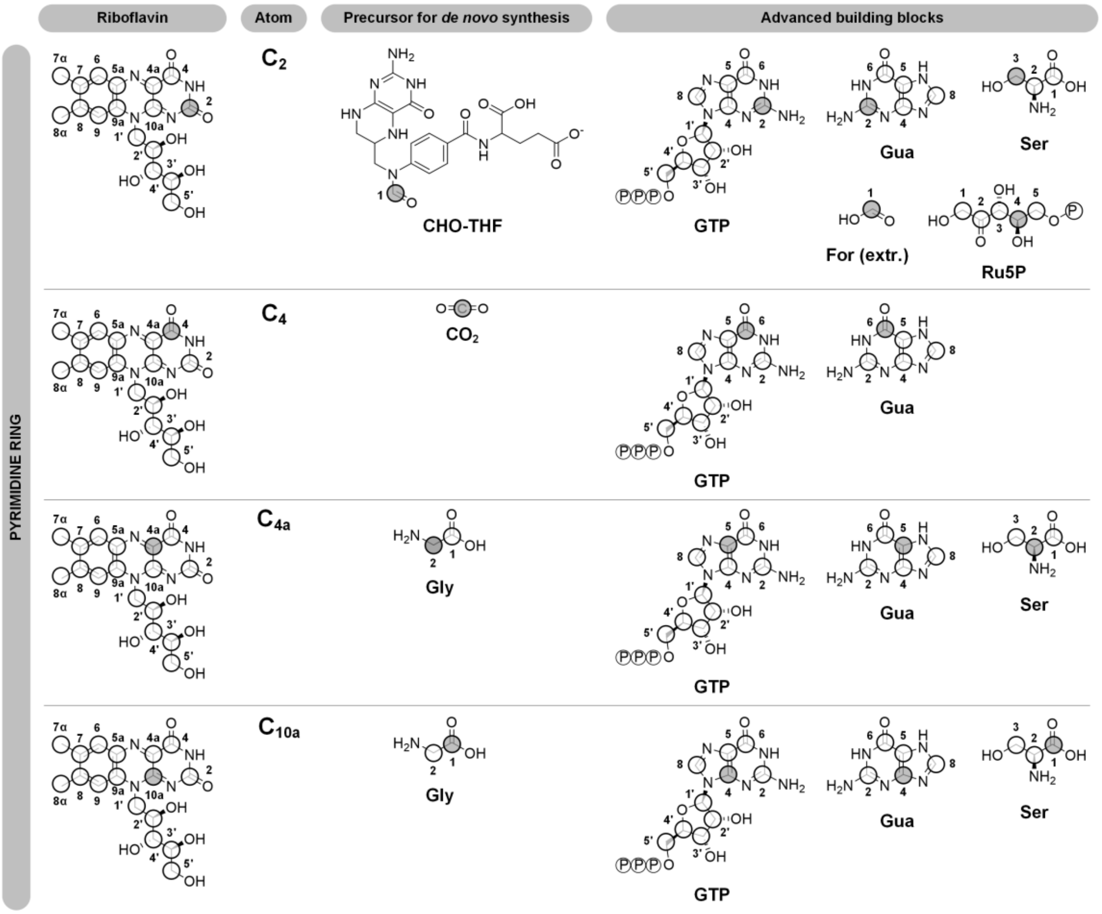

Figure S6: Metabolic origin of carbon atoms of the pyrimidine ring in riboflavin. Grey circles denote the carbon atom of interest and its origin in the metabolic precursor or donor to the precursor. The numbering is specific for the molecule, therefore, e.g. carbon atom 2 of riboflavin does not equal carbon atom 2 of GTP. CHO-THF, 10-formyltetrahydrofolate; For (extr.), formate (extracellular); Gly, glycine; GTP, guanosine triphosphate; Gua, guanine; Ru5P, ribulose 5-phosphate; Ser, serine.

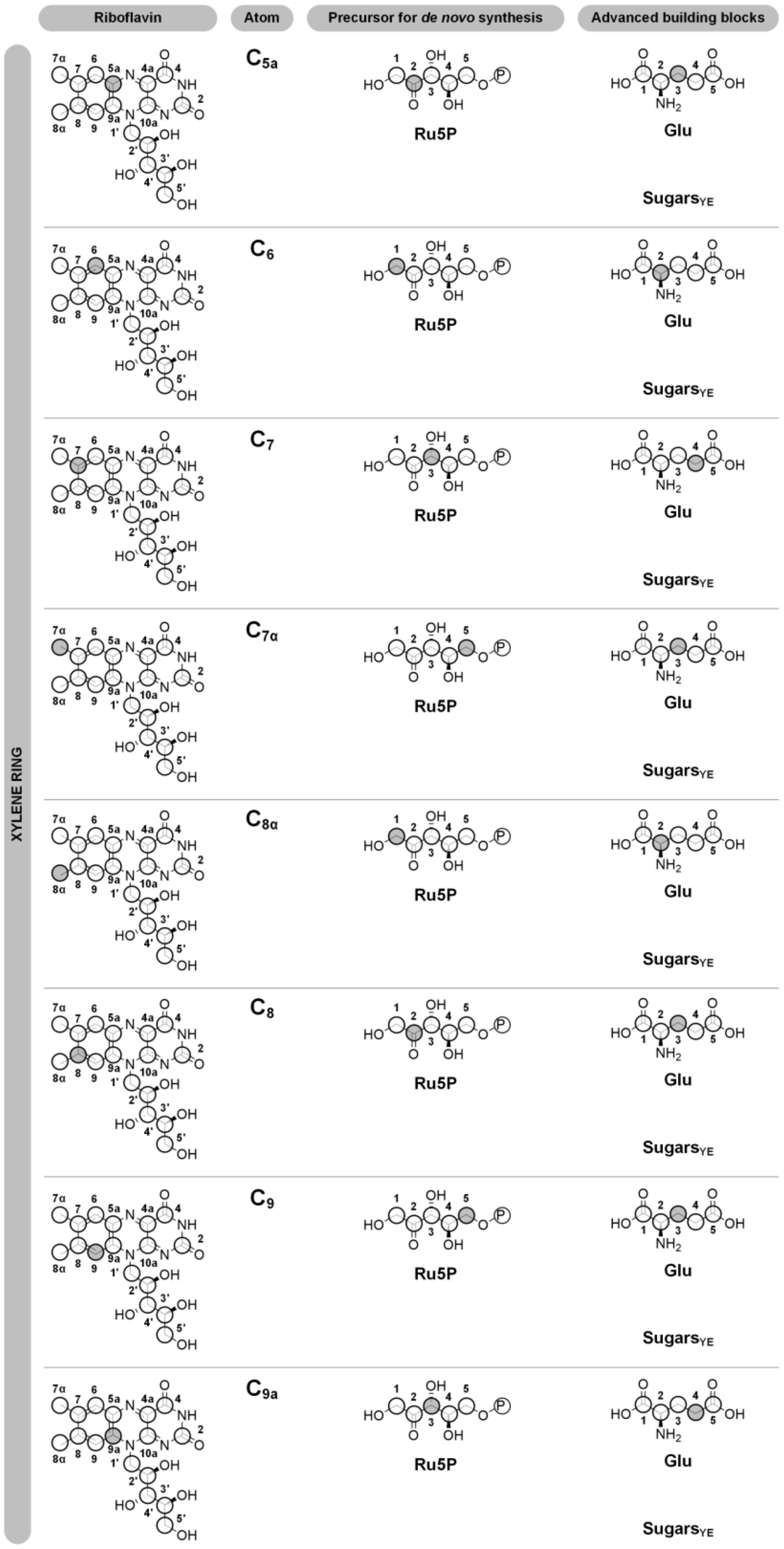

Figure S7: Metabolic origin of carbon atoms of the xylene ring in riboflavin. Grey circles denote the carbon atom of interest and its origin in the metabolic precursor or donor to the precursor. The numbering is specific for the molecule, therefore, e.g. carbon atom 2 of riboflavin does not equal carbon atom 2 of Ru5P. Glu, glutamate; Ru5P, ribulose 5-phosphate; Ser, serine; $Sugars_{YE}$, sugars derived from yeast extract.

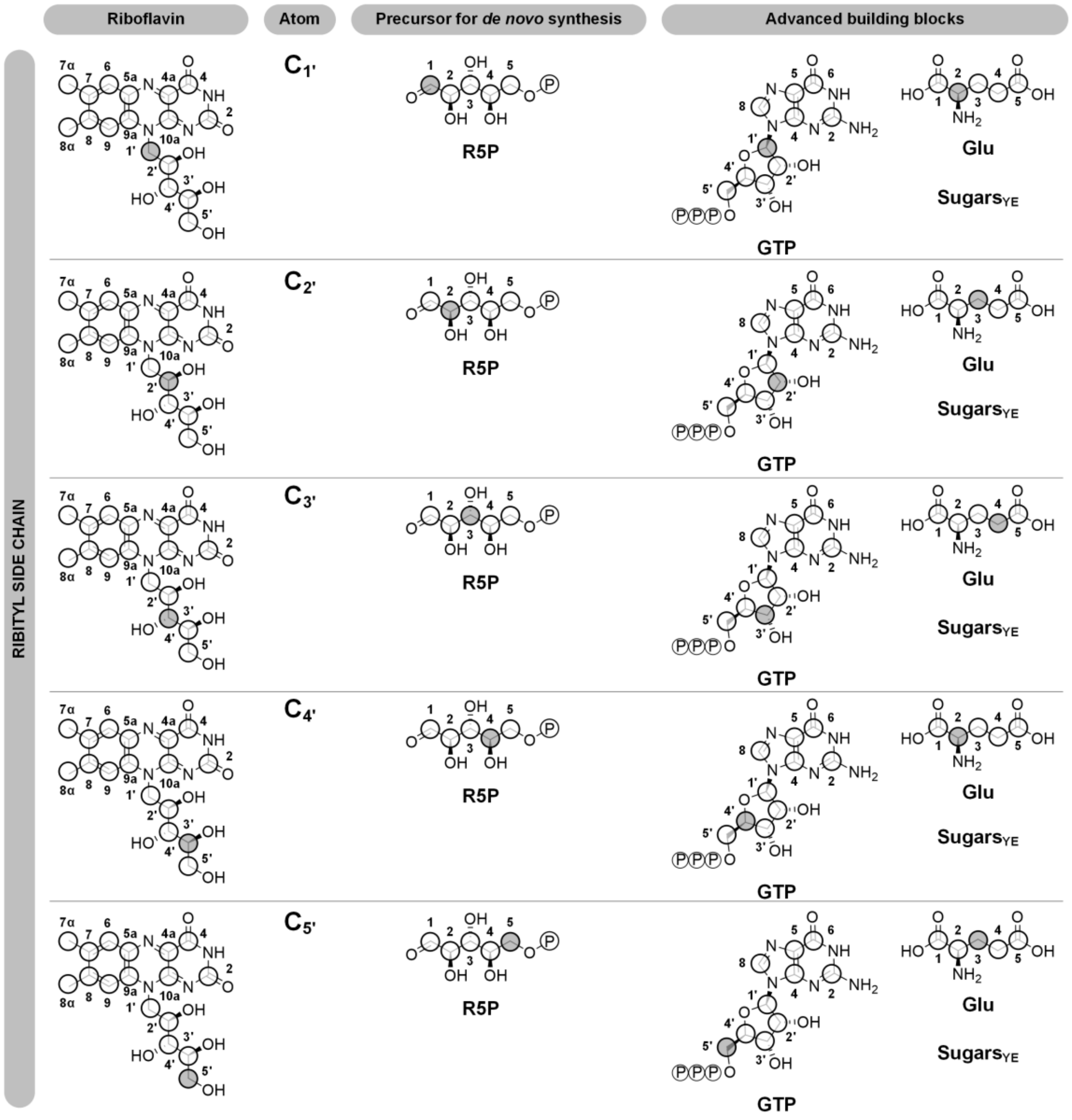

Figure S8: Metabolic origin of carbon atoms of the ribityl side chain of riboflavin. Grey circles denote the carbon atom of interest and its origin in the metabolic precursor or donor to the precursor. The numbering is specific for the molecule, therefore, e.g. carbon atom 2 of riboflavin does not equal carbon atom 2 of GTP. Glu, glutamate; GTP, guanosine triphosphate; R5P, ribose 5-phosphate; Sugars$_{YE}$, sugars derived from yeast extract.

Literature

1. Schwechheimer SK, Becker J, Peyriga L, Portais J-C, Sauer D, Müller R, Hoff B, Haefner S, Schröder H, Zelder O, Wittmann C. Improved riboflavin production with *Ashbya gossypii* from vegetable oil based on ^{13}C metabolic network analysis with combined labeling analysis by GC/MS, LC/MS, 1D, and 2D NMR. Metab Eng. 2018;47:357-373.
2. van Winden WA, Wittmann C, Heinzle E, Heijnen JJ. Correcting mass isotopomer distributions for naturally occurring isotopes. Biotechnol Bioeng. 2002;80:477-479.
3. Christensen B, Thykaer J, Nielsen J. Metabolic characterization of high- and low-yielding strains of *Penicillium chrysogenum*. Appl Microbiol Biotechnol. 2000;54:212-217.
4. Adler P, Frey LJ, Berger A, Bolten CJ, Hansen CE, Wittmann C. The key to acetate: metabolic fluxes of acetic acid bacteria under cocoa pulp fermentation-simulating conditions. Appl Environ Microbiol. 2014;80:4702-4716.
5. Ledesma-Amaro R, Kerkhoven EJ, Luis Revuelta J, Nielsen J. Genome scale metabolic modeling of the riboflavin overproducer *Ashbya gossypii*. Biotechnol Bioeng. 2014;111:1191-1199.
6. Stahmann KP, Kupp C, Feldmann SD, Sahm H. Formation and degradation of lipid bodies found in the riboflavin-producing fungus *Ashbya gossypii*. Appl Microbiol Biotechnol. 1994;42:121-127.
7. Bacher A, Eberhardt S, Fischer M, Kis K, Richter G. Biosynthesis of vitamin B_2 (riboflavin). Annu Rev Nutr. 2000;20:153-167.
8. Kanehisa M, Goto S. KEGG: kyoto encyclopedia of genes and genomes. Nucleic Acids Res. 2000;28:27-30.
9. Kanehisa M, Sato Y, Kawashima M, Furumichi M, Tanabe M. KEGG as a reference resource for gene and protein annotation. Nucleic Acids Res. 2016;44:17.
10. Kanehisa M, Furumichi M, Tanabe M, Sato Y, Morishima K. KEGG: new perspectives on genomes, pathways, diseases and drugs. Nucleic Acids Res. 2017;45.

11. Burns VW. Regulation and coordination of purine and pyrimidine biosyntheses in yeast: I. Regulation of purine biosynthesis and its relation to transient changes in intracellular nucleotide levels. Biophys J. 1964;4:151-166.
12. Rolfes RJ. Regulation of purine nucleotide biosynthesis: in yeast and beyond. Biochem Soc Trans. 2006;34:786-790.
13. Lacroute F. Regulation of pyrimidine biosynthesis in *Saccharomyces cerevisiae*. J Bacteriol. 1968;95:824-832.
14. Zhang JY, Reddy J, Buckland B, Greasham R. Toward consistent and productive complex media for industrial fermentations: Studies on yeast extract for a recombinant yeast fermentation process. Biotechnol Bioeng. 2003;82:640-652.
15. Kohlstedt M, Sappa PK, Meyer H, Maaß S, Zaprasis A, Hoffmann T, Becker J, Steil L, Hecker M, van Dijl JM, et al. Adaptation of *Bacillus subtilis* carbon core metabolism to simultaneous nutrient limitation and osmotic challenge: a multi-omics perspective. Environ Microbiol. 2014;16:1898-1917.
16. Lange A, Becker J, Schulze D, Cahoreau E, Portais J-C, Haefner S, Schröder H, Krawczyk J, Zelder O, Wittmann C. Bio-based succinate from sucrose: High-resolution ^{13}C metabolic flux analysis and metabolic engineering of the rumen bacterium *Basfia succiniciproducens*. Metab Eng. 2017;44:198-212.
17. Krömer JO, Sorgenfrei O, Klopprogge K, Heinzle E, Wittmann C. In-depth profiling of lysine-producing *Corynebacterium glutamicum* by combined analysis of the transcriptome, metabolome, and fluxome. J Bacteriol. 2004;186:1769-1784.
18. Bacher A, Rieder C, Eichinger D, Arigoni D, Fuchs G, Eisenreich W. Elucidation of novel biosynthetic pathways and metabolite flux patterns by retrobiosynthetic NMR analysis. FEMS Microbiol Rev. 1998;22:567-598.
19. Plaut GWE. Biosynthesis of riboflavin. 2. Incorporation of ^{14}C-labeled compounds into ring A. J Biol Chem. 1954;211:111-116.
20. Plaut GWE. Biosynthesis of riboflavin. 1. Incorporation of ^{14}C-labeled compounds into ring B and ring C. J Biol Chem. 1954;208:513-520.

21. Plaut GWE, Broberg PL. Biosynthesis of riboflavin. 3. Incorporation of ^{14}C-labeled compounds into the ribityl side chain. J Biol Chem. 1956;219:131-138.
